# Affordability of nutritious foods for complementary feeding in South Asia

**DOI:** 10.1093/nutrit/nuaa139

**Published:** 2021-03-08

**Authors:** Theresa Ryckman, Ty Beal, Stella Nordhagen, Zivai Murira, Harriet Torlesse

**Affiliations:** 1 Center for Health Policy and the Center for Primary Care and Outcomes Research, Department of Medicine, Stanford University School of Medicine, Stanford, California, USA; 2 Global Alliance for Improved Nutrition, Washington, DC, USA; 3 Department of Environmental Science and Policy, University of California, Davis, Davis, California, USA; 4 Global Alliance for Improved Nutrition, Geneva, Switzerland; 5 United Nations Children’s Fund, Regional Office for South Asia, Kathmandu, Nepal

**Keywords:** affordability, complementary feeding, dietary diversity, micronutrients, price

## Abstract

The high prevalence of stunting and micronutrient deficiencies among children in South Asia has lifelong health, educational, and economic consequences. For children aged 6–23 months, undernutrition is influenced by inadequate intake of complementary foods containing nutrients critical for growth and development. The affordability of nutrients lacking in young children’s diets in Bangladesh, India, and Pakistan was assessed in this study. Using data from nutrient gap assessments and household surveys, household food expenditures were compared with the cost of purchasing foods that could fill nutrient gaps. In all 3 countries, there are multiple affordable sources of vitamin A (orange-fleshed vegetables, dark leafy greens, liver), vitamin B_12_ (liver, fish, milk), and folate (dark leafy greens, liver, legumes, okra); few affordable sources of iron and calcium (dark leafy greens); and no affordable sources of zinc. Affordability of animal-source protein varies, with several options in Pakistan (fish, chicken, eggs, beef) and India (fish, eggs, milk) but few in Bangladesh (eggs). Approaches to reduce prices, enhance household production, or increase incomes are needed to improve affordability.

## INTRODUCTION

In 2017, malnutrition was estimated to contribute to >1 million premature deaths among children younger than 5 years in South Asia.^1^ Childhood stunting affects 1 in 3 children across the region, 15% of children are wasted, and micronutrient deficiencies are an enduring challenge.^2^ The highest burdens of malnutrition in South Asia are concentrated in Bangladesh, India, and Pakistan—the 3 most highly populated countries in the region. In these 3 countries, it is estimated that 18%–52% of children younger than 5 years are vitamin A deficient, 11%–44% are iron deficient, and 19%–45% are zinc deficient.^3^ Stunting, wasting, and micronutrient deficiencies in early life, especially during the first 1000 days between conception and a child’s second birthday, increase the risk of morbidity and mortality and lead to lower levels of physical and cognitive development that affect children throughout their lives.^4^

Although stunting prevalence has declined substantially over the past 2 decades, the burdens of stunting and micronutrient deficiencies remain high in South Asia.^5^ One of the main explanations is that complementary foods and feeding practices in early life are not supplying sufficient essential nutrients for healthy growth and development.^6^^,^^7^ In South Asia, only 20% of children aged 6–23 months consume a diet that meets minimum diversity requirements, only 25% consume nondairy animal source foods, and less than half of all children consume vegetables or fruits daily.^7^

Interventions to improve complementary feeding practices and increase dietary diversity have been shown to improve child growth.^8^^,^^9^ These interventions often focus on improving caregiver knowledge, behavior, and preferences for nutritious and diverse complementary foods through nutrition education and counselling. However, low dietary diversity may also be rooted in food availability, accessibility, and affordability barriers, as well as time and convenience constraints.^10^ In several studies, affordability has been identified as an important barrier to more nutritious food consumption and a key driver of food choice in many low- and middle-income countries.^11–19^ Minimum dietary diversity is correlated with household wealth in South Asian countries.^20^^,^^21^ Previous work has found that a minimum-cost nutritious diet would exceed current household food expenditure for most households in Bangladesh and Pakistan^14^^,^^16^ and that the least-cost diet to satisfy dietary recommendations is unaffordable to approximately half of poor rural households in India.^18^

Despite the evidence that affordability is a barrier to increasing consumption of nutritious complementary foods, the extent to which different nutrients, and foods that are good sources of those nutrients, are affordable in South Asian countries, and for which households, remains unknown. Identifying the lowest-cost foods to fill key nutrient gaps could aid in the design of complementary feeding interventions in the region, including those acting at the market level and those aiming to shape household demand. In addition, knowing which nutrients are already affordable to most households at current prices can help direct research into other barriers to consumption. More broadly, there is a need for additional methods that can be used to quantify affordability of nutrients or individual foods in terms of their nutrient content (E. Djimeu Wouabe, H. Kelahan, T. Beal, et al., unpublished data), because most food affordability literature instead focuses on the relative cost per kilocalorie^11^ or the cost of a fully nutritious diet.^12^^,^^22^^,^^23^ Once developed, such methods need to be tested across diverse settings for their usefulness and applicability.

In this study, we explored the affordability of nutrients and foods using available evidence on nutrient gaps among children of complementary feeding age (ie, 6–23 months) in South Asia. We assessed affordability at the individual nutrient, food, and household levels, which gave us the opportunity to assess not just country-level affordability but inequalities in affordability within countries. This work builds on a concurrent study using similar methods in Eastern and Southern Africa,^24^ demonstrating the study’s wide applicability as well as the local specificity of some of its results. Beyond assessing affordability of nutrients and foods for young children in South Asia, the methods used in this study could be applied to other countries and populations where diets are lacking in ≥1 key nutrients.

## METHODS

The analysis consisted of 3 major components: (1) an assessment of nutrient gaps in the diets of children aged 6–23 months; (2) an estimation of portion sizes of country-specific foods that could fill these nutrient gaps; and (3) an analysis of current household consumption of and expenditure on the foods and the extent to which households could potentially afford to increase consumption. The analysis was focused on 3 countries in South Asia: Bangladesh, India, and Pakistan. These countries were chosen because of their large populations (95% of the region’s total) and share of the region’s malnutrition burden, low levels of dietary diversity and micronutrient intake, government interest and commitment to addressing child malnutrition, and cultural and religious diversity.

### Nutrient gap assessment

The choice of nutrients to analyze for each country was informed by comprehensive nutrient gap assessments that were conducted for each country.^3^ These assessments enabled micronutrient gaps to be identified by synthesizing country-specific evidence on the prevalence among children aged 6–23 months of inadequate intakes or availability of 11 micronutrients (namely iron, zinc, vitamin B_12_, calcium, vitamin A, folate, iodine, vitamin B_1_, niacin, vitamin B_6_, and vitamin C) commonly lacking in young children’s diets.^25^ Based on the findings from the comprehensive nutrient gap assessments, the likely micronutrient gaps among children of complementary feeding age in these 3 countries are iron, vitamin A, calcium, zinc, folate, and vitamin B_12_. More details on how micronutrient gaps were assessed is available in a report of Beal et al.^26^ The analysis also included foods that could meet protein needs, focusing on animal sources because, unlike plant sources, they are known to include all essential amino acids in adequate quantities needed for proper child growth and development.^27^

### Food selection and portion analysis

The methods to identify foods in each country that could meet requirements for these 7 nutrients are described in more detail elsewhere.^24^ Briefly, foods were included if they met the following 3 criteria: (1) foods could meet 50% of nutrient needs, adjusting for the proportion of each nutrient needed for complementary feeding, refuse, and cooking yield, and considering portion sizes that could feasibly be consumed by a young child; (2) country-specific price data were available or could be estimated from surveys; and (3) foods were consumed by at least 10% of households in the country, based on consumption and expenditure surveys (Table S1 in the Supporting Information online). The analysis also included the liver of animals commonly consumed in the country (eg, chicken liver and either beef or sheep liver), even though consumption was not tracked in our surveys and thus it did not meet the third criterion. This was done because if the animal’s flesh is being consumed, the animal’s organs should be available (even if liver itself is not currently consumed by households), and because liver contains high concentrations of numerous micronutrients.

We calculated daily edible portion sizes that would provide 50% of daily protein and micronutrient requirements from complementary foods for each food and nutrient, using nutrient density data from food composition tables (Table S2 in the Supporting Information online), reference nutrient intake, and data on the proportion of each nutrient required from complementary feeding (Table S3 in the Supporting Information online).^28–35^ Weekly purchasable portion sizes corresponding to these daily edible portion sizes were then calculated by adjusting for refuse (from food composition tables) and cooking yield (Table S4 in the Supporting Information online).^36–41^

In each country, several types of dark-green leafy vegetables and legumes were considered, which can vary substantially in nutrient content. For these 2 categories of foods, median nutrient densities and average prices were used across several different varieties. More details are available elsewhere^24^ and in Tables S5 and S6 in the Supporting Information online. The portion sizes for each food and nutrient are listed in Table S7 in the Supporting Information online. We also analyzed the cost to meet 100% of energy requirements (450 kcal/day) for children aged 6–23 months for all foods included in the other analyses with energy density at least 0.8 kcal/g as well as for the lowest-cost, widely consumed, nutrient-poor staple in the country (rice or wheat flour).

### Cost estimation and affordability analysis

The analysis drew upon the latest available household consumption and expenditure surveys for each country to assess consumption and expenditure patterns for the selected nutritious foods among households with children of complementary feeding age.^42–44^ These surveys asked representative samples of households to recall their food consumption and expenditures over the past 7–30 days (Table S1 in the Supporting Information online). Expenditures included actual expenditures from purchases as well as the monetary value of food consumed from own production, in-kind contributions, and other sources. Nationally representative food price data were not collected in the surveys and were not publicly available from other sources. Prices were estimated by averaging household-reported expenditures divided by household-reported consumption, which yielded spatially and temporally specific prices that could be matched to a household’s sub-national region, rural or urban location, and the month the household was surveyed. In Bangladesh, published price data available for urban areas only also were incorporated.^45^^,^^46^ These estimates were validated against alternate sources (details are reported in Supporting Information online).

In the analysis, we used these price estimates and the portion sizes calculated for each food to estimate the cost to meet 50% of nutrient needs from each food. These costs were compared to household food expenditure (from all sources, including food eaten in and outside of the home) adjusted for household size and composition, which was done by dividing total food expenditure by the number of adult equivalents (AEQs) in the household. Adult equivalents are an estimate of a household’s total energy needs, based on each household member’s proportional energy requirement relative to that of an adult, and are commonly used in analyses of household diets.^47^^,^^48^ Food expenditure was chosen as a comparator because it allowed us to assess affordability in terms of whether purchasing a food would detract substantially from resources for food (or, if the cost of a single food exceeded food expenditure, nonfood resources) and because it has been correlated with food security in other settings.^24^ There is a lack of evidence on what a food could cost, as a proportion of baseline food expenditure, and be affordable to a household, so in our analysis, we used <10% of food expenditure per AEQ as a benchmark for affordability. Apart from non-nutritious staples, in early analyses of these 3 countries and for 6 countries in Eastern and Southern Africa, it was found that households in low- and middle-income countries tend to spend <5% of resources on single nutritious foods,^24^ so 10% is a reasonable but conservative upper bound.

We analyzed affordability for countries overall, for urban and rural areas within each country, and for households of varying socioeconomic status, defined by dividing households into quintiles on the basis of their food expenditure per AEQ. In the analysis, we did not view rural or urban setting and socioeconomic status as independent drivers of affordability differences (because rural households tend to spend less, on average) but rather we sought to demonstrate how affordability may vary for these subgroups. Several components of our analysis, including nutrient densities, were explored in sensitivity analysis. Although the base case analysis does not account for current household consumption of a food, this was also explored via sensitivity analysis.

### Average share of micronutrient requirements

We also assessed affordability by accounting for a food’s composition of all 6 commonly lacking micronutrients, because many foods contain several of the 6 micronutrients. To do this, a new metric, average share of micronutrient requirements, was developed. For a given quantity of a given food, average share of requirements is calculated as the proportion of a child’s micronutrient requirements from complementary foods that are met from consuming that food, averaged over all 6 micronutrients (details in Supporting Information online and Figures S1 and S2 in the Supporting Information online). We calculated quantities for each food required to achieve an average of one-third of requirements, which could correspond to that quantity providing a value between 100% of requirements for 2 of the 6 micronutrients and 33% of requirements for all 6 micronutrients (see Figure S3 in the Supporting Information online). We calculated the cost of purchasing these quantities (again divided by household food expenditure per AEQ) for all foods for which the daily quantity was <100 g—a quantity chosen because it is a reasonable complementary feeding meal size. Assumptions about nutrient densities, cooking yield, refuse, and daily micronutrient requirements were the same as in the by-nutrient affordability analysis. To assess affordability, the cost of these quantities was compared to one-third of household food expenditure per AEQ. The fraction one-third was chosen because the quantities are calculated to meet one-third of requirements for the priority micronutrients.

### Statistical analysis details

Data curation, cleaning, and analysis were conducted using Stata 15 and the *svy* family of commands, which account for weighting, clustering, stratification, and other complex survey design techniques used in the household surveys.^49^ Unless otherwise noted, the results presented in this article are reported as means with 95%CIs that propagate variation in prices and household expenditures but not uncertainty in portion sizes, which was explored separately via sensitivity analysis. The CIs were calculated in Stata assuming that the estimates follow a normal distribution parameterized by the population mean and linearized robust SEs estimated from the data.

## RESULTS

### Household consumption and expenditure patterns

**Figure 1:**
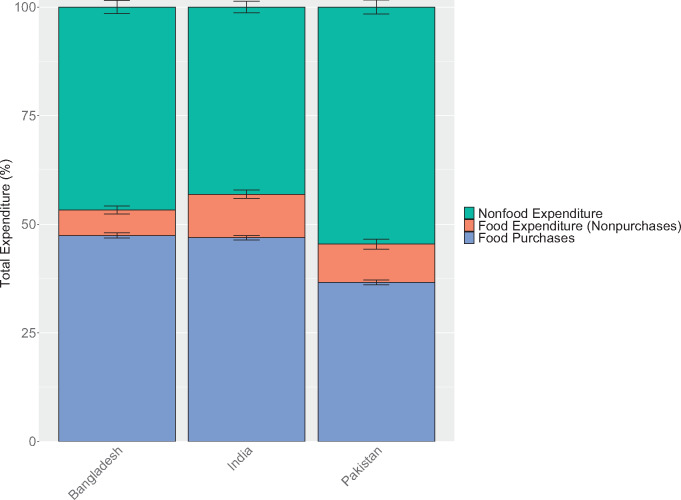
**Total household food and nonfood expenditures**. Note: Only data from households with children of complementary feeding age are shown, yielding sample sizes of 5813 households for Bangladesh, 10 868 households for India, and 5202 households for Pakistan. Error bars represent 95%CIs. Different surveys recorded household consumption over different periods: Bangladesh (2 weeks), India (1 week for fruits, vegetables, roots and tubers, and meats; 30 days for cereal products, legumes, and dairy products), Pakistan (2 weeks for most foods, 1 month for cereals and legumes), but we converted expenditures to weekly values in our analysis. Food expenditure includes expenditure on all foods, including those eaten outside of the home

In Bangladesh, India, and Pakistan, an average of 53%, 57%, and 45% of total household expenditures in each country, respectively, was spent on food, with the average Pakistani household spending proportionally less on food than the average Indian or Bangladeshi household (Figure 1). Most food came from purchases (82%–89%); even in rural areas, own production and other sources accounted for <25% of food expenditure (Figure S4). Across the 3 countries, households in the lowest food expenditure per AEQ quintile spent only 37%–45% as much on food as households in the highest quintile, and only 57%–66% as much as the average household, revealing substantial inequalities across households (Figure S5 in the Supporting Information online).

**Figure 2:**
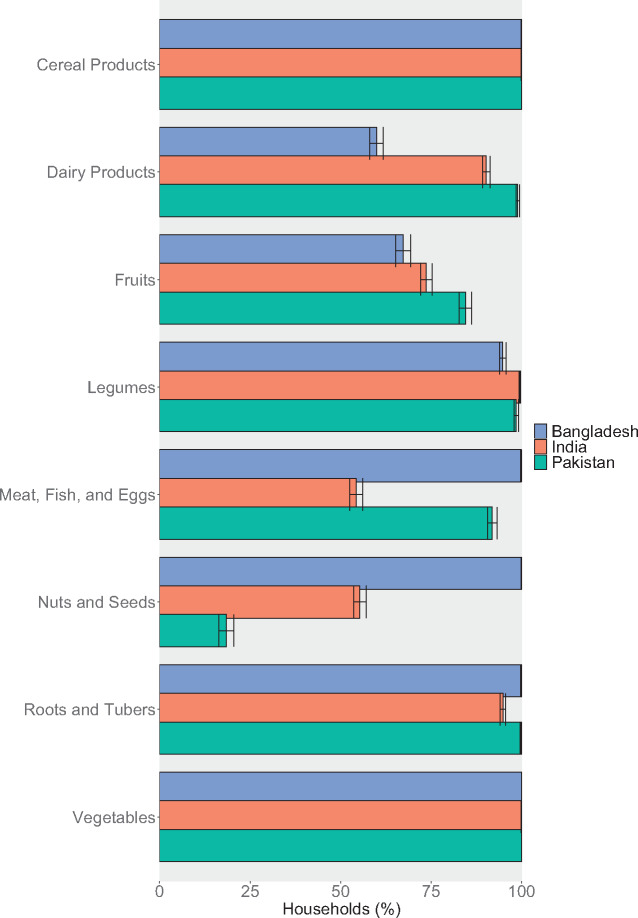
**Household consumption of key food groups**. Note: Only data from households with children of complementary feeding age are shown, yielding sample sizes of 5813 households in Bangladesh, 10 868 households in India, and 5202 households in Pakistan. Other food groups, including sugar and other sweets; oils and other fats; salt, spices, and condiments; beverages; and foods eaten outside of the home are omitted from this figure for brevity. Different surveys recorded household consumption over different periods: Bangladesh (2 weeks), India (1 week for fruits, vegetables, roots and tubers, and meats; 30 days for cereal products, legumes, and dairy products), Pakistan (2 weeks for most foods, 1 month for cereals and legumes). Error bars represent 95%CIs

**Figure 3:**
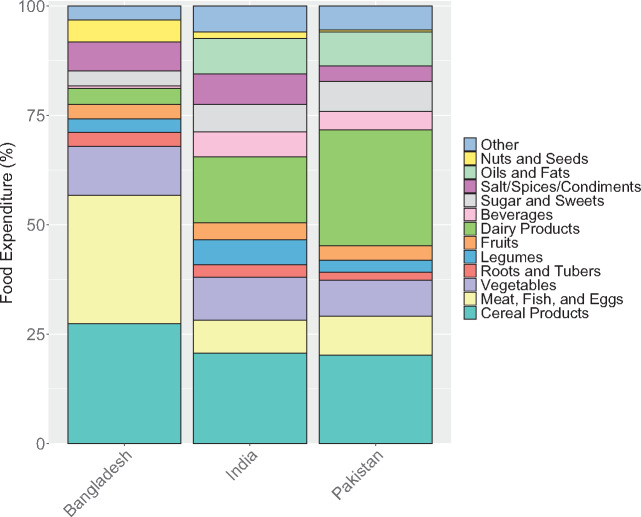
**Household expenditure by food group**. Note: Only data from households with children of complementary feeding age are shown, yielding sample sizes of 5813 households in Bangladesh, 10 868 households in India, and 5202 households in Pakistan. Expenditure includes value of consumption from all sources (purchases, own-production, and in-kind). Different surveys recorded household consumption over different periods: Bangladesh (2 weeks), India (1 week for fruits, vegetables, roots and tubers, and meats; 30 days for cereal products, legumes, and dairy products), Pakistan (2 weeks for most foods, 1 month for cereals and legumes), but we converted expenditures to weekly values in our analysis. Error bars represent 95%CIs. Food expenditure includes expenditure on all foods, including those eaten outside of the home (included in the “Other” category)

Household expenditure on and consumption of many food groups were similar across countries (Figures 2 and 3). The vast majority of households in Bangladesh, India, and Pakistan had consumed cereal products, legumes, roots and tubers, and vegetables over the past 7–30 days (time periods vary by survey and food; see Figure S1 in the Supporting Information online). On average, 21%–27% of household food expenditure was spent on cereal products and 8%–11% on vegetables. More than 90% of households in Bangladesh and Pakistan had consumed meat, fish, and/or eggs in the past 1–2 weeks, but only approximately 50% of households in India consumed these foods. Nuts and seeds were consumed much more commonly in Bangladesh than the other countries, and dairy products were more commonly consumed in Pakistan and India than in Bangladesh. Fruits were consumed by >50% of households in all 3 countries. These same patterns are observed in the expenditure data as well, with substantially more resources allocated toward meat, fish, and eggs in Bangladesh, and toward dairy products in India and Pakistan. Consumption was relatively consistent across urban and rural areas (Figures S6 and S7 in the Supporting Information online) and, for some food groups (namely, cereals, vegetables, legumes, and roots and tubers), across quintiles (Figures S8–S10 in the Supporting Information online). However, expenditure on and consumption of dairy products, meat, fish, eggs, fruits, and nuts and seeds was more varied, with low-quintile households spending and consuming less, especially for animal-source foods. This result suggests there are generally lower levels of dietary diversity among lower-resource households.

### Selected foods to meet nutrient needs

Several foods were included in the household surveys that were consumed by ≥10% of households (over 1–2 weeks for most foods) and could meet micronutrient or animal-source protein needs (Table 1). Many foods could fill multiple nutrient gaps and were consumed in at least 2 (or, in many cases, all 3) countries.

**Table 1:** Selected foods to meet animal-source protein and micronutrient needs^a^

	Bangladesh	India	Pakistan
Protein	Beef, chicken, fresh fish, eggs, fresh milk	Goat/mutton, chicken, fresh fish, eggs, yogurt, fresh milk	Beef, chicken, fresh fish, eggs, yogurt, fresh milk
Iron	Chicken liver, beef liver, beef, dark-green leafy vegetables, legumes	Chicken liver, sheep liver, goat/mutton, dark-green leafy vegetables, legumes	Chicken liver, beef liver, beef, dark-green leafy vegetables, legumes
Vitamin A	Beef liver, chicken liver, dark-green leafy vegetables, pumpkin, mango, eggs, fresh milk, fresh fish	Sheep liver, chicken liver, carrots, dark-green leafy vegetables, pumpkin, eggs, fresh milk, fresh fish, yogurt	Beef liver, chicken liver, carrots, dark-green leafy vegetables, eggs, fresh milk, fresh fish, yogurt
Calcium	Small fresh fish, dark-green leafy vegetables, fresh milk	Dark-green leafy vegetables, yogurt, fresh milk	Dark-green leafy vegetables, yogurt, fresh milk
Zinc	Beef, beef liver, chicken liver, chicken, legumes, eggs, fresh milk	Goat/mutton, sheep liver, chicken liver, groundnuts, chicken, legumes, eggs, yogurt, fresh milk	Beef, beef liver, chicken liver, groundnuts, chicken, legumes, eggs, yogurt, fresh milk
Folate	Chicken liver, beef liver, mango, dark-green leafy vegetables, okra, legumes, eggs, fresh peas, bananas	Chicken liver, sheep liver, groundnuts, dark-green leafy vegetables, okra, legumes, eggs, fresh peas, oranges, bananas	Chicken liver, beef liver, groundnuts, dark-green leafy vegetables, okra, legumes, fresh peas, eggs, oranges, bananas
Vitamin B_12_	Beef liver, chicken liver, fresh fish, beef, eggs, fresh milk	Sheep liver, chicken liver, fresh fish, goat/mutton, eggs, fresh milk, yogurt	Beef liver, chicken liver, fresh fish, beef, eggs, fresh milk, yogurt

a Foods are ordered from highest to lowest nutrient density within each nutrient category.

**Figure 4:**
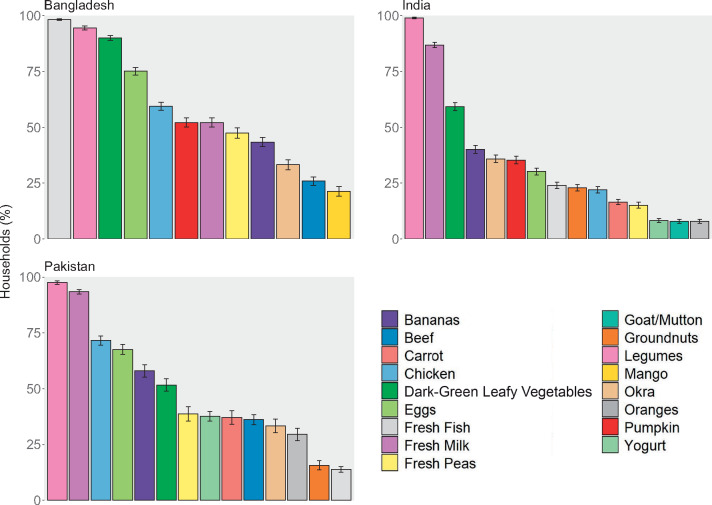
**Current consumption of selected nutritious foods**. Note: Only data from households with children of complementary feeding age are shown, yielding sample sizes of 5813 households in Bangladesh, 10 868 households in India, and 5202 households in Pakistan. Different surveys recorded household consumption over different periods: Bangladesh (2 weeks), India (1 week for fruits, vegetables, roots and tubers, and meats; 30 days for cereal products, legumes, and dairy products), Pakistan (2 weeks for most foods, 1 month for cereals and legumes). Error bars represent 95%CIs

**Figure 5:**
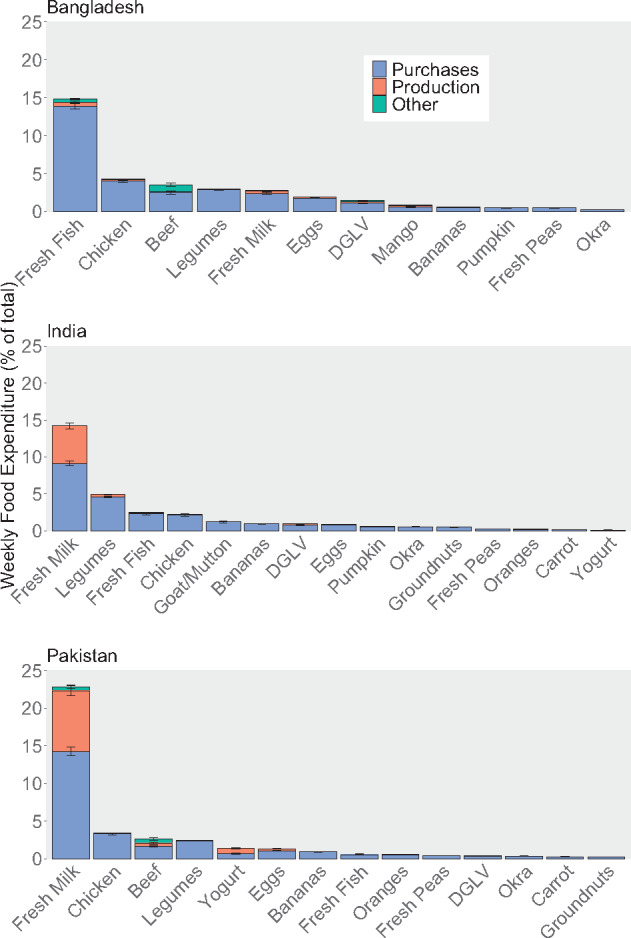
**Current expenditure from purchases, own production, and other sources for selected nutritious foods**. Note: Only data from households with children of complementary feeding age are shown, yielding sample sizes of 5813 households in Bangladesh, 10 868 households in India, and 5202 households in Pakistan. Different surveys recorded household consumption over different periods: Bangladesh (2 weeks), India (1 week for fruits, vegetables, roots and tubers, and meats; 30 days for cereal products, legumes, and dairy products), Pakistan (2 weeks for most foods, 1 month for cereals and legumes), but we converted expenditures to weekly values in our analysis. Error bars represent 95%CIs. DGLV, dark-green leafy vegetables

Across the 3 countries, there were some similarities in household consumption of and expenditure on these nutritious foods (Figures 4 and 5). Legumes, including lentils, moong beans, chickpeas, and split peas, were consumed by >90% of households in all countries. Milk was commonly consumed in India and Pakistan and stands out as the highest-expenditure food (accounting for >10% of total food spending in both countries) and the only food analyzed with substantial consumption from own production. Eggs and chicken were commonly consumed in Pakistan and Bangladesh and dark-green leafy vegetables were consumed by >50% of households in all 3 countries. Other foods varied more across countries; for example, fresh fish was consumed by almost all households in Bangladesh but <25% in India and Pakistan. Apart from milk in India and Pakistan and fish in Bangladesh, average expenditures for all foods analyzed were <5% of total food expenditure. Although consumption was similar across urban and rural areas within a country and across expenditure quintiles, most of the selected foods were consumed by slightly fewer rural households than urban households (Figures S11 and S12 in the Supporting Information online), and consumption and expenditure on several selected foods by lowest-quintile households were substantially lower than that of higher-quintile households (Figures S13 and S14 in the Supporting Information online). Rural and low-resource households consumed, on average, a smaller variety of the nutritious foods chosen for this analysis.

### Affordability of selected nutritious foods

**Figure 6:**
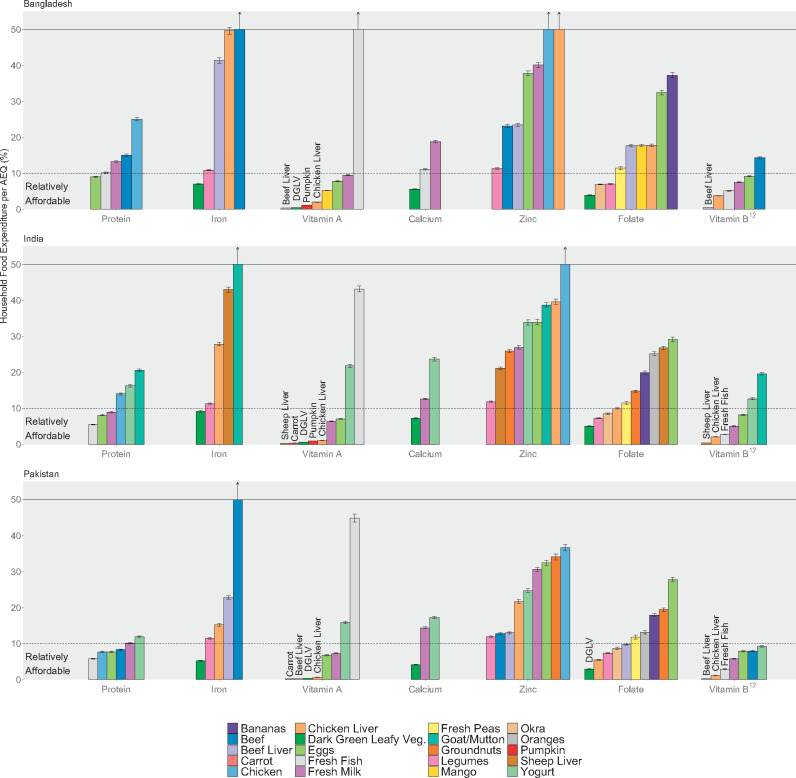
**Portion-size cost, as a share of total household food expenditure per adult equivalent (AEQ).** Note: The *y*-axis was truncated at 50%, but the costs of some foods exceeded 50% of household food expenditure per AEQ; these foods are designated with vertical arrows indicating that the bar continues vertically beyond the scale of the graph. Sample sizes were 5813 households (Bangladesh), 10 868 households (India), and 5,202 households (Pakistan). Error bars represent 95%CIs. DGLV, dark-green leafy vegetables

In all 3 countries, vitamin A and vitamin B_12_ appeared to be the most affordable nutrients, with several foods falling below 10%, or even 5%, of household food spending per AEQ on average (Figure 6). These foods included orange-fleshed vegetables (eg, carrots and pumpkin) and dark-green leafy vegetables for vitamin A, fresh milk for vitamin B_12_, and ruminant liver (beef liver in Bangladesh and Pakistan, sheep liver in India) and chicken liver for both vitamins. Dark-green leafy vegetables could also meet 50% of young children’s requirements for calcium and iron in Pakistan and folate in all 3 countries for <5% of food expenditure per AEQ, and it would cost <10% of food expenditure per AEQ to meet all 3 nutrient needs in all 3 countries with dark-green leafy vegetables (see Figure S15 in the Supporting Information online for results stratified by food). Several other foods that could fill folate gaps for <10% of food expenditure per AEQ; these vary by country (eg, legumes, okra, chicken liver). However, other options to meet iron and calcium needs would exceed 10% of food expenditure. Fresh fish, eggs, and milk in all 3 countries, as well as chicken and beef in Pakistan, cost close to the 10% threshold and could possibly be affordable options to meet protein needs for many households, although for Bangladesh, only eggs fall below 10% of household food expenditure per AEQ on average.

Zinc was the least affordable nutrient. Legumes were the most affordable source of zinc in all 3 countries and cost 11%–12% of food expenditure per AEQ, on average. Beef and beef liver were somewhat affordable sources of zinc in Pakistan (approximately 13% of food expenditure per AEQ).

**Figure 7:**
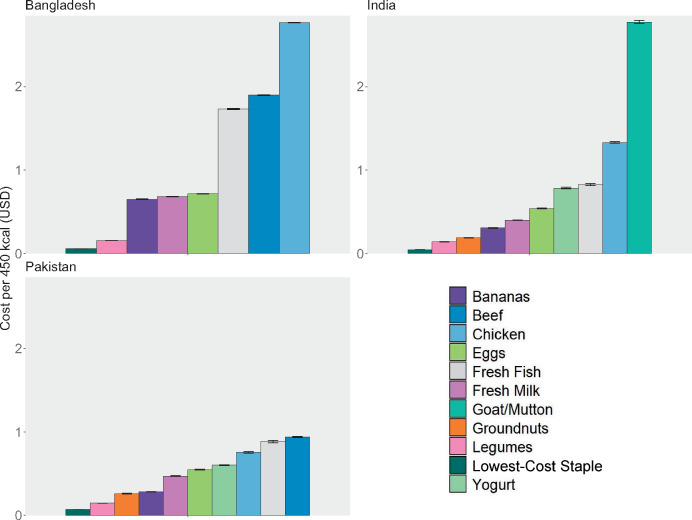
**Food cost per 450 kcal**. Note: The lowest-cost staple was rice in Bangladesh and Pakistan and wheat flour in India. Error bars represent 95%CIs

In general, animal-source foods were less affordable than plant-source foods. Chicken and ruminant meat (ie, beef, goat, or mutton) were often among the least affordable foods to fill nutrient gaps, with some exceptions (protein in Pakistan). The most affordable nutritious foods analyzed per kilocalorie were plant-source foods: legumes, groundnuts, and bananas (Figure 7). Even these most affordable foods cost between 2 and 10 times more than the lowest-cost staple (ie, rice or wheat flour), signaling the affordability challenges many households likely face in shifting consumption away from energy-dense and nutrient-poor staples toward more diverse nutritious foods (see also Figure S16 in the Supporting Information online). These findings, including the high cost of meeting many nutrient needs via most animal-source foods, were generally consistent in sensitivity analyses of nutrient densities and refuse (Figures S17 and 18 in the Supporting Information online), with some exceptions for specific foods. Most notably, if legumes contain less folate, on average, than was assumed in our primary analysis, they could potentially be a much less affordable source of folate.

**Figure 8:**
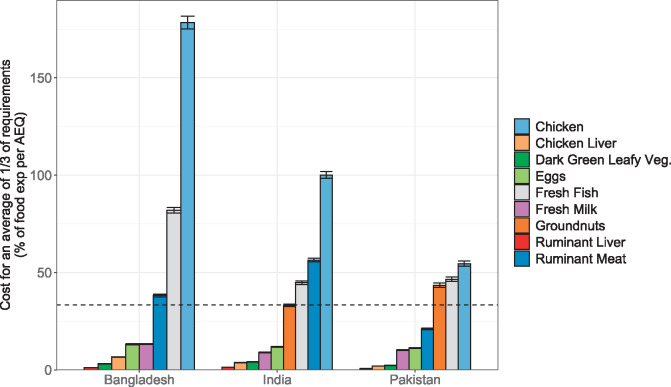
**Cost of achieving an average of one-third of micronutrient requirements, as a share of total household food expenditure per adult equivalent (AEQ)**. Sample sizes were 5813 households (Bangladesh), 10 868 households (India), and 5202 households (Pakistan). Error bars represent 95%CIs

In the average share of micronutrient requirements analysis, several animal-source foods appeared more affordable, given their high concentration of bioavailable nutrients. Affordability appeared more favorable for chicken liver, ruminant liver, fresh milk, and eggs in all 3 countries, as well as beef in Pakistan (Figure 8). Although several animal-source foods were considered in this analysis, only 2 plant-source foods could achieve an average of one-third of requirements at edible portion sizes ≤100 g: dark-green leafy vegetables and groundnuts (Table 2). Dark-green leafy vegetables were an affordable option to meet many individual nutrient needs and were also among the more affordable foods that could fulfill multiple micronutrient requirements in combination. Groundnuts were a less affordable plant-source option but were close to the 33% affordability threshold in India. Chicken and fresh fish were the least affordable animal-source foods by this analysis.

**Table 2:** Average share of micronutrient requirements portion sizes

Food^a^	Daily edible portion size required to achieve an average of one-third of requirements (g)
Ruminant liver (beef and sheep)	1
Chicken liver	3
Ruminant meat (beef)	27
Ruminant meat (mutton/goat)	28
Eggs	35
Dark-green leafy vegetables	57
Chicken	83
Fresh milk	95
Fresh fish	98
Groundnuts	99
Fresh peas	104
Yogurt	110
Legumes	138
Okra	161
Mango	174
Carrots	258
Oranges	292
Pumpkin	319
Bananas	694

a Foods with a portion size >100 g were not included in the average share of requirements analysis.

When we adjusted the analysis for estimates of the amount that households already spent on a food, some foods became substantially more affordable (because a smaller additional quantity of that food needed to be purchased to reach the portion size meeting 50% of nutrient requirements). At least 1 food had a net cost that would be <10% of current food expenditures per AEQ for all nutrients and countries (≥2 foods except for zinc in Bangladesh and India; Figure S19 in the Supporting Information online). In Bangladesh, household consumption of fish could already be more than enough to meet protein, calcium, and vitamin B_12_ needs. The same was true of milk in India and Pakistan (protein, vitamin A, calcium, vitamin B_12_). However, in our analysis, we were unable to account for which members of a household currently consume each food, which could explain how these nutrient gaps persist despite currently high levels of household consumption.

Subgroup analysis revealed that rural households would need to devote slightly larger proportions of total food expenditure per AEQ, on average, to purchasing nutritious foods than would urban households (Figure S20 in the Supporting Information online). More substantial inequalities were revealed when the affordability analysis was stratified by quintiles based on food expenditure per AEQ (Figure S21 in the Supporting Information online). For households in the lowest quintile, there were still multiple options to meet vitamin A and vitamin B_12_ requirements for <10% of food expenditure per AEQ, but dark-green leafy vegetables (folate, calcium, iron) and fresh fish (protein) were the only affordable foods to fill other nutrient gaps, and only in a subset of countries. These findings reflect lower baseline food expenditures rather than higher prices, because prices do not differ substantially across rural or urban settings or quintiles in these countries (Figures S22 and S23 in the Supporting Information online). With the exception of groundnuts in India, none of the average share of micronutrient requirements analysis findings differed by setting or quintile (Figures S24 and S25 in the Supporting Information online).

The subsamples of households with children of complementary feeding age in each country looked very similar to the full samples of households surveyed but had, on average, slightly lower food expenditures per AEQ and were slightly more likely to live in rural areas (Table S8 in the Supporting Information online). To check that the findings did not rely on the selection of only those households with complementary feeding–age children, which may not be a nationally representative sample of all households in the country, the main affordability analysis was run with all surveyed households. Under this analysis, conclusions about the relative and absolute affordability of each food and nutrient in each country were the same (Figure S26 in the Supporting Information online).

## DISCUSSION

This study undertook a comprehensive analysis of the affordability of different foods to meet the needs for 7 nutrients likely to be insufficiently consumed at present by young children in Bangladesh, India, and Pakistan. Vitamin A and vitamin B_12_ were the most affordable nutrients, with several foods, including orange-fleshed vegetables, dark leafy greens, and liver, falling below 10% of current household food expenditure per AEQ on average, even for low-resource households. Dark leafy greens proved to be a particularly affordable source of several individual micronutrients (namely, vitamin A, folate, calcium, and iron) for most households in all 3 countries and were also affordable when requirements for the 6 micronutrients were considered jointly. In addition, there were multiple other options (eg, legumes, okra) for meeting half of folate needs for <10% of average household expenditure per AEQ.

Desirability, convenience, cultural and religious acceptability, traditional recipes, and taste preferences may represent larger barriers than affordability to the intake of vitamin A, vitamin B_12_, and folate. For example, although dark leafy greens were consumed by nearly all households in Bangladesh, they were consumed by only 50%–60% of households in India and Pakistan. Inadequate knowledge, both of their nutritional value and of the importance of micronutrient consumption, could also play a role. Interventions could focus on these demand-side barriers to increase consumption of affordable sources of vitamin A, vitamin B_12_, and folate. Such efforts should be complemented with the promotion of appropriate household food-processing techniques and cooking practices to preserve nutrient content and increase the bioavailability of nutrients from these food sources. Although altering dietary practices can be challenging, there have been successful examples of improving complementary feeding practices through social and behavior change communication approaches in the region.^50–52^ In addition, for convenience or cultural reasons, households may prefer to purchase the same foods for all household members. Purchasing enough of these more affordable foods to feed the entire household would considerably increase their cost. Future research should explore these nonaffordability barriers to consumption of affordable sources of vitamin A, vitamin B_12_, and folate to identify context-specific policy solutions. It may also be that nutritious foods like meat cannot be purchased in small quantities in local markets, making them effectively unaffordable to households that do not have sufficient money to buy these large quantities. In such settings, finding ways to make such foods available in small quantities would be valuable, such as through private-sector business models that cater to the needs of the poor by making small serving sizes available for purchase.

There are also multiple animal-source foods that could meet protein needs in Pakistan and India for <10% of household expenditure per AEQ, but animal-source protein was less affordable in Bangladesh, where only eggs and fresh fish fell below this threshold, on average. Iron and calcium also presented greater affordability barriers, with dark-green leafy vegetables being the only determinably affordable source of these 2 nutrients across all 3 countries. For lowest-quintile households in Bangladesh and India, purchasing even these cheapest sources of protein, iron, and calcium in sufficient quantities would exceed 10% of food expenditure per AEQ. However, calcium and animal-source protein may be much more accessible in all countries than our primary analysis suggests, given the high current household consumption of fish in Bangladesh and milk in India and Pakistan revealed in sensitivity analysis (Figure S19 in the Supporting Information online). Furthermore, several animal-source foods (including milk, eggs, chicken liver, and beef liver) were among the lowest-cost options for meeting the requirements of the 6 commonly lacking micronutrients in combination.

Zinc had the greatest affordability barriers: Even the most affordable sources—legumes, ruminant meat, and ruminant liver—would generally cost >10% of food expenditure per AEQ (closer to 20% for households in the lowest spending quintile). Zinc was still the least affordable nutrient even after estimated current consumption was incorporated. Zinc has a relatively high cost in part because many zinc-rich foods are from animal sources, which tend to be considerably more expensive than plant-source foods. Interventions to address gaps in zinc intake especially, as well as iron and possibly calcium and animal-source protein, are thus unlikely to succeed if they do not address affordability, either through lowering prices (eg, by increasing availability or by providing subsidies) or through increasing incomes (eg, through social safety nets). Encouraging additional home production through livestock rearing could also help in some areas: With the exception of milk, none of these foods was commonly produced by households, and perishable foods (eg, animal-source foods) that are purchased often carry a price premium due to storage and transport difficulties. For those nutrients that can be provided via large-scale food fortification and/or biofortification (eg, iron, zinc, folic acid, vitamin B_12_, and vitamin A), such approaches can also be considered. Fortification could be especially promising where social protection programs that provide food in-kind or at subsidized prices (eg, India’s Public Distribution System) already exist and for unaffordable nutrients (eg, zinc and iron). Additional exploration of policy interventions that could make less-affordable foods and nutrients more accessible to households, such as social safety nets, promotion of home production, market interventions to lower prices, and fortification, should be prioritized for research.

The results of our study also highlight inequalities in household food expenditures and diet quality, with households in the lowest food expenditure per AEQ quintile spending considerably less than higher-quintile households, particularly on some of the most nutrient-dense animal-source foods. Poorer households are also more likely to rely on social protection schemes as a source of food and, if these programs primarily provide in-kind food assistance rather than cash transfers, may have less flexibility to shift food consumption than higher-income households. Thus, it is essential to ensure any interventions to improve affordability are well targeted to households facing the greatest barriers. Social protection interventions could be particularly beneficial to these households. Global and regional evidence indicates that social protection programs are most effective at improving outcomes related to child nutrition when combined with interventions to improve caregiver knowledge of and skills related to complementary feeding.^8^^,^^53^^,^^54^

Overall, the results of this study show similar profiles of nutrient affordability across the 3 countries, with a few differences. One major difference is that prices (and thus unaffordability) of animal-source foods tended to be higher in Bangladesh; this finding is consistent with a recent analysis of the relative prices of several foods groups compared to cereals across 4 South Asian countries.^21^ In addition, consumption of most animal-source foods is far less common in India than in Bangladesh and Pakistan, due in part to cultural differences, including a preference for vegetarian diets held among ≥20% of the population.^55^^,^^56^ Because we examined only sources that were consumed by at least 10% of households, some of this cultural sensitivity is already built in. For example, beef and beef liver were not considered in India because consumption was <10%. National-level analyses may mask subnational differences in cultural acceptability and food availability, including seasonal variation (see Figure S27 in the Supporting Information online for seasonal food price differences). Subnational analyses, using the methods discussed in this article, could generate context-specific evidence on which foods to promote across the year, as has been done with cost-of-diet analyses.^16^^,^^18^^,^^19^

This is the second of 2 analyses applying this novel method to estimate nutrient affordability. The first^24^ examined 6 countries in eastern and southern Africa. Comparing results across these very different regions reveals certain commonalities: Vitamin A is the most affordable nutrient in both regions, particularly through orange-fleshed vegetables, dark-green leafy vegetables, and liver. Vitamin B_12_ was only examined in 1 of the 6 African countries, but also proved to be a relatively affordable nutrient there. Similarly, iron and calcium were less affordable, and zinc was the least affordable nutrient in the 2 African countries where it was analyzed. However, there are also important differences. In particular, small dried or tinned fish were important affordable sources of protein, iron, and calcium in some of the African countries but were infrequently consumed in India and Pakistan, underscoring the importance of examining a food’s nutrient content as well as its regional availability and acceptability. Overall, this second application of the method helps demonstrate its applicability across a range of different contexts.

Our findings can also be compared with other literature on diet affordability in South Asia. Cost-of-diet analyses in all 3 countries have also identified dairy and other animal-source foods as presenting the greatest cost challenges and found that dark-green leafy vegetables are relatively more affordable.^14^^,^^16^^,^^18^^,^^19^ Focusing on children younger than 2 years, a cost-of-diet analysis in Pakistan also concluded that iron and calcium could present affordability challenges, but that vitamins A and B_12_ would also be unaffordable, whereas zinc would be affordable, which contrasts starkly with our findings.^16^ These differences are likely due, in part, to differences in the specific foods considered in the cost-of-diet analysis, which may be driven by an aim to identify a lowest-cost complete diet to meet the nutrient needs of all household members, rather than specific nutrients for children aged 6–23 months, as well as differences in price and nutrient composition data sources and methodological assumptions.

By analyzing affordability at the household level, our analysis captured within-country inequality in food and nutrient affordability; given the vast amount of data collected in the household expenditure surveys, the methods presented in this study could be extended to cover different subgroups. For example, some foods were affordable at the 10% threshold, on average, but exceeded 20% of current food expenditure per AEQ for lower-spending households, which may require special interventions that address those households’ limited resources for food, such as safety-net cash transfers or other interventions to increase incomes. This study’s broad approach can also be replicated across settings, allowing for comparisons across regions and countries, as we explored in this study, as well as over time. Furthermore, this is the first analysis of which we are aware that analyzed affordability separately for specific nutrients and foods, allowing for more targeted policy and programmatic interventions and future research efforts.

Attempts to set a threshold for affordability for nutrients and foods were limited, however, by the lack of existing benchmarks in the literature on nutrition affordability (E. Djimeu Wouabe, H. Kelahan, T. Beal, et al., unpublished data). We evaluated foods by whether their cost would exceed 10% of household food expenditure per AEQ (see Table S9 in the Supporting Information online for assessments under alternative thresholds). Although 10% appears to be a reasonable upper bound, based on current household spending on individual foods reported here and in other work,^24^ it is still somewhat arbitrary; furthermore, the proportion of food expenditure households can afford to reallocate may scale with current spending. This analysis was also based on the affordability of portion sizes that provide 50% of nutrient needs, assuming that the other 50% would be met by other foods that are currently part of the child’s diet. However, this may not be the case for all children. Importantly, none of these methodological decisions and thresholds affect the affordability of the foods and nutrients relative to one another. In addition, even without applying strict thresholds and after attempting to adjust for current consumption of a food, some nutrients and foods can clearly be distinguished as affordable (ie, vitamin A, vitamin B_12_, and folate; dark-green leafy vegetables, legumes, and liver) or unaffordable (ie, zinc and iron; chicken).

Similarly, although the one-third of food expenditure per AEQ threshold is also somewhat arbitrary in the average share of requirements analysis, many of the same foods are considered affordable for meeting both individual and multiple micronutrients (eg, liver, dark-green leafy vegetables, milk). However, there are some differences; these can generally be explained by foods’ nutrient compositions and not differences in the thresholds used for the 2 analyses. Eggs are more affordable when joint micronutrient densities are considered, because they are higher priced but have moderate amounts of most of the nutrients considered here, whereas legumes are more affordable for meeting individual nutrient needs because their cost is low but they contribute substantially to fewer micronutrient requirements (Figures S1–S3 in the Supporting Information online).

This study is also limited by a lack of recent price data that is representative of urban and rural areas in these countries and covers a large enough variety of foods. Prices that were estimated on the basis of household consumption and spending could be subject to noise in household reporting, including from recall and other biases.^57^ However, although these limitations may add random noise to the price estimates, which was somewhat reduced because averaged prices within subnational region and month were used, there is little reason to believe this noise would bias prices in 1 direction or the other. Comparison against external data sources and the similar affordability patterns observed across the 3 countries further validates our approach.

Also, in this study, foods were generally analyzed in broad categories (eg, legumes, dark leafy greens), whereas actual nutrient content may vary by specific food types, with implications for which foods are the most nutritious. For example, the sensitivity analysis indicated that if less folate-rich legumes were considered than assumed in the original analysis, legumes would become a much less affordable source of folate. Also, we considered in the analysis only foods that at least 10% of households consume regularly and thus may have omitted nutrient-dense foods that could potentially be included in diets. In addition, the analysis was based on the assumption that children were receiving certain nutrients from breastmilk or substitutes (ie, formula or animal milk); on the basis of data from demographic and health surveys, this assumption is likely to hold for most children in the countries studied here, especially those in the poorest households.^58–61^ Where it does not hold, a larger portion size would be required, entailing slightly reduced affordability.

Although the base case analysis did not consider current consumption of a food, household consumption of fish in Bangladesh and of milk in India and Pakistan, if divided according to number of AEQs in the household, was already large enough to meet requirements for several nutrients (ie, protein, calcium, vitamin B_12_) despite evidence indicating gaps in their consumption. This result emphasizes the importance of considering not just the purchase of a food at the household level but also the allocation of that food within the household, which could affect purchasing considerations and which this analysis could not explore. Such issues include, but go well beyond, affordability and also touch on decision-making roles, traditions, and sociocultural and gender norms that influence intrahousehold food allocations.^48^ Future research in this regard could focus on dairy and fish, because high current consumption levels may make these foods especially feasible complementary feeding options, but the presence of gaps in these nutrients points to other possible barriers to consumption. These findings also suggest there is a need for operational research to assess nonaffordability barriers and understand how to design interventions to improve the intake of affordable nutritious foods (eg, dark-green leafy vegetables, liver) and nutrients (eg, vitamins A and B_12_). Liver is another priority topic for future research because it is high in several micronutrients, but there were no data available on consumption of liver in the surveys. Research is also needed to understand how to improve the affordability of less affordable foods (eg, meat) or identify alternative options (eg, large-scale fortification, home fortification, or biofortification) for less-affordable nutrients (eg, zinc, iron), and to gather additional evidence on foods and nutrients with less-clear affordability conclusions (eg, calcium, animal-source protein).

## CONCLUSION

This study provides important evidence on which nutrients of concern in young children’s diets present affordability challenges in households in 3 South Asian countries and identifies the most affordable foods to meet likely nutrient gaps. Although several nutrients with affordability barriers are identified, the possibility of additional barriers beyond cost for any of the nutrients assessed here cannot be ruled out. More research is needed to inform the design of interventions that could address the high cost of zinc, iron, and calcium, and in Bangladesh, animal-source protein; the low consumption of affordable sources of vitamin A, vitamin B_12_, and folate among young children; or, especially for low-income households, increase household resources to purchase and/or produce foods rich in these nutrients, including through social protection mechanisms or subsidies. Ongoing government-led, multi-sector nutrition efforts in these 3 countries could use such research to develop more tailored interventions that help ensure nutritious foods are affordable to low-income households. By putting in place evidence-based interventions and identifying additional barriers to consumption, especially for more affordable nutrients (eg, vitamin A, vitamin B_12_, folate), nutrient gaps among children aged 6–23 months in the South Asia region, which accounts for >20% of the global population, can be better addressed, ultimately leading to improvements in the health and nutritional status of millions of children.

## Supplementary Material

nuaa139_Supplementary_Data

## Supporting Information

The following Supporting Information is available through the online version of this article at the publisher’s website.

*Table S1* **Data sources used for each country**

*Table S2* **Nutrient densities used in portion-size analysis**

*Table S3* **Nutrient requirements from complementary foods for children aged 6–23 months**

*Table S4* **Country-specific values for refuse, cooking yield, and proportion of purchasable portion**

*Table S5* **Variation in nutrient densities**

*Table S6* **Variation in food prices**

*Table S7* **Weekly purchasable portion sizes used in affordability analysis**

*Table S8* **Characteristics of all households surveyed and surveyed households with children of complementary feeding age**

*Table S9* **Nutrients and nutritious foods that are affordable and unaffordable under various thresholds**

*Figure S1* **Share of micronutrient requirements across portion sizes (plant-source foods)**

*Figure S2* **Share of micronutrient requirements across portion sizes (animal-source foods)**

*Figure S3* **Average share of micronutrient requirements across portion sizes, by food**

*Figure S4* **Total household food and nonfood expenditure, by rural or urban setting**

*Figure S5* **Total household food and nonfood expenditure, by quintile**

*Figure S6* **Household consumption of key food groups, by rural or urban setting**

*Figure S7* **Weekly household expenditure by food group and rural or urban setting**

*Figure S8* **Household consumption of key food groups, by quintile**

*Figure S9* **Proportional weekly household expenditure by food group and quintile**

*Figure S10* **A: Total weekly household expenditure by food group and quintile (local currency).** B: Total weekly household expenditure by food group and quintile (international dollars)

*Figure S11* **Current consumption of selected nutritious foods, by rural or urban setting**

*Figure S12* **Current expenditure on selected nutritious foods, by rural or urban setting**

*Figure S13* **Current consumption of selected nutritious foods, by quintile**

*Figure S14* **Current expenditure on selected nutritious foods, by quintile**

*Figure S15* **Portion-size cost, as a share of total household food expenditure per adult equivalent, by food**

*Figure S16* **Food prices per kilogram, by country, adjusted for currency exchange rates and purchasing-power parities**

*Figure S17* **Sensitivity analysis results for nutrient density and refuse for dark-green leafy vegetables, legumes, and chicken liver**

*Figure S18* **Cost/450 kcal sensitivity analysis results for nutrient density (legumes)**

*Figure S19* **Portion-size cost net current expenditure per adult equivalent (AEQ), as a share of total household food expenditure per AEQ**

*Figure S20* **Portion-size cost, as a share of total household food expenditure per adult equivalent, by rural or urban setting**

*Figure S21* **Portion-size cost, as a share of total household food expenditure per adult equivalent, by quintile**

*Figure S22* **Food cost/450 kcal by rural or urban setting**

*Figure S23* **Food cost/450 kcal by quintile**

*Figure S24* **Average share of micronutrient requirements affordability analysis, by rural or urban setting**

*Figure S25* **Average share of micronutrient requirements affordability analysis, by quintile**

*Figure S26* **Portion-size cost, as a share of total household food expenditure per adult equivalent, using all surveyed households**

*Figure S27* **A: Seasonal price variation by food and country: Bangladesh (taka).** B: Seasonal price variation by food and country: India (rupees). C: Seasonal price variation by food and country: Pakistan (rupees)
