# Supplementary material for: Affordability of nutritious foods for complementary feeding in South Asia
Source: Nutr Rev. 2021 Mar 8;79(Suppl 1):52–68. doi: 10.1093/nutrit/nuaa139 (PMC7948078; doi:10.1093/nutrit/nuaa139)
Supplement: nuaa139_Supplementary_Data [file nuaa139_supplementary_data.docx]

**SUPPORTING INFORMATION**

Contents

[Supplementary Methods – food and non-food expenditure definitions 3](#_Toc65487435)

[Supplementary Methods – details for each country 3](#_Toc65487436)

[Supplementary Methods – details for certain foods 4](#_Toc65487437)

[Supplementary Methods – average share of micronutrient requirements analysis 5](#_Toc65487438)

[Table S1: Data sources used for each country 7](#_Toc65487439)

[Table S2: Nutrient densities used in portion size analysis 8](#_Toc65487440)

[Table S3: Nutrient requirements from complementary foods for children aged 6-23 months 9](#_Toc65487441)

[Table S4: Country-specific values for refuse, cooking yield, and proportion of purchasable portion 10](#_Toc65487442)

[Table S5: Variation in nutrient densities 12](#_Toc65487443)

[Table S6: Variation in food prices 13](#_Toc65487444)

[Table S7: Weekly purchasable portion sizes used in affordability analysis 14](#_Toc65487445)

[Table S8: Characteristics of all households surveyed and surveyed households with children of complementary feeding age 15](#_Toc65487446)

[Table S9: Nutrients and nutritious foods which are affordable and unaffordable under various thresholds 16](#_Toc65487447)

[Figure S1: Share of micronutrient requirements across portion sizes (plant-source foods) 17](#_Toc65487448)

[Figure S2: Share of micronutrient requirements across portion sizes (animal-source foods) 18](#_Toc65487449)

[Figure S3: Average share of micronutrient requirements across portion sizes, by food 19](#_Toc65487450)

[Figure S4: Total household food and non-food expenditure, by rural/urban setting 20](#_Toc65487451)

[Figure S5: Total household food and non-food expenditure, by quintile 21](#_Toc65487452)

[Figure S6: Household consumption of key food groups, by rural/urban setting 22](#_Toc65487453)

[Figure S7: Weekly household expenditure by food group and rural/urban setting 23](#_Toc65487454)

[Figure S8: Household consumption of key food groups, by quintile 24](#_Toc65487455)

[Figure S9: Proportional weekly household expenditure by food group and quintile 25](#_Toc65487456)

[Figure S10A: Total weekly household expenditure by food group and quintile (local currency) 26](#_Toc65487457)

[Figure S10B: Total weekly household expenditure by food group and quintile (international dollars) 27](#_Toc65487458)

[Figure S11: Current consumption of selected nutritious foods, by rural/urban setting 28](#_Toc65487459)

[Figure S12: Current expenditure on selected nutritious foods, by rural/urban setting 29](#_Toc65487460)

[Figure S13: Current consumption of selected nutritious foods, by quintile 30](#_Toc65487461)

[Figure S14: Current expenditure on selected nutritious foods, by quintile 31](#_Toc65487462)

[Figure S15: Portion size cost, as a share of total household food expenditure per adult equivalent, by food 32](#_Toc65487463)

[Figure S16: Food prices per kg by country, adjusted for currency exchange rates and purchasing power parities 36](#_Toc65487464)

[Figure S17: Sensitivity analysis results for nutrient density and refuse for dark leafy green vegetables, legumes, and chicken liver 37](#_Toc65487465)

[Figure S18: Cost per 450 kcal sensitivity analysis results for nutrient density (legumes) 39](#_Toc65487466)

[Figure S19: Portion size cost net current expenditure per AEQ, as a share of total household food expenditure per AEQ 40](#_Toc65487467)

[Figure S20: Portion size cost, as a share of total household food expenditure per adult equivalent, by rural/urban setting 42](#_Toc65487468)

[Figure S21: Portion size cost, as a share of total household food expenditure per adult equivalent, by quintile 45](#_Toc65487469)

[Figure S22: Food cost per 450 kcal by rural/urban setting 48](#_Toc65487470)

[Figure S23: Food cost per 450 kcal by quintile 49](#_Toc65487471)

[Figure S24: Average share of micronutrient requirements affordability analysis, by rural/urban setting 50](#_Toc65487472)

[Figure S25: Average share of micronutrient requirements affordability analysis, by quintile 51](#_Toc65487473)

[Figure S26: Portion size cost, as a share of total household food expenditure per adult equivalent, using all surveyed households 52](#_Toc65487474)

[Figure S27A: Seasonal price variation by food and country – Bangladesh (taka) 54](#_Toc65487475)

[Figure S27B: Seasonal price variation by food and country – India (rupees) 55](#_Toc65487476)

[Figure S27C: Seasonal price variation by food and country – Pakistan (rupees) 57](#_Toc65487477)

[References 59](#_Toc65487478)

# Supplementary Methods – food and non-food expenditure definitions

Food expenditures (aggregate and for specific food items) are defined as the value of consumption for all foods and beverages (non-alcoholic and alcoholic) consumed by the household, both in the home and away from the home. This includes purchases, value of own production, and value of foods consumed from gifts and other in-kind sources.

Non-food expenditure includes expenditures on non-food items (including narcotics), services (including food processing service charges such as milling or butchering), healthcare, education (including some in-kind scholarships), housing (rent and imputed rent for households that own their property), insurance payments, taxes, and fees. Non-routine lumpy expenditures (such as for weddings) are included. House purchases, the value of durable goods owned by the household, and purchases for household enterprises were excluded.

Because data were collected over a relatively short period of time for each country, expenditures have not been adjusted for regional price differences or inflation, except when converted to common units (e.g., USD).

# Supplementary Methods – details for each country

Most of the methods described here were consistent across countries. However, there was some variation in the available price data and price estimation strategies used. These and other methodological differences between analyses for each country are summarized below.

*Bangladesh*

Bangladesh’s 2016-17 Household Income and Expenditure Survey (HIES) interviewed 46,080 households. Data on household members’ age in months was not available in the survey, and so the analysis was conducted for all households with children under two years. As part of the HIES, survey enumerators visited households on a daily basis for two weeks and asked them to report quantities of and expenditures on all foods consumed that day. Survey weights were not included in the survey datasets, so these were calculated manually as the inverse probability of a household being surveyed given the HIES’ stratified sampling strategy, based on the number of households sampled per primary sampling unit (PSU), the number of PSUS sampled per strata, and the total number of households per strata.

Price data stratified by time (month and year) and location (division) was obtained from publications available on the Bangladesh Bureau of Statistics website (Statistical Bulletins and Statistical Pocketbooks) ^1,2^. These data are only representative of urban areas. For rural areas, we estimated prices based on the urban price data and implied urban-rural price differentials calculated from the Household Income and Expenditure Survey. Specifically, we estimated prices for each item at the level of upazila/thana (a sub-unit of district) and urban/rural setting. If no households surveyed in a particular upazila consumed an item, we estimated its price at a higher level (e.g. district). For each item, we then calculated the difference between the average price in rural areas and the average price in urban areas. We multiplied the urban price data by this rural-urban difference to estimate rural prices. This approach yielded prices specific to urban/rural setting, division, and month.

*India*

India’s 2011 National Sample Survey (NSS, round 68, type 2 consumer expenditure) interviewed 101,651 households. Data on household members’ age in months was not available in the survey, and so the analysis was conducted for all households with children under two years. Households were asked to recall quantities consumed and expenditure on a long list of a foods over the past 7-30 days. The reference period was 30 days for cereal products, pulses/legumes, dairy products, sugar, and salt, and 7 days for fruits, vegetables, and meats.

We were unable to identify a reliable, representative source of data on market prices for India that covered a sufficient variety of foods. Instead, we relied on prices calculated from the National Sample Survey data by dividing expenditures by quantities of a food purchased. We estimated average prices for each food at the level of primary sampling unit (PSU) and month, across all households in that PSU that purchased that food in a given month. If no households in a particular PSU consumed a particular food in a particular month, we estimated prices at higher geographic levels (e.g. stratum, district, etc.) and broader time periods (e.g. three-month categories). We validated price estimate against historical crowd-sourced data from *numbeo.com* for a selection of overlapping items (milk, eggs, potatoes, chicken, and apples) ^3^.

*Pakistan*

Pakistan’s 2015-16 Household Income and Expenditure Survey (HIES) interviewed 24,238 households. Data on household members’ age in months was available, and so we conducted the analysis only for households with children aged 6-23 months. Households were asked to recall consumption (including quantities consumed and expenditures) from a long list of foods over the prior two weeks for most foods (consumption of cereals, pulses, oil, coffee, tea, and condiments was asked about for the prior month).

Similarly to India, we estimated prices from the Pakistan Household Income and Expenditure Survey data due to a lack of alternative data sources. Prices were again estimated as household-reported expenditures divided by reported quantities purchased. We estimated average prices for each food at the level of primary sampling unit (PSU) and month, across all households in that PSU that purchased that food in a given month. If no households in a particular PSU consumed a particular food in a particular month, we estimated prices at higher geographic levels (e.g. stratum, etc.) and broader time periods (e.g. three-month categories). Estimated prices were validated against data from the Pakistan Bureau of Statistics for a few overlapping items.

# Supplementary Methods – details for certain foods

For most foods, we exactly matched foods in the price data to foods in USDA and local nutrient composition tables ^4–6^ to estimate nutrient density, cooking yield, and refuse. To estimate current consumption and expenditure on these foods, if multiple similar varieties were covered in the survey data, we summed consumption/expenditure over these varieties for each household. For some foods, additional assumptions had to be made. These are detailed below:

*Dark Green Leafy Vegetables (DGLV)*

For most countries, surveys captured several different varieties of DGLV. Because nutrient composition can vary substantially across DGLV, we used median values across multiple varieties of DGLV in multiple food composition tables (Table S3) and varied nutrient densities (using the 10^th^ and 90^th^ percentile values) in sensitivity analysis (Figure S19).

*Legumes*

Legumes were treated similarly to DGLV. For nutrient density, we used the median value across multiple varieties (Table S3) and the 10^th^ and 90^th^ percentile values in sensitivity analysis (Figures S19-20). To estimate prices, we averaged prices across all legume varieties that were consumed by 10% or more of surveyed households (Table S4).

*Fish*

We included fresh fish as a source of protein, vitamin A, and vitamin B_12_ for all countries. For Bangladesh, we used the average price over several types of commonly consumed fish (puti, soal, rohi, and koi), while for Pakistan and India price data could not be estimated for multiple kinds of fish as the surveys only tracked consumption and expenditures on all types of fish combined. We also included small fresh fish (which tend to be higher in calcium) for Bangladesh only, as it is the only country where we could find evidence that small fish are commonly consumed by large parts of the population ^7^.

*Chicken*

Data on chicken nutrient content came from USDA food composition tables. We varied refuse based on whether price and expenditure data were for live chickens or chickens that had already been killed.

*Chicken Liver*

We used the price and refuse for chicken, assuming that households could obtain chicken liver from purchasing a whole chicken and that chicken liver is not sold separately. We also ran a sensitivity analysis with zero refuse, which applies to the scenario in which households can purchase chicken liver separately (Figures S19-20).

*Beef and Sheep Liver*

We used the price for beef (or in India, for goat/mutton), and zero refuse (assuming that households can purchase liver separately for around the same price as other cuts of meat).

# Supplementary Methods – average share of micronutrient requirements analysis

We developed the average share of micronutrient requirements metric to assess the affordability of a food in terms of its joint provision of multiple micronutrients. Our motivation for developing this metric was to more fairly assess the affordability of foods which are a good source of several micronutrients but may be considered less affordable when their individual micronutrient contributions are considered separately. For a given portion size *i,* of a given food *j,* the average share of requirements over a set of micronutrients *A,* is calculated as:

$$X_{i,j}=\frac{1}{|A|}\sum_{a \in A} min\{\frac{nutrient\_density_{a,j}*i}{nutrient\_requirements_{a}}, 1\}$$

For each food in the analysis, we calculated the portion size *i* for which average share of requirements equals one third, and assessed the affordability of those foods for which this portion size was less than or equal to 100 g. This calculation can be visualized in figures S1-S3.

We focused this analysis on the same set of six micronutrients (iron, vitamin A, calcium, zinc, folate, vitamin B_12_) chosen for the other analyses in this study and used the same assumptions about cooking yield and refuse (Table S5), nutrient requirements (which are adjusted for micronutrient contributions from breastmilk or formula; Table S2), and nutrient densities (Table S6).

# Table S1: Data sources used for each country

| **Country** | **Consumption & expenditure data source** | **Reference period** | **Time Period** | **Data collection method** | **Location of the list of foods covered** | **Food price data source** | **Notes** |
| --- | --- | --- | --- | --- | --- | --- | --- |
| Bangladesh | 2016-17 Household Income and Expenditure Survey (HIES) | 2 weeks | April 2016-March 2017 | Recall (daily) | Pages 30, 31, 44, and 59 of the survey questionnaire^8^ | Bangladesh Bureau of Statistics (annual statistical pocketbook and monthly statistical bulletins) and HIES | The Bureau of Statistics price data only cover urban areas, by division. We used this data directly for urban areas. For rural areas, we calculated the average urban-rural price differential for each food and applied this as a multiplier to the urban division-specific prices to estimate rural division-specific prices |
| India | 2011 National Sample Survey (NSS; round 68, type 2) | 1 week (fruits, vegetables, meats, all other foods); 1 month (cereal products, pulses, dairy products, sugar, salt) | July 2011-June 2012 | Recall | Pages 5-10 of the survey questionnaire^9^ | Estimated from the NSS | Prices estimated from the NSS were checked for validity for a selection of common items against crowdsourced food price data from numbeo.com ^3^ |
| Pakistan | 2015-16 Household Income and Expenditure Survey (HIES) | 2 weeks (most foods); 1 month (cereal products, pulses, oil, coffee, tea, condiments) | October 2015-August 2016 | Recall | Pages 8-14 of the survey’s female questionnaire^10^ | Estimated from the HIES | Prices estimated from the HIES were checked for validity for a selection of common items against data from the Pakistan Bureau of Statistics (which collects price data on fewer foods and for urban areas only) ^11^. |

# Table S2: Nutrient densities used in portion size analysis

| **Food** | **Iron (mg)** | **Vit A (mcg RAE)** | **Calcium (mg)** | **Zinc (mg)** | **Folate (mcg DFE)** | **Vit B12 (mcg)** |
| --- | --- | --- | --- | --- | --- | --- |
| Bananas | 0.3 | 3 | 5 | 0.2 | 20 | 0.0 |
| Beef | 2.8 | 0 | 8 | 6.5 | 8 | 2.6 |
| Beef Liver | 6.5 | 9,442 | 6 | 5.3 | 253 | 70.6 |
| Carrots | 0.3 | 852 | 30 | 0.2 | 14 | 0.0 |
| Chicken | 1.2 | 46 | 14 | 1.9 | 5 | 0.3 |
| Chicken Liver | 12.3 | 4,139 | 11 | 4.0 | 569 | 19.0 |
| DGLV | 2.7 | 367 | 151 | 0.6 | 54 | 0.0 |
| Eggs | 1.2 | 149 | 50 | 1.1 | 44 | 1.1 |
| Fresh Milk | 0.0 | 46 | 113 | 0.4 | 5 | 0.5 |
| Fresh Fish | 0.7 | 33 | 14 | 0.5 | 4 | 4.4 |
| Goat/Mutton | 4.2 | 0 | 14 | 5.6 | 5 | 2.8 |
| Groundnuts | 1.3 | 0 | 57 | 2.3 | 86 | 0.0 |
| Legumes | 2.6 | 1 | 34 | 1.3 | 45 | 0.0 |
| Mango | 0.5 | 152 | 13 | 0.1 | 33 | 0.0 |
| Okra | 0.3 | 14 | 77 | 0.4 | 46 | 0.0 |
| Oranges | 0.1 | 11 | 40 | 0.1 | 30 | 0.0 |
| Peas | 2.1 | 54 | 43 | 0.3 | 42 | 0.0 |
| Pumpkin | 0.6 | 288 | 15 | 0.2 | 9 | 0.0 |
| Sheep Liver | 6.8 | 13,682 | 7 | 6.5 | 73 | 67.0 |
| Yogurt | 0.1 | 27 | 121 | 0.6 | 7 | 0.4 |

# Table S3: Nutrient requirements from complementary foods for children aged 6-23 months

| **Nutrient** | **Requirement type** | **Animal or plant source*** | **Nutrient Requirements (by age)** | | | **Proportion of nutrient requirements needed from complementary foods**** |
| --- | --- | --- | --- | --- | --- | --- |
|  |  |  | **6-11 mo** | **12-35 mo** | **6-23 mo (estimated)** |  |
| Protein (g) | RDA | Animal source foods | 11.0 | 13.0 | 12.3 | 0.49 |
| Iron (mg) | RNI | Animal source foods | 6.2 | 3.9 | 4.7 | 0.98 |
| Iron (mg) | RNI | Plant source foods | 9.3 | 5.8 | 7.0 | 0.98 |
| Zinc (mg) | RNI | Animal source foods | 2.5 | 2.4 | 2.4 | 0.87 |
| Zinc (mg) | RNI | Plant source foods | 4.1 | 4.1 | 4.1 | 0.87 |
| Calcium (mg) | RNI | Both | 400.0 | 500.0 | 466.7 | 0.65 |
| Vitamin A (µg RAE) | RDA | Both | 500.0 | 300.0 | 366.7 | 0.17 |
| Vitamin B12 (µg) | RDA | Both | 0.5 | 0.9 | 0.8 | 0.70 |
| Folate (µg DFE) | RDA | Both | 80.0 | 150.0 | 126.7 | 0.60 |

RDA = Recommended Dietary Allowance, RNI = Recommended Nutrient Intake, RAE = Retinol Activity Equivalents, DFE = Dietary Folate Equivalents. Nutrient requirements are based on ^12^ for RDAs and (37) for RNIs. *For iron, we assumed 15% dietary iron bioavailability for animal-source foods and 10% for plant foods; for zinc we assumed 50% dietary zinc bioavailability for animal-source foods and 30% for legumes, nuts, and seeds ^13^. **Based on ^14^; we assumed that breast milk or formula provides the remaining proportion of nutrient requirements.

# Table S4: Country-specific values for refuse, cooking yield, and proportion of purchasable portion

| **Country** | **Food** | **Refuse from purchasable portion (proportion)*** | **Cooking yield (proportion)**** | **Proportion of purchasable portion** |
| --- | --- | --- | --- | --- |
| Bangladesh | Bananas | 0.36 | 1.00 | 0.64 |
|  | Beef | 0.15 | 0.70 | 0.60 |
|  | Beef liver | 0.00 | 0.73 | 0.73 |
|  | Chicken (live) | 0.49 | 0.77 | 0.39 |
|  | Chicken liver | 0.49 | 0.62 | 0.32 |
|  | Dark green leafy vegetables | 0.10 | 0.98 | 0.88 |
|  | Eggs | 0.12 | 1.01 | 0.89 |
|  | Fresh fish | 0.00 | 0.85 | 0.85 |
|  | Fresh fish (small - calcium) | 0.00 | 1.00 | 1.00 |
|  | Fresh milk | 0.00 | 1.00 | 1.00 |
|  | Fresh peas/beans | 0.06 | 1.00 | 0.94 |
|  | Legumes | 0.00 | 2.50 | 2.50 |
|  | Mango | 0.29 | 1.00 | 0.71 |
|  | Okra | 0.00 | 1.00 | 1.00 |
|  | Pumpkin | 0.30 | 0.92 | 0.64 |
| India | Bananas | 0.36 | 1.00 | 0.64 |
|  | Carrot | 0.11 | 0.94 | 0.84 |
|  | Chicken (live) | 0.49 | 0.77 | 0.39 |
|  | Chicken liver | 0.49 | 0.62 | 0.32 |
|  | Dark green leafy vegetables | 0.10 | 0.98 | 0.88 |
|  | Eggs | 0.12 | 1.01 | 0.89 |
|  | Fresh fish | 0.00 | 0.85 | 0.85 |
|  | Fresh milk | 0.00 | 1.00 | 1.00 |
|  | Fresh peas/beans | 0.06 | 1.00 | 0.94 |
|  | Goat/mutton | 0.34 | 0.70 | 0.46 |
|  | Groundnuts | 0.10 | 1.00 | 0.90 |
|  | Legumes | 0.00 | 2.50 | 2.50 |
|  | Okra | 0.00 | 1.00 | 1.00 |
|  | Oranges | 0.27 | 1.00 | 0.73 |
|  | Pumpkin | 0.30 | 0.92 | 0.64 |
|  | Sheep liver | 0.00 | 0.73 | 0.73 |
|  | Yogurt | 0.00 | 1.00 | 1.00 |
| Pakistan | Bananas | 0.36 | 1.00 | 0.64 |
|  | Beef | 0.15 | 0.70 | 0.60 |
|  | Beef liver | 0.00 | 0.73 | 0.73 |
|  | Carrot | 0.11 | 0.94 | 0.84 |
|  | Chicken (dead) | 0.34 | 0.77 | 0.51 |
|  | Chicken liver | 0.34 | 0.62 | 0.41 |
|  | Dark green leafy vegetables | 0.10 | 0.98 | 0.88 |
|  | Eggs | 0.12 | 1.01 | 0.89 |
|  | Fresh fish | 0.00 | 0.85 | 0.85 |
|  | Fresh milk | 0.00 | 1.00 | 1.00 |
|  | Fresh peas/beans | 0.06 | 1.00 | 0.94 |
|  | Groundnuts | 0.10 | 1.00 | 0.90 |
|  | Legumes | 0.00 | 2.50 | 2.50 |
|  | Okra | 0.00 | 1.00 | 1.00 |
|  | Oranges | 0.27 | 1.00 | 0.73 |
|  | Yogurt | 0.00 | 1.00 | 1.00 |

Notes: if item is consumed raw (e.g. fresh milk) then a value of 1.00 was used for cooking yield. Variation in refuse and cooking yields for the same item across countries reflects differences in the form in which the item was purchased (e.g. some surveys recorded prices for live chickens vs. dead chickens). See supplementary methods for more details. * Refuse values are from ^4,15^. ** Cooking yield values are from ^16,17^.

# Table S5: Variation in nutrient densities

| **Dark green leafy vegetables** | | | | | |
| --- | --- | --- | --- | --- | --- |
| **Food** | **Food composition table (Source)** | **Nutrient content** | | | |
|  |  | **Iron (mg)** | **Vitamin A (µg RAE)** | **Calcium (mg)** | **Folate**  **(µg DFE)** |
| Amaranth leaves | Bangladesh FCT ^6^ | 6.4 | 816 | 240 | 50 |
| Indian spinach |  | 2.2 | 201 | 140 | 92 |
| Spinach |  | 3.1 | 672 | 157 | 177 |
| Average of 20 varieties | Tamil Nadu, India (Amalraj & Pius ^5^) | - | - | 325 | - |
| Amaranth leaves | USDA FCT ^4^ | 2.3 | 139 | 209 | 57 |
| Pumpkin leaves |  | 3.2 | 80 | 43 | 25 |
| Drumstick leaves |  | 2.3 | 351 | 151 | 23 |
| Spinach |  | 3.6 | 524 | 136 | 146 |
| Beet greens |  | 1.9 | 383 | 114 | 14 |
| Median [10^th^ and 90^th^ percentiles] | | 2.7 [2.1-4.4] | 367 [121-715] | 151 [100-257] | 54 [20-155] |

| **Legumes** | | | | | |
| --- | --- | --- | --- | --- | --- |
| **Food** | **Food composition table (Source)** | **Nutrient content** | | | |
|  |  | **Iron (mg)** | **Zinc (mg)** | **Folate (µg DFE)** | **Energy (kcal)** |
| Bengal gram (chickpea) | Bangladesh FCT ^6^ | 4.0 | 1.3 | 48 | 182 |
| Lentil |  | 2.2 | 1.7 | 9 | 155 |
| Grass pea |  | 1.9 | 1.2 | 42 | 142 |
| Pea |  | 2.3 | 1.6 | 9 | 170 |
| Green gram (mung bean) |  | 2.9 | 1.1 | 32 | 161 |
| Lentil | USDA FCT ^4^ | 3.3 | 1.3 | 181 | 114 |
| Pigeon pea |  | 1.1 | 0.9 | 111 | 121 |
| Chickpea |  | 2.9 | 1.5 | 172 | 164 |
| Median [10^th^ and 90^th^ percentiles] | | 2.6 [1.7-3.5] | 1.3 [1.0-1.6] | 45 [9-175] | 158 [119-174] |

Notes: FCT = Food Composition Table, USDA = US Department of Agriculture, RAE = Retinol Activity Equivalents, DFE = Dietary Folate Equivalents.

# Table S6: Variation in food prices

| **Country** | **Food (as listed in data)** | **Price per kg, local currency* [95% CI]** | **Price per kg, 2018 USD [95% CI]** |
| --- | --- | --- | --- |
| **Dark green leafy vegetables** | | | |
| Bangladesh | Spinach (palong, pui) | 24.1 [23.8-24.3] | 0.322 [0.318-0.325] |
| India | Palak/leafy veg. | 17.5 [17.2-17.8] | 0.380 [0.373-0.386] |
| Pakistan | Spinach | 23.6 [23.1-24.1] | 0.216 [0.211-0.221] |
| **Legumes** | | | |
| Bangladesh | Masur | 138.0 [137.4-138.6] | 1.844 [1.836-1.851] |
|  | Kesari | 68.9 [68.5-69.4] | 0.921 [0.914-0.928] |
|  | Gram (Chhola) | 82.4 [82.1-82.7] | 1.101 [1.096-1.105] |
|  | Moong bean | 109.0 [108.4-109.5] | 1.456 [1.448-1.463] |
|  | Mashkalai | 112.2 [111.8-112.7] | 1.499 [1.494-1.505] |
| India | Arhar/tur/pigeon pea | 64.7 [64.4-65.0] | 1.402 [1.395-1.409] |
|  | Gram: split | 47.9 [47.6-48.3] | 1.039 [1.032-1.046] |
|  | Gram: whole | 46.8 [46.4-47.3] | 1.015 [1.006-1.025] |
|  | Moong | 67.9 [67.5-68.3] | 1.471 [1.462-1.480] |
|  | Masur/red/brown lentils | 56.2 [55.8-56.5] | 1.218 [1.210-1.226] |
|  | Urd/black lentils | 60.1 [59.7-60.5] | 1.303 [1.295-1.312] |
|  | Peas | 41.7 [41.2-42.1] | 0.903 [0.893-0.914] |
|  | Khesari/grass pea | 40.6 [40.2-41.0] | 0.879 [0.870-0.888] |
| Pakistan | Pulse masoor (whole/broken) | 150.4 [149.3-151.6] | 1.374 [1.364-1.385] |
|  | Pulse moong (whole/broken) | 155.4 [154.4-156.4] | 1.420 [1.410-1.429] |
|  | Pulse mash (whole/broken/wash) | 188.3 [186.0-190.6] | 1.721 [1.700-1.742] |
|  | Pulse gram | 113.7 [112.7-114.7] | 1.038 [1.029-1.048] |
|  | Gram whole (black/white) | 119.0 [118.1-119.9] | 1.087 [1.079-1.096] |
|  | Beans (lobia red and white) | 131.9 [130.6-113.2] | 1.205 [1.193-1.217] |
| **Fish** | | | |
| Bangladesh | Rohi | 317.3 [315.1-319.4] | 4.239 [4.210-4.268] |
|  | Koi | 403.1 [400.4-405.8] | 5.386 [5.350-5.422] |
|  | Soal | 450.7 [449.0-452.5] | 6.022 [5.999-6.045] |
|  | Puti | 255.5 [254.6-256.5] | 3.414 [3.402-3.427] |
| India | Fish & prawns | 102.8 [101.5-104.1] | 2.228 [2.200-2.256] |
| Pakistan | Fish fresh | 261.6 [257.6-265.5] | 2.390 [2.353-2.426] |

*Local currencies are 2016-17 Bangladeshi Taka (BDT), 2011 Indian Rupees (INR), 2015-16 Pakistani Rupees (PKR).

#

# Table S7: Weekly purchasable portion sizes used in affordability analysis

|  | Protein | Iron | Vitamin A | Calcium | Zinc | Folate | Vitamin B_12_ |
| --- | --- | --- | --- | --- | --- | --- | --- |
| Bananas |  |  |  |  |  | 2.08 |  |
| Beef | 0.12 | 0.96 |  |  | 0.19 |  | 0.12 |
| Beef Liver |  | 0.34 | 0.003 |  | 0.19 | 0.14 | 0.004 |
| Carrots |  |  | 0.03 |  |  |  |  |
| Chicken* | 0.16-0.21 |  |  |  | 0.77-0.99 |  |  |
| Chicken Liver* |  | 0.32-0.41 | 0.01-0.02 |  | 0.45-0.59 | 0.11-0.15 | 0.02-0.03 |
| DGLV |  | 1.00 | 0.07 | 0.80 |  | 0.56 |  |
| Eggs | 3.78 |  | 3.29 |  | 15.86 | 13.60 | 3.84 |
| Fresh Fish | 0.11 |  | 3.29 | 0.11** |  |  | 0.05 |
| Fresh Milk | 0.64 |  | 0.46 | 0.91 | 1.94 |  | 0.36 |
| Fresh Peas |  |  |  |  |  | 0.67 |  |
| Goat/Mutton | 0.15 | 0.82 |  |  | 0.29 |  | 0.15 |
| Groundnuts |  |  |  |  | 0.60 | 0.34 |  |
| Legumes |  | 0.37 |  |  | 0.38 | 0.24 |  |
| Mango |  |  | 0.19 |  |  | 0.66 |  |
| Okra |  |  |  |  |  | 0.58 |  |
| Oranges |  |  |  |  |  | 9.27 |  |
| Pumpkin |  |  | 0.12 |  |  |  |  |
| Sheep Liver |  | 0.32 | 0.002 |  | 0.16 | 0.20 | 0.003 |
| Yogurt | 0.60 |  | 0.81 | 0.88 | 1.26 |  | 0.47 |

Notes: DGLV = Dark Green Leafy Vegetables. Units are based on units from the expenditure and price data and are in kilograms except for eggs (number of eggs), fresh milk (liters), and oranges (number of oranges)

*Chicken and chicken liver portion sizes vary depending on the purchasable form (alive vs. dead).

**Fresh fish was only included as a source of calcium for Bangladesh, where there is greater consumption of small fish that are higher in calcium.

# Table S8: Characteristics of all households surveyed and surveyed households with children of complementary feeding age

|  | All households  (full sample) | Household with children of complementary feeding age | P-value |
| --- | --- | --- | --- |
| Bangladesh | | | |
| Number of households | 45,990 | 5,813 |  |
| Percent rural | 65.80 [65.78-65.83] | 67.39 [66.00-68.79] | 0.03 |
| Mean annual food expenditure per AEQ | 23130 [22799-23461] | 21955 [21487-22422] | < 0.01 |
| Percent in lowest food expenditure per AEQ quintile | 20.0 [19.1-20.9] | 21.4 [19.8-22.9] | 0.03 |
| Mean household size | 4.03 [4.01-4.05] | 4.99 [4.93-5.04] | < 0.01 |
| India | | | |
| Number of households | 101,651 | 10, 868 |  |
| Percent rural | 68.75 [67.87-69.64] | 73.30 [71.76-74.84] | < 0.01 |
| Mean annual food expenditure per AEQ | 13177 [12967-13387] | 11517 [11315-11720] | < 0.01 |
| Percent in lowest food expenditure per AEQ quintile | 20.0 [19.5-20.5] | 22.7 [21.1-24.2] | < 0.01 |
| Mean household size | 4.42 [4.39-4.45] | 6.01 [5.93-6.10] | < 0.01 |
| Pakistan | | | |
| Number of households | 24,238 | 6,124 |  |
| Percent rural | 63.41 [62.39-64.42] | 67.71 [66.18-69.23] | < 0.01 |
| Mean annual food expenditure per AEQ | 31044 [30469-31618] | 28119 [27601-28638] | < 0.01 |
| Percent in lowest food expenditure per AEQ quintile | 20.0 [19.1-21.0] | 23.8 [22.1-25.6] | < 0.01 |
| Mean household size | 6.31 [6.42-6.38] | 7.84 [7.71-7.98] | < 0.01 |

This table shows weighted means and p-values corresponding to a two-tailed t-test comparing differences in weighted means (and accounting for survey clustering and sampling) between households with children of complementary feeding age and households without children of complementary feeding age.

# Table S9: Nutrients and nutritious foods which are affordable and unaffordable under various thresholds

|  | **Protein** | **Iron** | **Vitamin A** | **Calcium** | **Zinc** | **Folate** | **Vitamin B_12_** |
| --- | --- | --- | --- | --- | --- | --- | --- |
| **Affordable (at a threshold of less than 5% of total adjusted household food expenditure)** | | | | | | | |
| **Bangladesh** |  |  | Beef liver, DGLV, pumpkin, chicken liver |  |  | DGLV | Beef liver, chicken liver |
| **India** |  |  | Sheep liver, carrot, DGLV, pumpkin, chicken liver |  |  | DGLV | Sheep liver, chicken liver, fresh fish, fresh milk |
| **Pakistan** |  |  | Carrot, beef liver, DGLV, chicken liver | DGLV |  | DGLV | Beef liver, chicken liver, fresh fish |
| **Affordable (at a threshold of less than 10% of total adjusted household food expenditure)** | | | | | | | |
| **Bangladesh** | Eggs | DGLV | Beef liver, DGLV, Pumpkin, chicken liver, mango, eggs, fresh milk | DGLV |  | DGLV, okra, legumes | Beef liver, chicken liver, fresh fish, fresh milk, eggs |
| **India** | Fresh fish, eggs, fresh milk | DGLV | Sheep liver, carrot, DGLV, Pumpkin, chicken liver, fresh milk, eggs | DGLV |  | DGLV, legumes, okra, chicken liver | Sheep liver, chicken liver, fresh fish, fresh milk, eggs |
| **Pakistan** | Fresh fish, chicken, eggs, beef | DGLV | Carrot, beef liver, DGLV, chicken liver, eggs, fresh milk | DGLV |  | DGLV, chicken liver, legumes, okra, beef liver | Beef liver, chicken liver, fresh fish, fresh milk, eggs, beef, yogurt |
| **Unaffordable (at a threshold of greater than 50% of total adjusted household food expenditure)** | | | | | | | |
| **Bangladesh** |  | Beef | Fresh fish |  | Chicken, chicken liver |  |  |
| **India** |  | Goat/mutton |  |  | Chicken |  |  |
| **Pakistan** |  | Beef |  |  |  |  |  |

Note: Table shows the foods for each nutrient and country combination that cost less than 5% and 10% and greater than 50% of total household food expenditure per adult equivalent on average. Foods are ordered from least to most expensive. A blank cell indicates that no foods were affordable (or unaffordable) under a given threshold. “DGLV” = Dark Green Leafy Vegetables.

# Figure S1: Share of micronutrient requirements across portion sizes (plant-source foods)


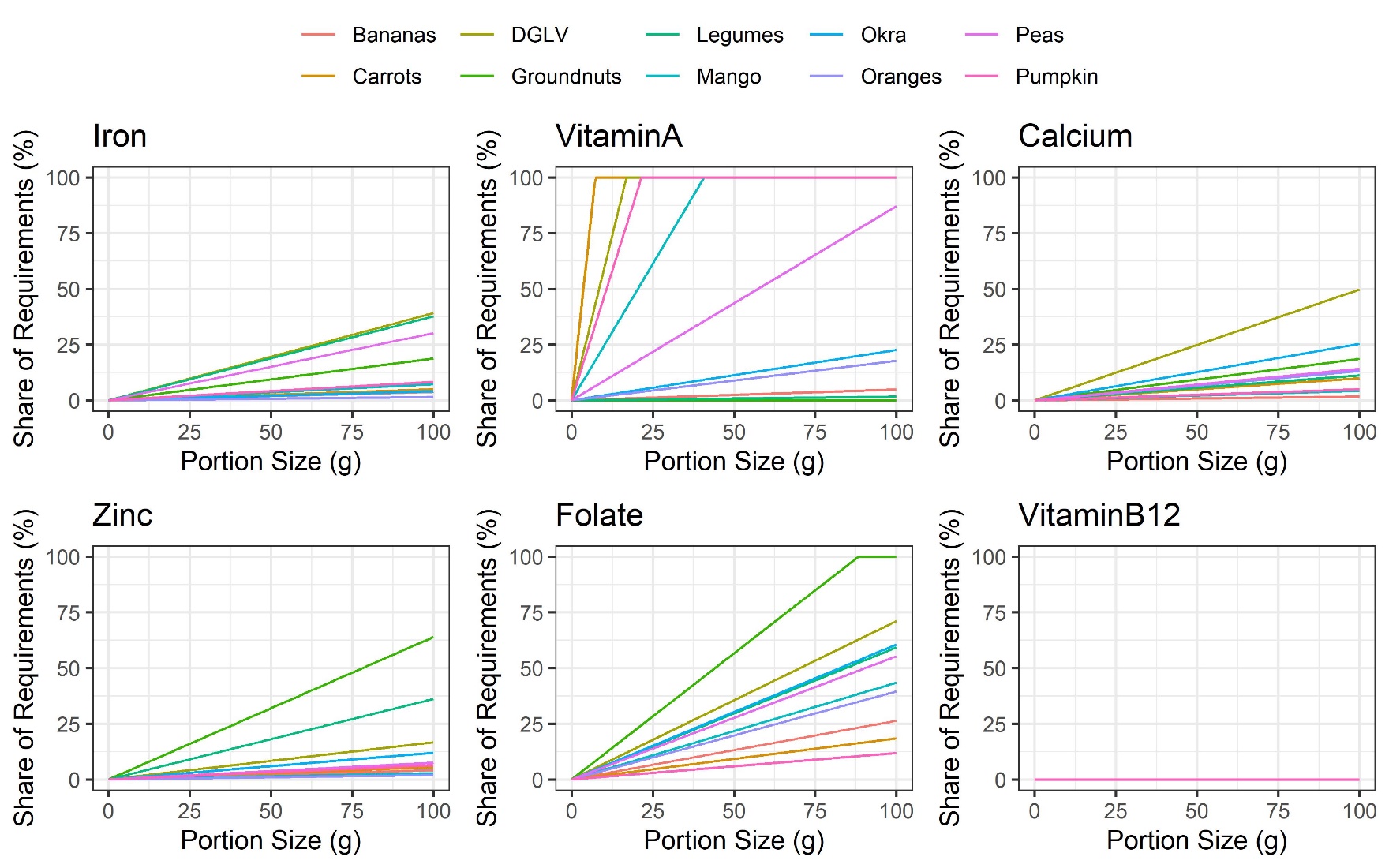


# Figure S2: Share of micronutrient requirements across portion sizes (animal-source foods)


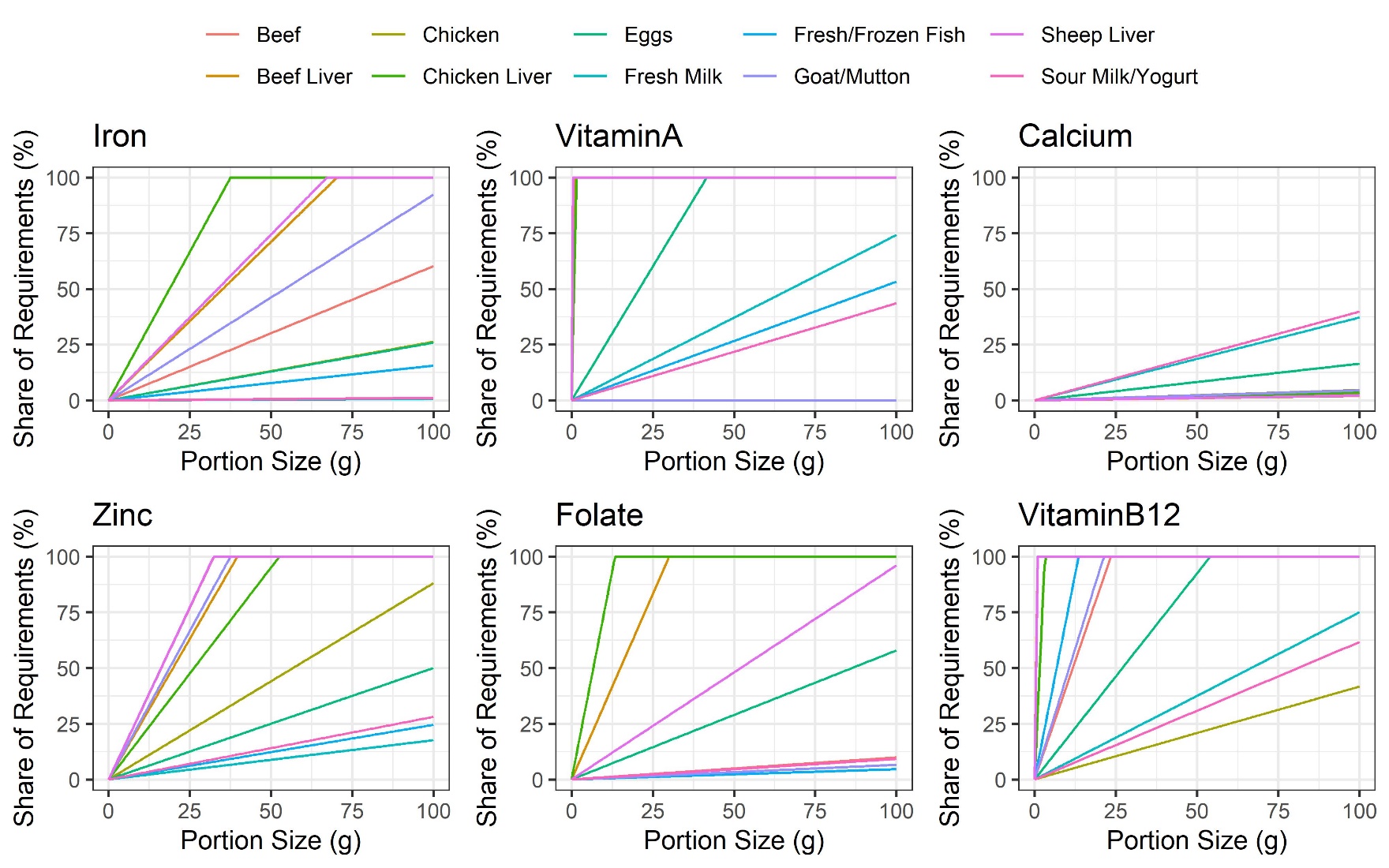


# Figure S3: Average share of micronutrient requirements across portion sizes, by food


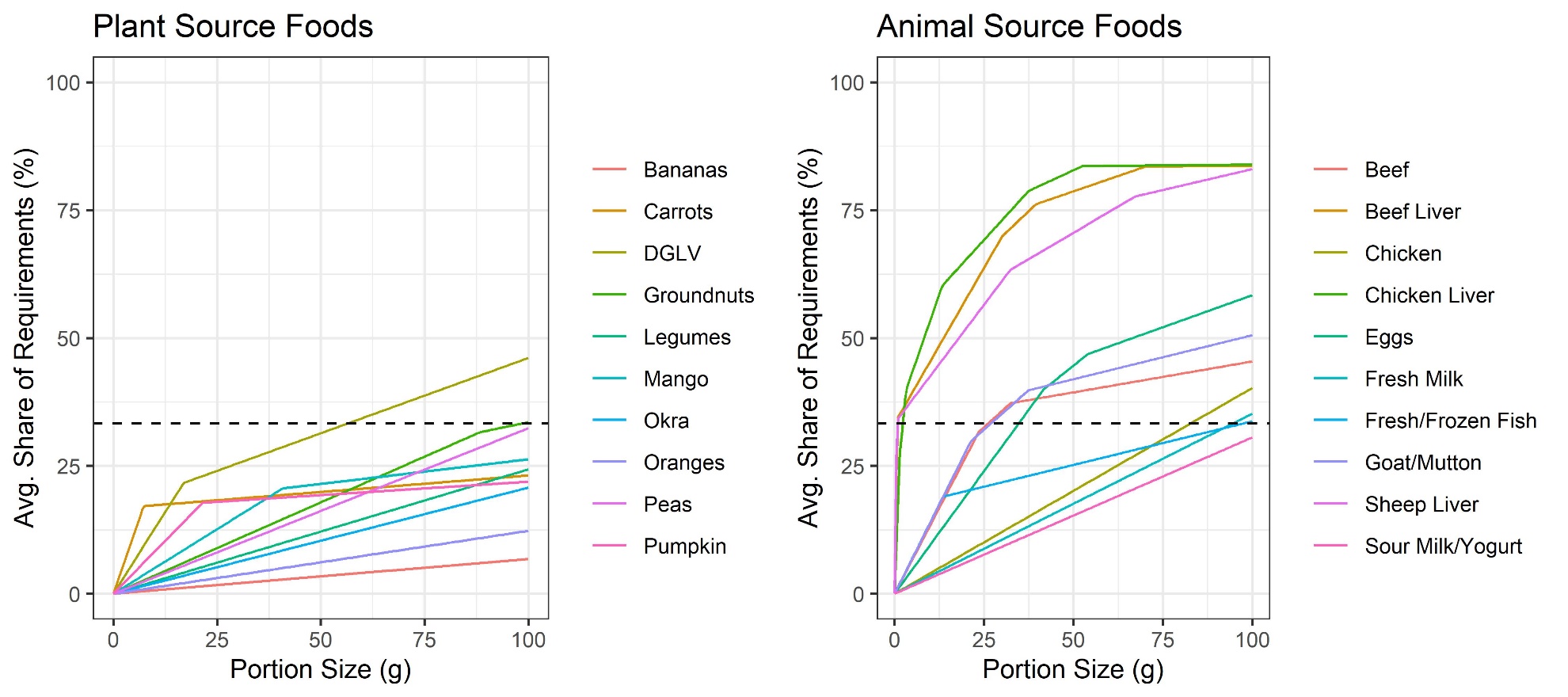


# Figure S4: Total household food and non-food expenditure, by rural/urban setting

*
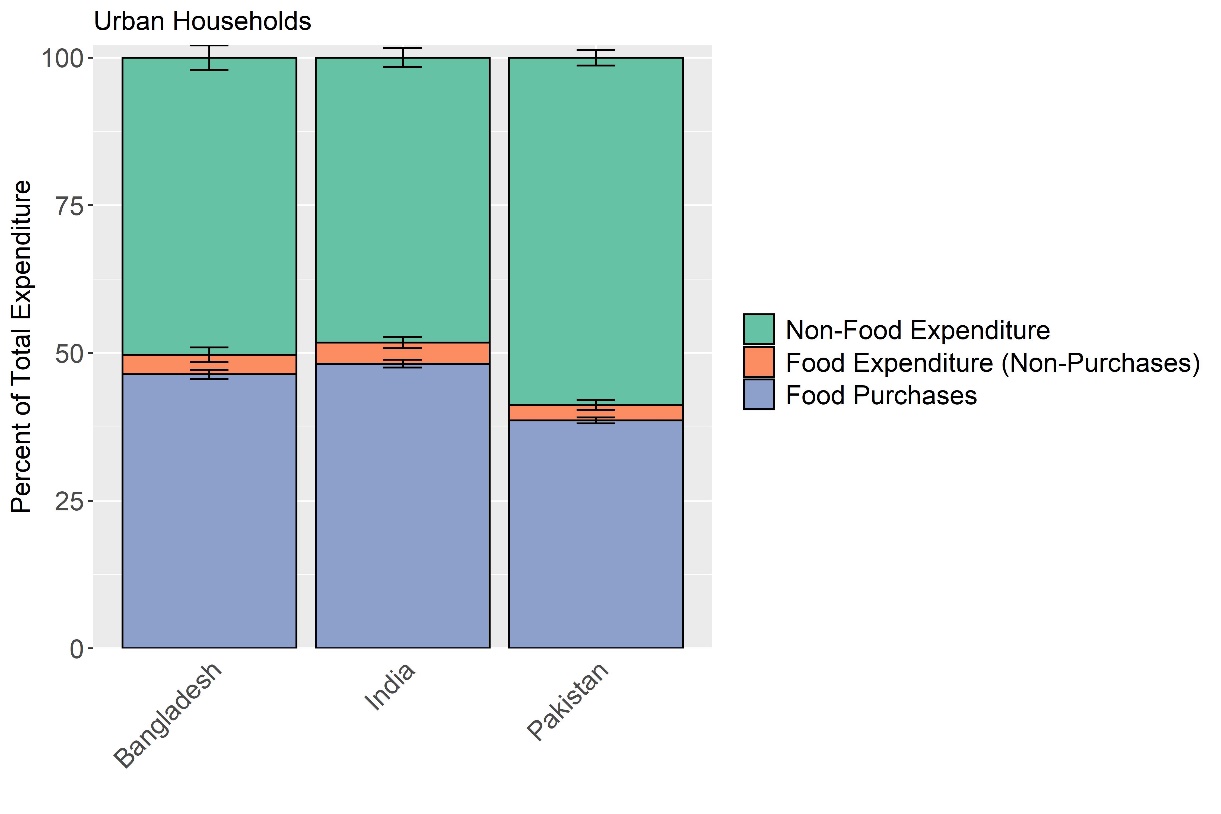

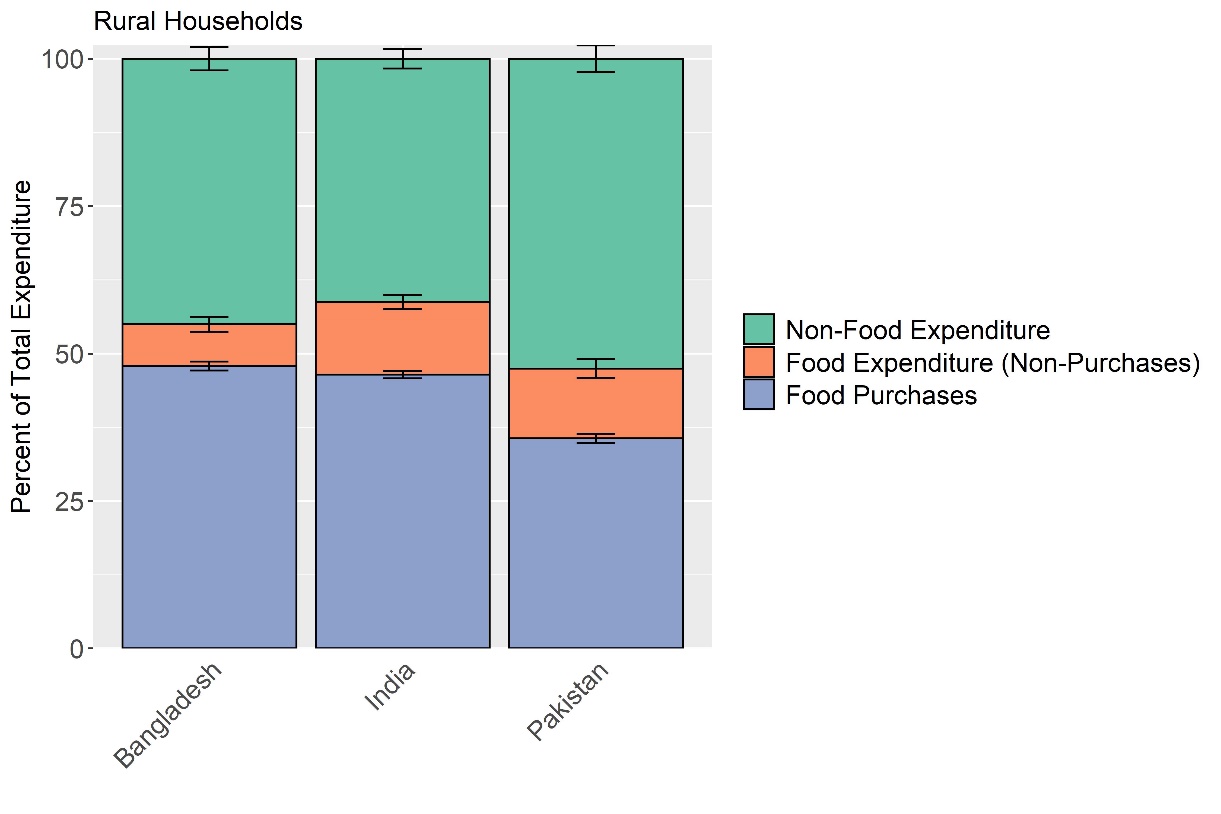
*

Note: only households with children of complementary feeding age are shown. Error bars represent 95% confidence intervals. Different surveys recorded household consumption over different periods: Bangladesh (2 weeks), India (1 week for fruits, vegetables, roots and tubers, and meats; 30 days for cereal products, legumes, and dairy products), Pakistan (2 weeks for most foods, 1 month for cereals and legumes), but we converted expenditures to weekly values in our analysis.

Figure S5: Total household food and non-food expenditure, by quintile


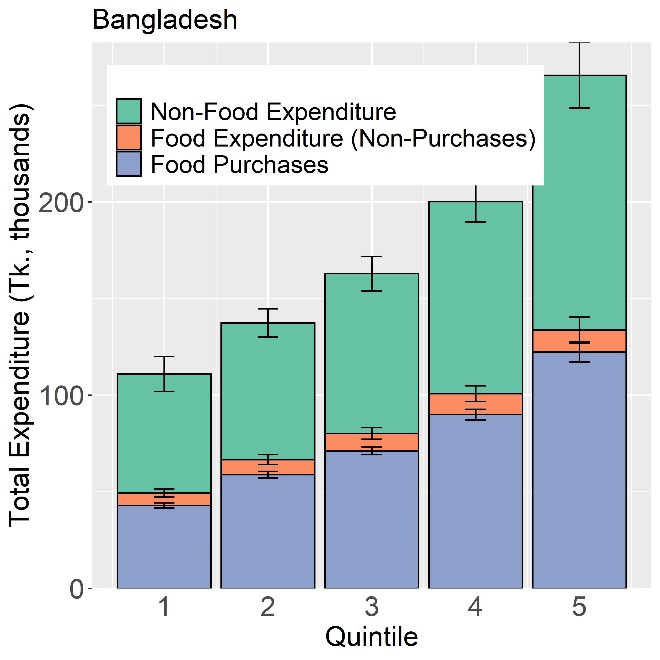

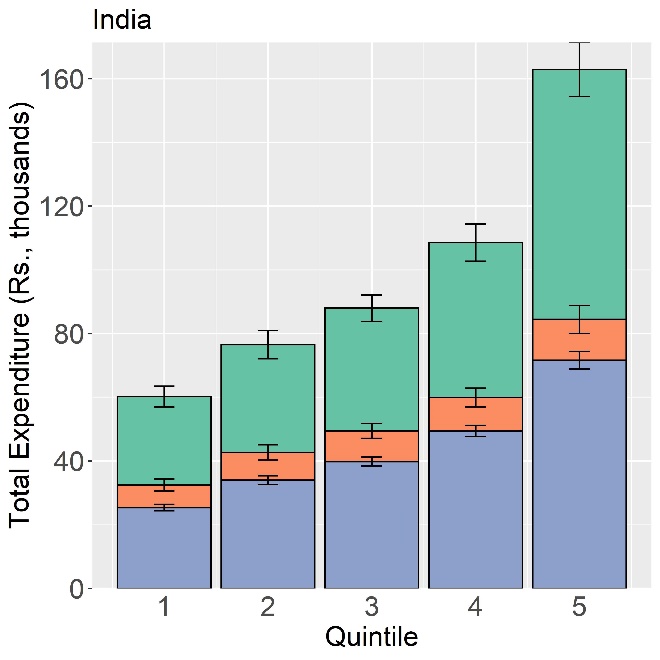

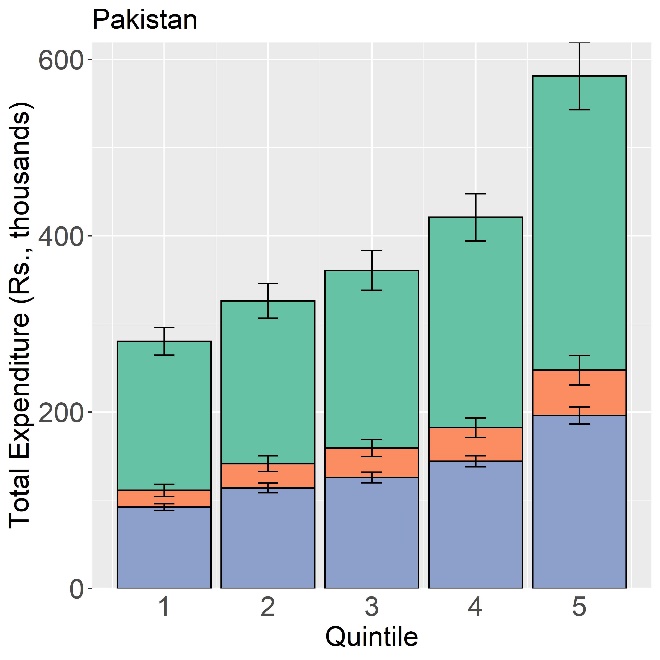


Note: only households with children of complementary feeding age are shown. Error bars represent 95% confidence intervals. Different surveys recorded household consumption over different periods: Bangladesh (2 weeks), India (1 week for fruits, vegetables, roots and tubers, and meats; 30 days for cereal products, legumes, and dairy products), Pakistan (2 weeks for most foods, 1 month for cereals and legumes), but we converted expenditures to weekly values in our analysis. Quintiles are based on household food expenditures per AEQ.

# Figure S6: Household consumption of key food groups, by rural/urban setting


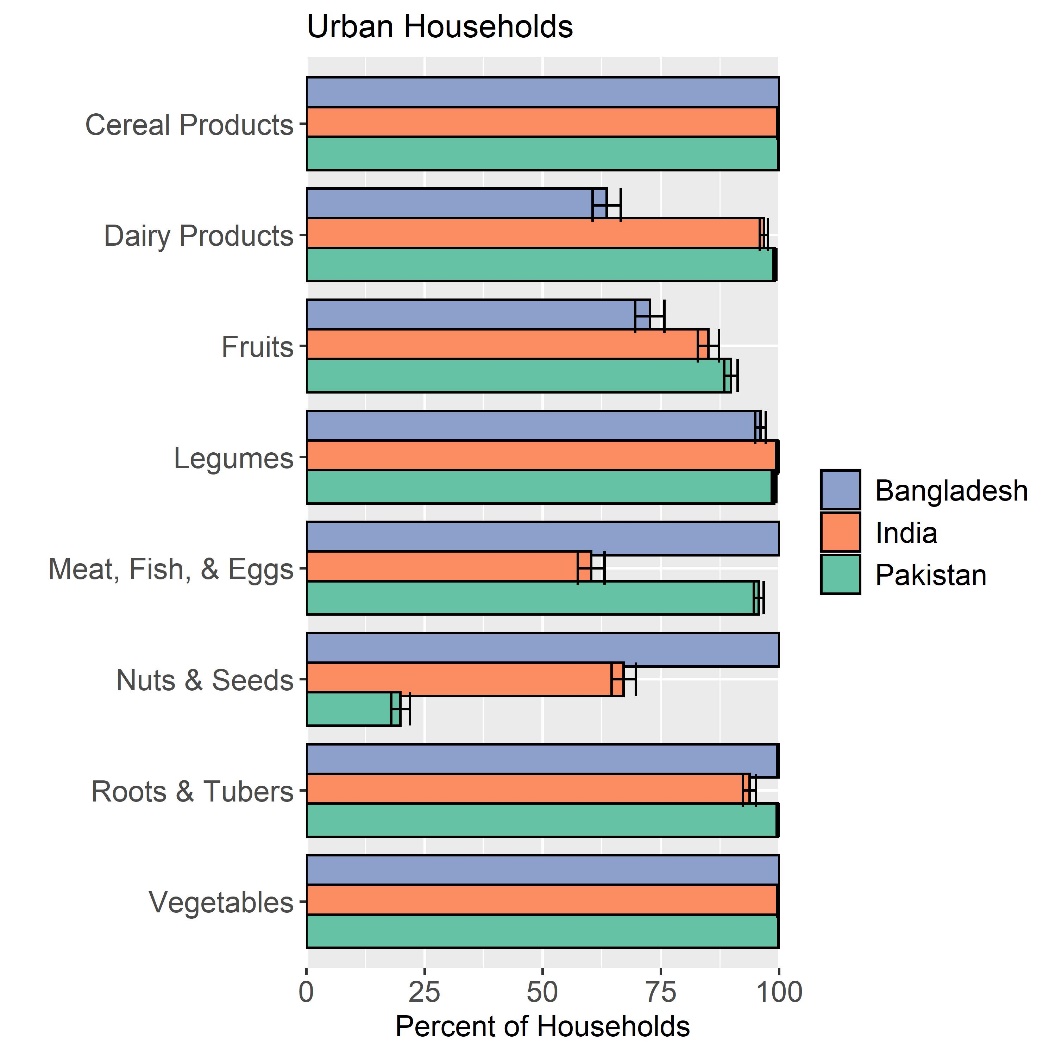

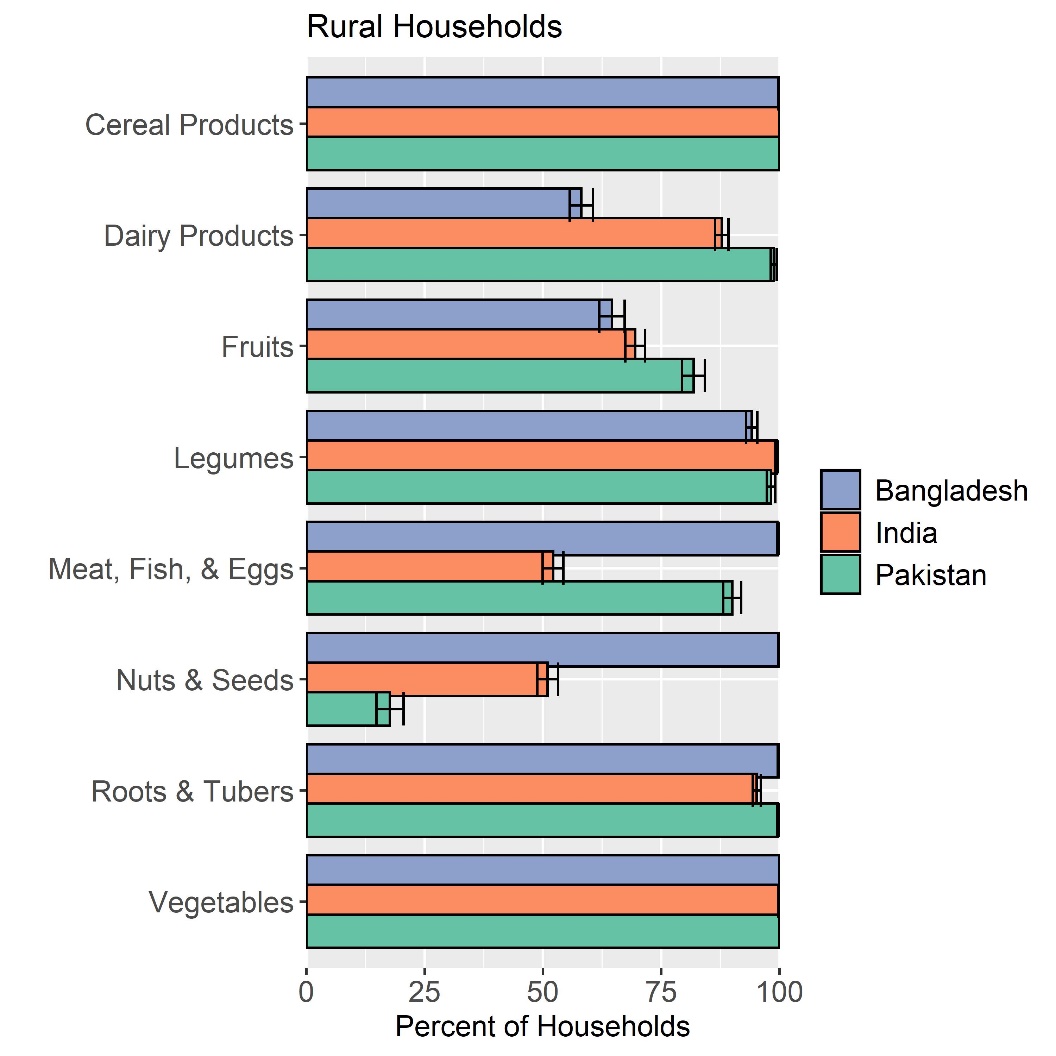


Note: only households with children of complementary feeding age are shown. Error bars represent 95% confidence intervals. Different surveys recorded household consumption over different periods: Bangladesh (2 weeks), India (1 week for fruits, vegetables, roots and tubers, and meats; 30 days for cereal products, legumes, and dairy products), Pakistan (2 weeks for most foods, 1 month for cereals and legumes).

# Figure S7: Weekly household expenditure by food group and rural/urban setting


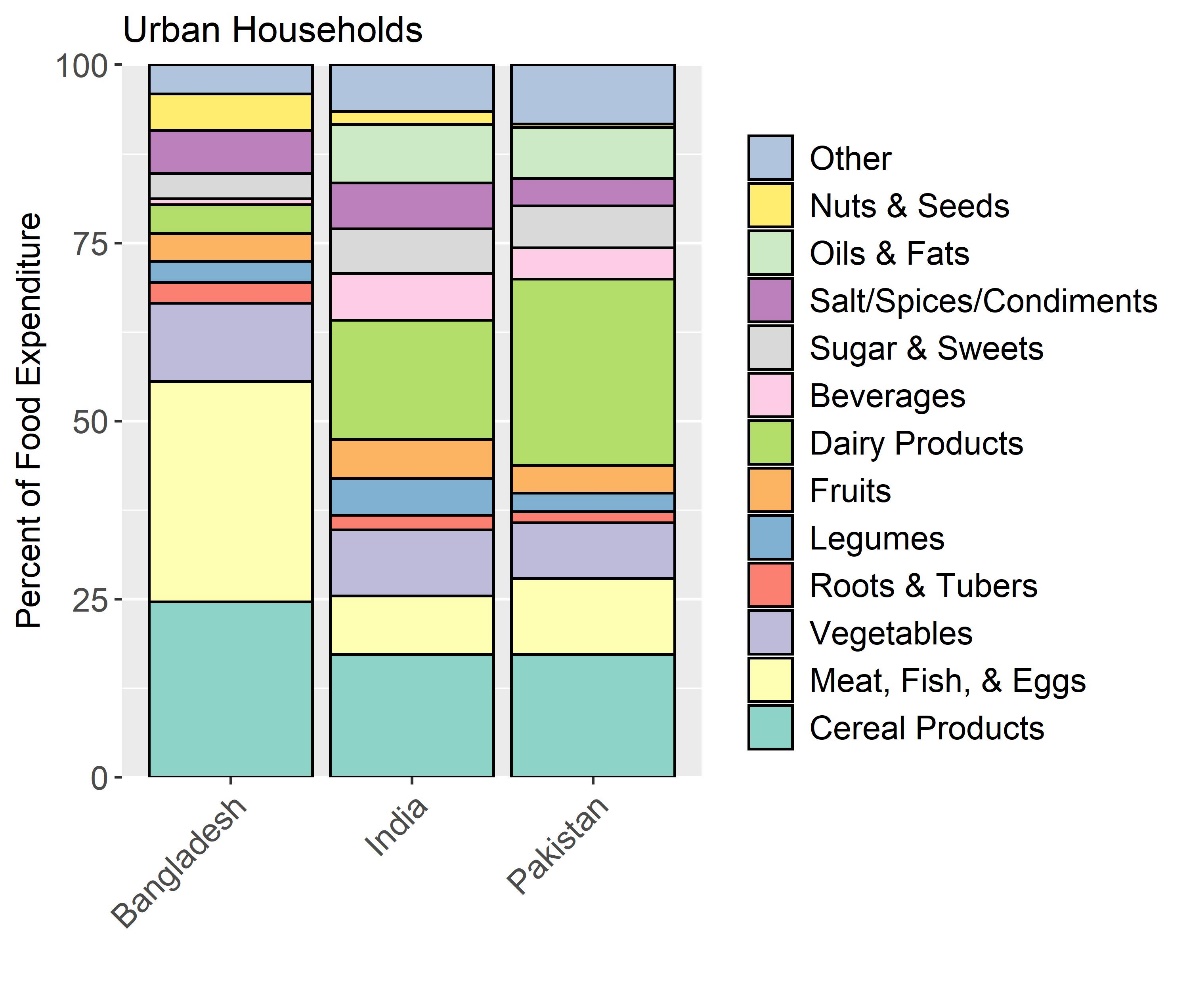

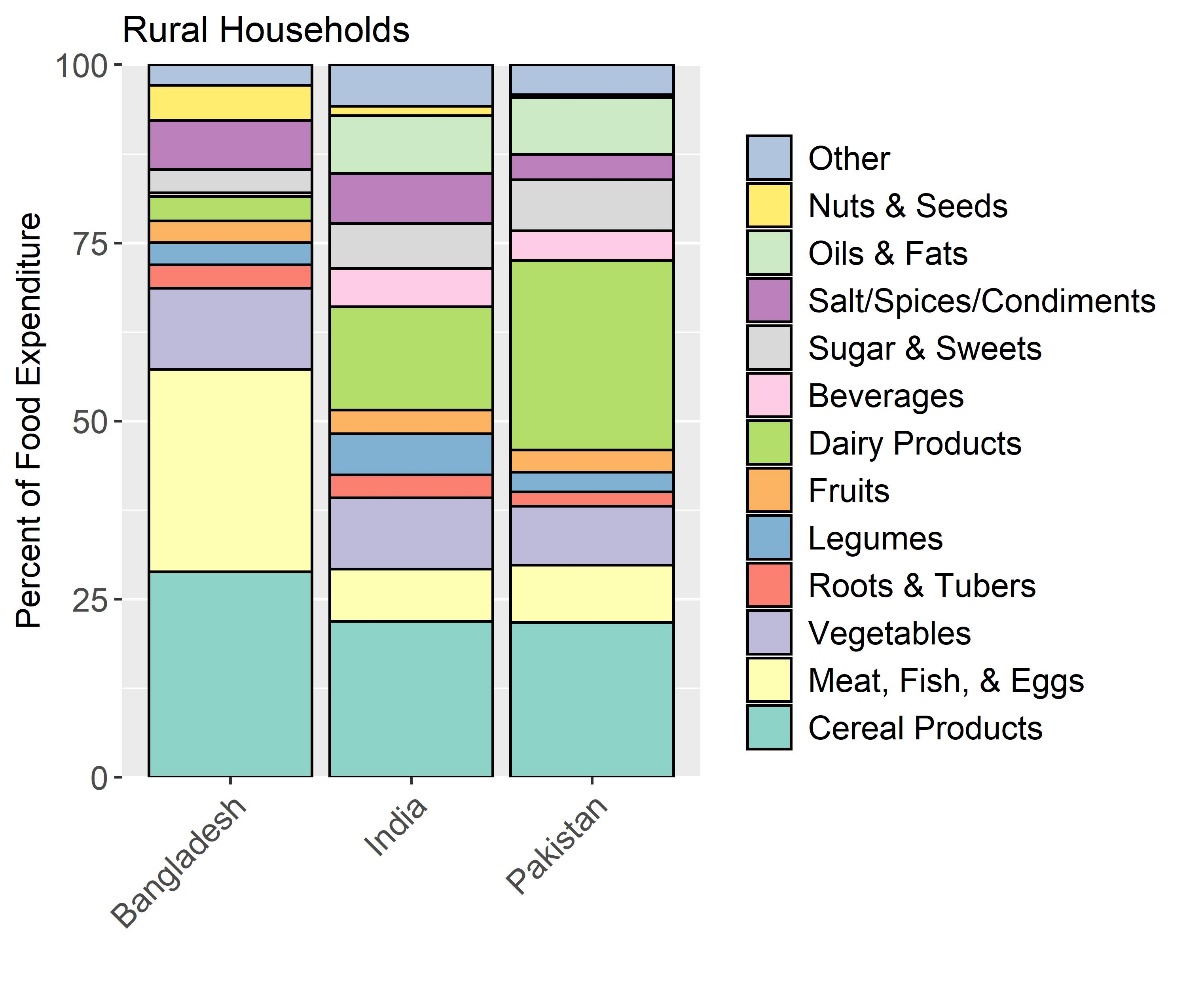


Note: only households with children of complementary feeding age are shown. Expenditure includes value of consumption from all sources (purchases, own-production, and in-kind). Different surveys recorded household consumption over different periods: Bangladesh (2 weeks), India (1 week for fruits, vegetables, roots and tubers, and meats; 30 days for cereal products, legumes, and dairy products), Pakistan (2 weeks for most foods, 1 month for cereals and legumes), but we converted expenditures to weekly values in our analysis.

# Figure S8: Household consumption of key food groups, by quintile

**
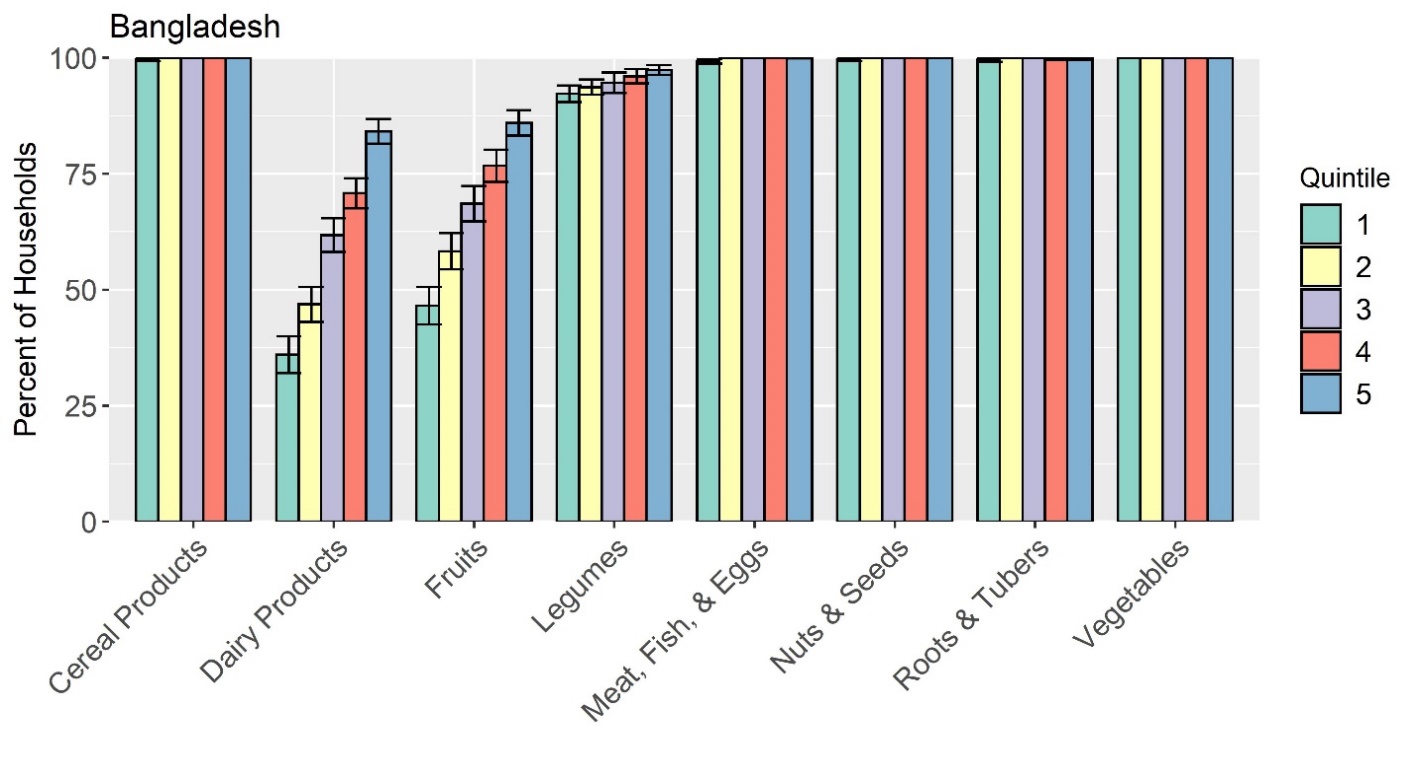

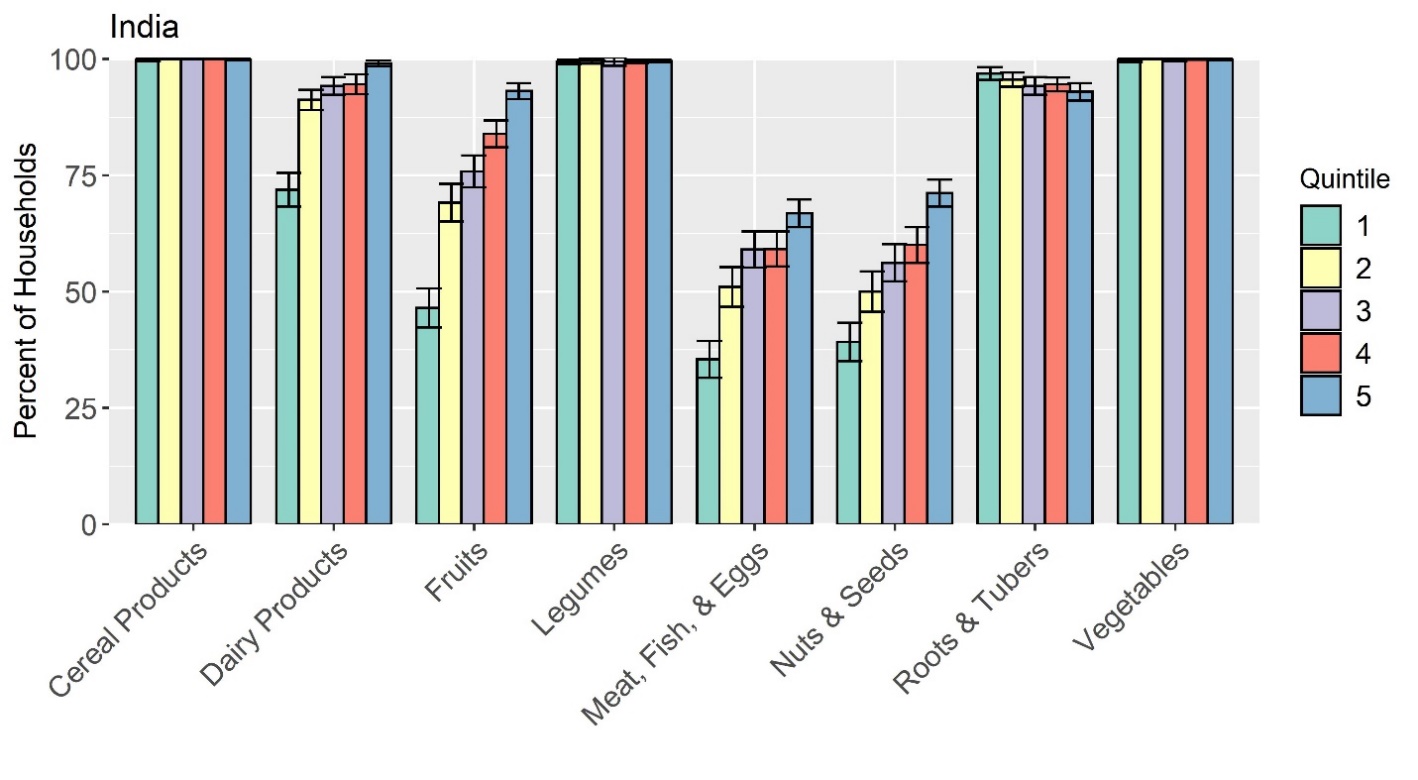

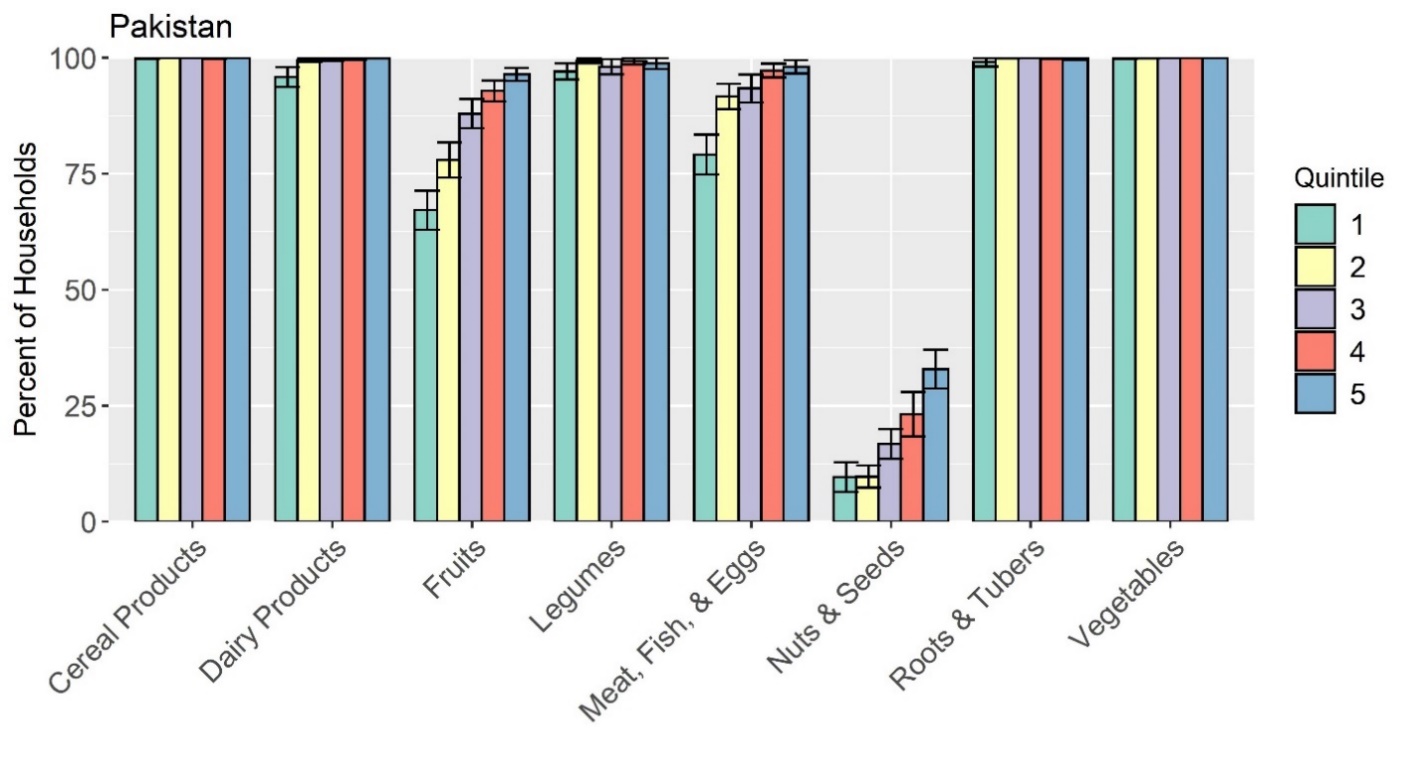
**

Note: only households with children of complementary feeding age are shown. Error bars represent 95% confidence intervals. Different surveys recorded household consumption over different periods: Bangladesh (2 weeks), India (1 week for fruits, vegetables, roots and tubers, and meats; 30 days for cereal products, legumes, and dairy products), Pakistan (2 weeks for most foods, 1 month for cereals and legumes). Quintiles are based on household food expenditures per AEQ.

# Figure S9: Proportional weekly household expenditure by food group and quintile


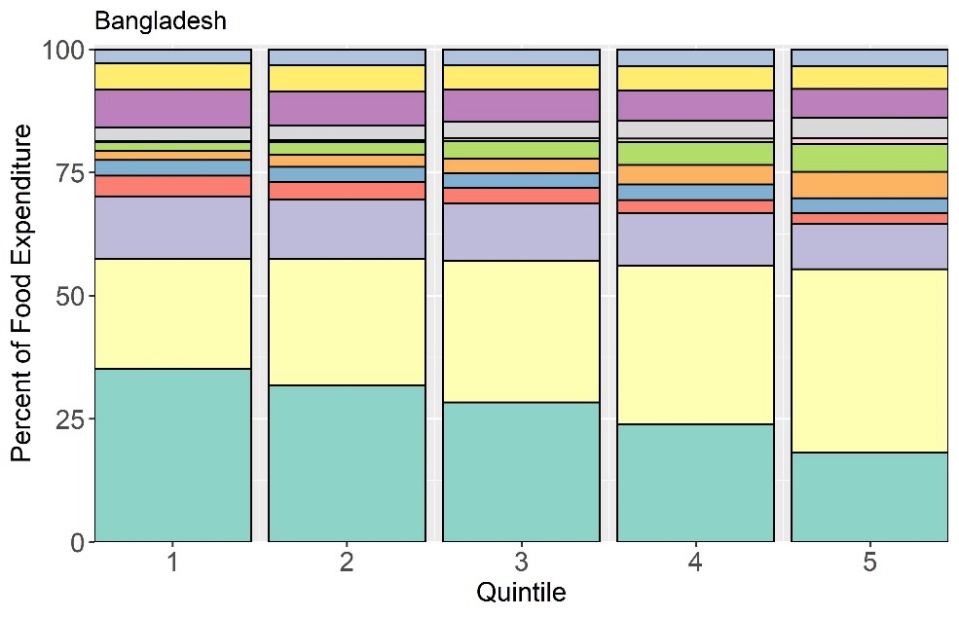

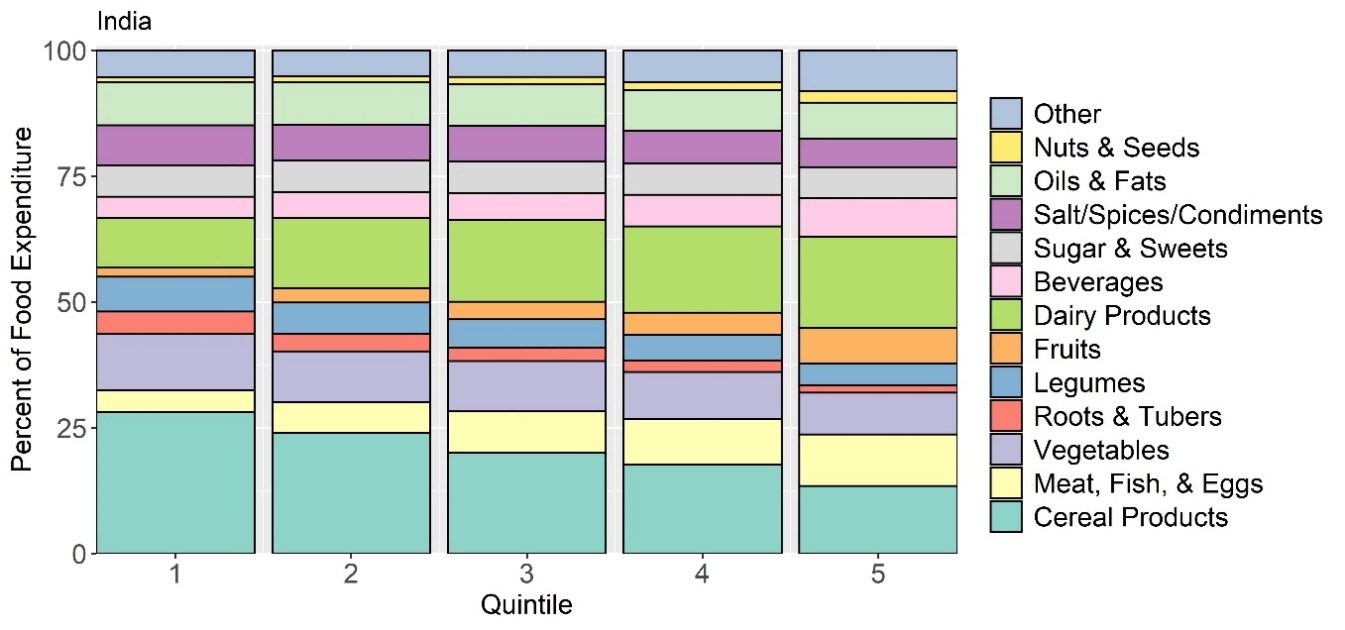

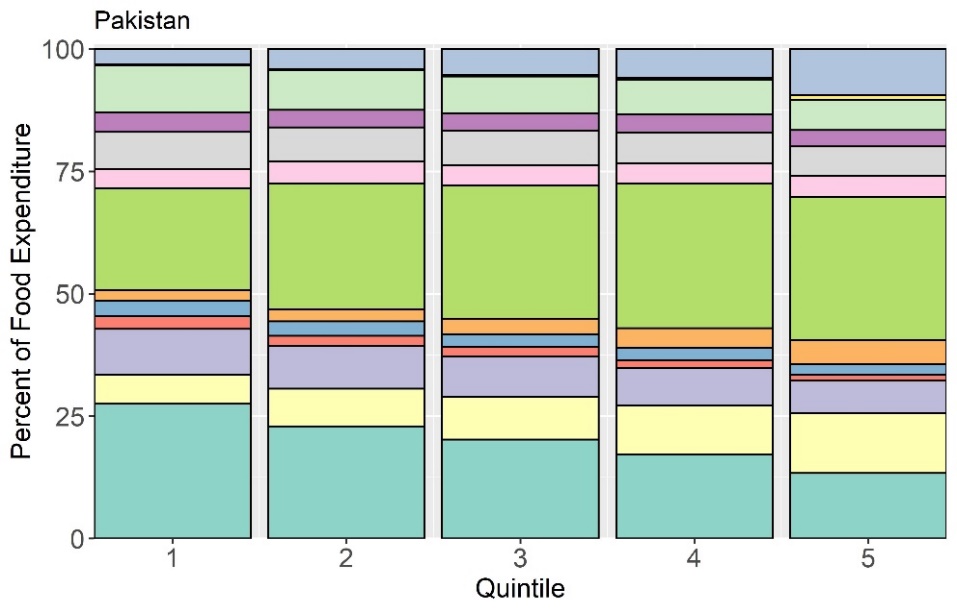


Note: only households with children of complementary feeding age are shown. Expenditure includes value of consumption from all sources (purchases, own-production, in-kind). Different surveys recorded household consumption over different periods: Bangladesh (2 weeks), India (1 week for fruits, vegetables, roots and tubers, and meats; 30 days for cereal products, legumes, and dairy products), Pakistan (2 weeks for most foods, 1 month for cereals and legumes), but we converted expenditures to weekly values in our analysis. Quintiles are based on household food expenditures per AEQ.

# Figure S10A: Total weekly household expenditure by food group and quintile (local currency)


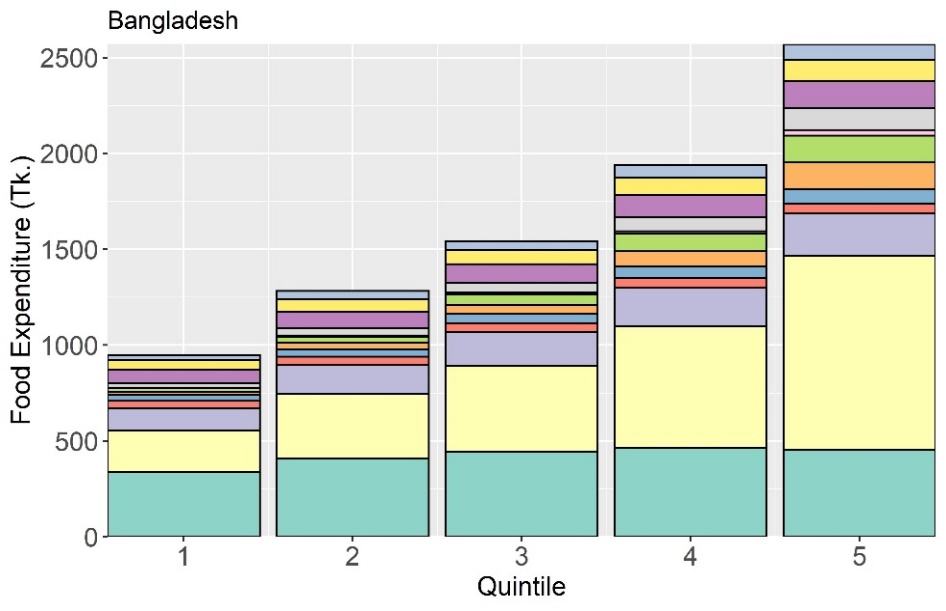

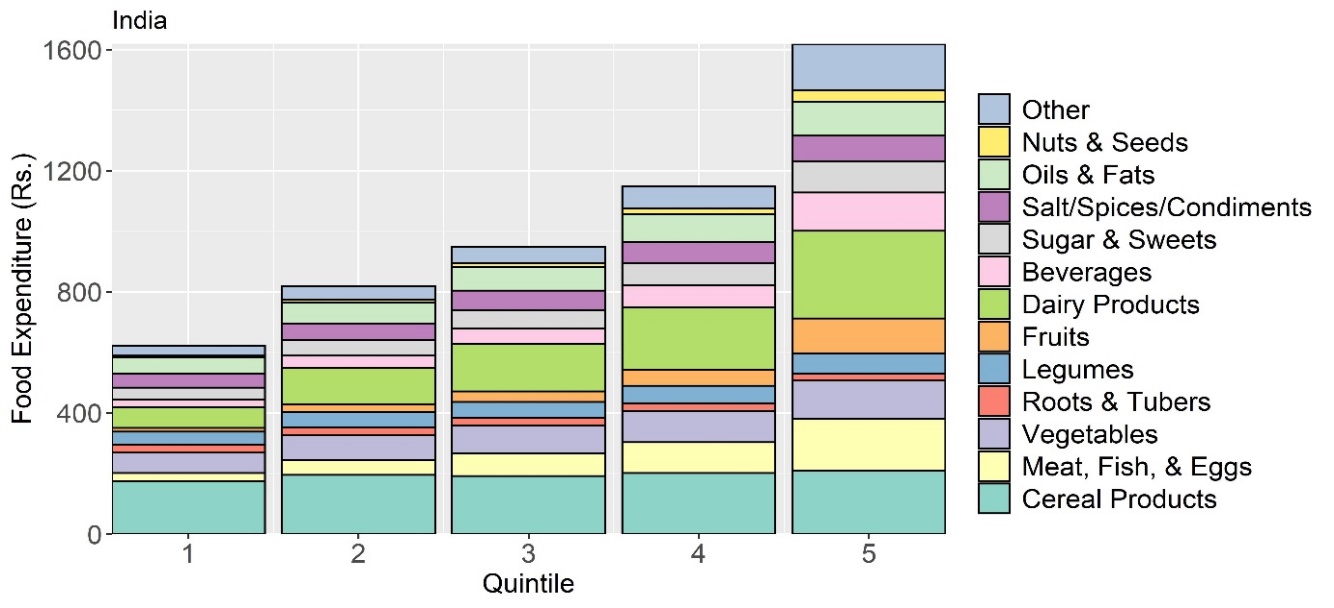


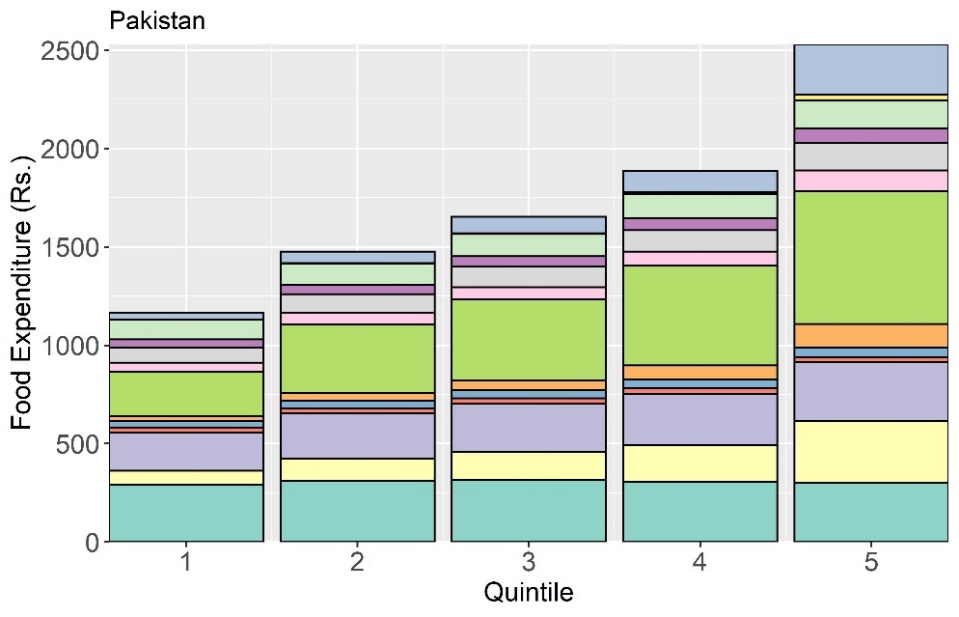


Note: only households with children of complementary feeding age are shown. Expenditure includes value of consumption from all sources (purchases, own-production, and in-kind). Different surveys recorded household consumption over different periods: Bangladesh (2 weeks), India (1 week for fruits, vegetables, roots and tubers, and meats; 30 days for cereal products, legumes, and dairy products), Pakistan (2 weeks for most foods, 1 month for cereals and legumes), but we converted expenditures to weekly values in our analysis. Quintiles are based on household food expenditures per AEQ.

# Figure S10B: Total weekly household expenditure by food group and quintile (international dollars)


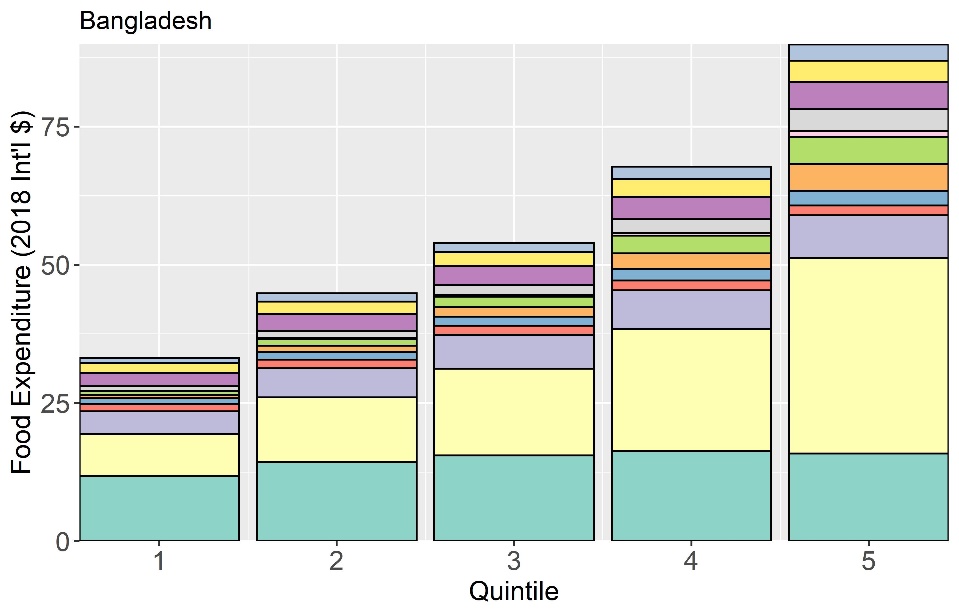

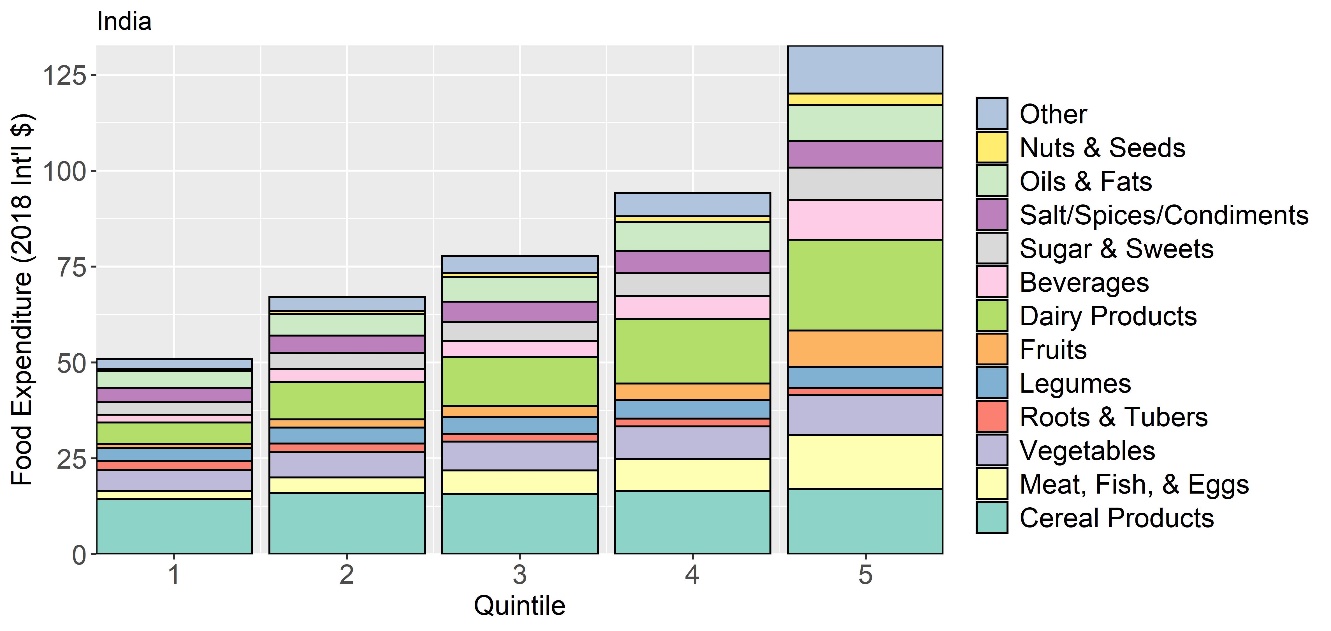

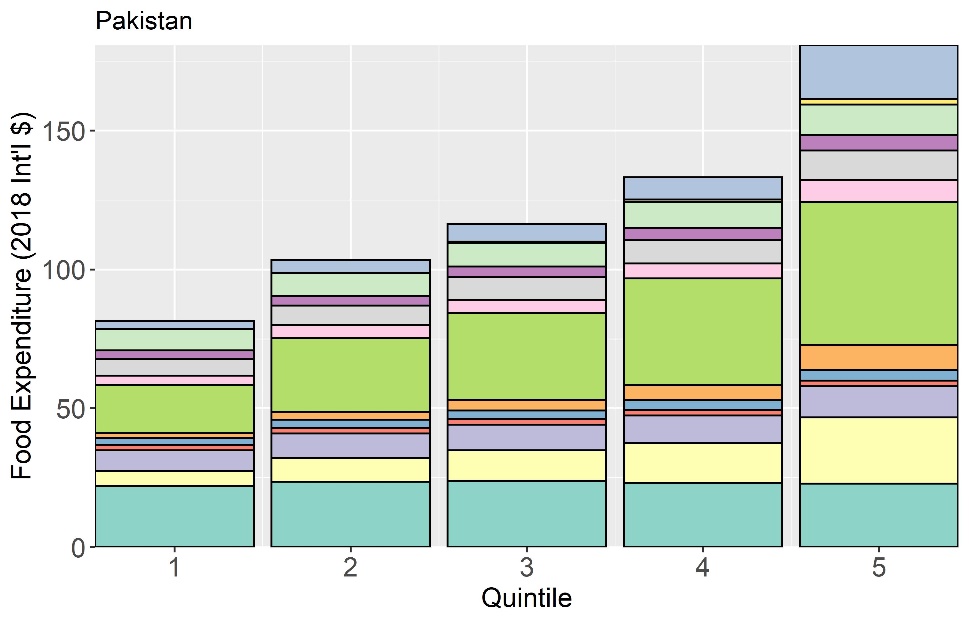


Note: only households with children of complementary feeding age are shown. Expenditure includes value of consumption from all sources (purchases, own-production, and in-kind). Different surveys recorded household consumption over different periods: Bangladesh (2 weeks), India (1 week for fruits, vegetables, roots and tubers, and meats; 30 days for cereal products, legumes, and dairy products), Pakistan (2 weeks for most foods, 1 month for cereals and legumes), but we converted expenditures to weekly values in our analysis. Quintiles are based on household food expenditures per AEQ.

# Figure S11: Current consumption of selected nutritious foods, by rural/urban setting


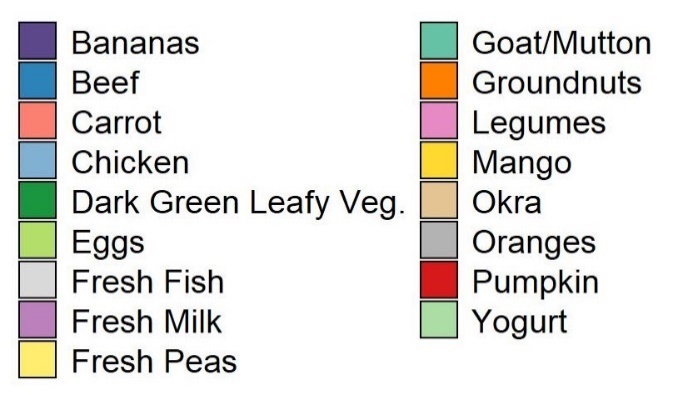

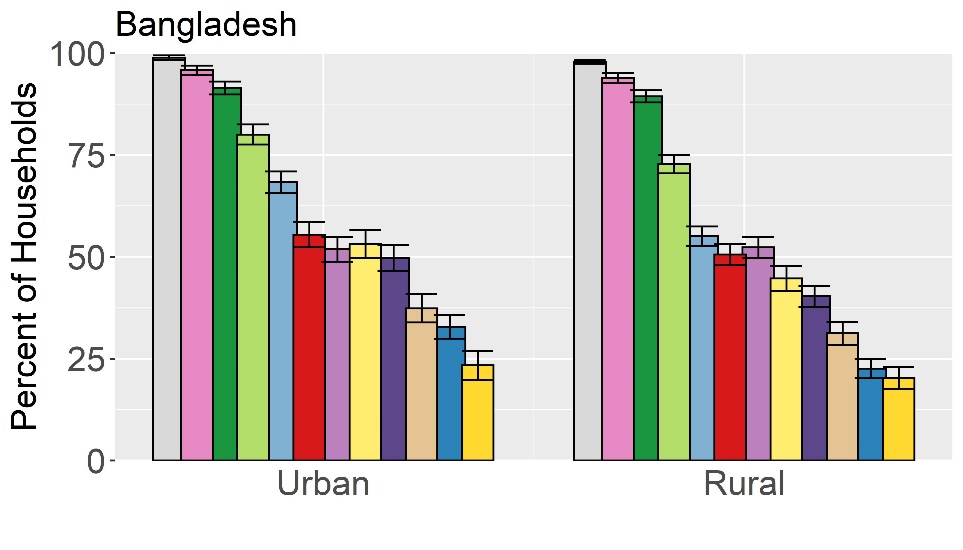

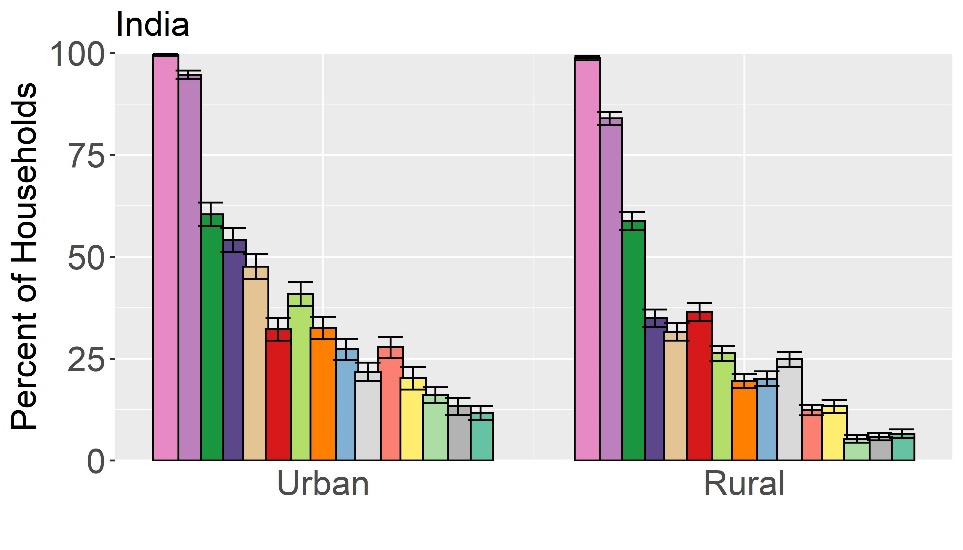

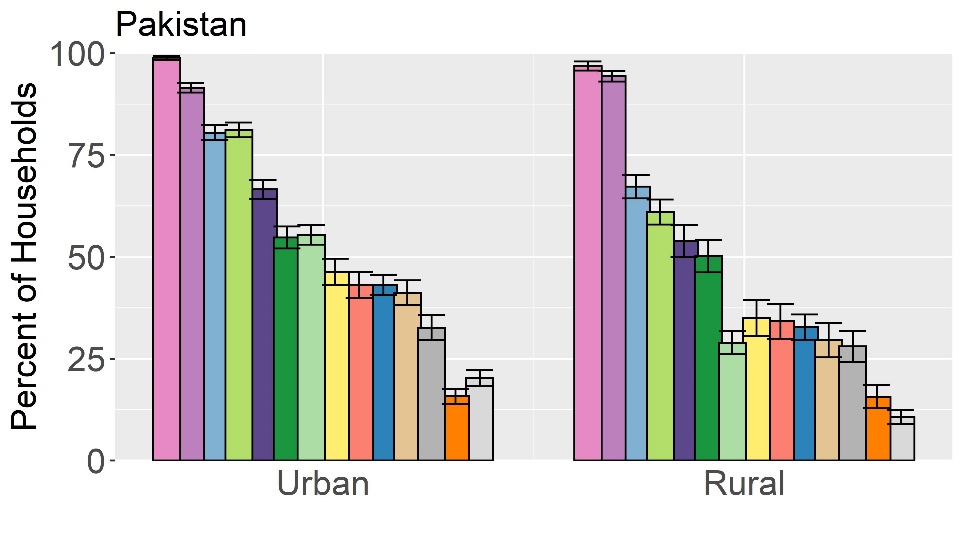


Note: only households with children of complementary feeding age are shown. Error bars represent 95% confidence intervals. Different surveys recorded household consumption over different periods: Bangladesh (2 weeks), India (1 week for fruits, vegetables, roots and tubers, and meats; 30 days for cereal products, legumes, and dairy products), Pakistan (2 weeks for most foods, 1 month for cereals and legumes).

# Figure S12: Current expenditure on selected nutritious foods, by rural/urban setting

**
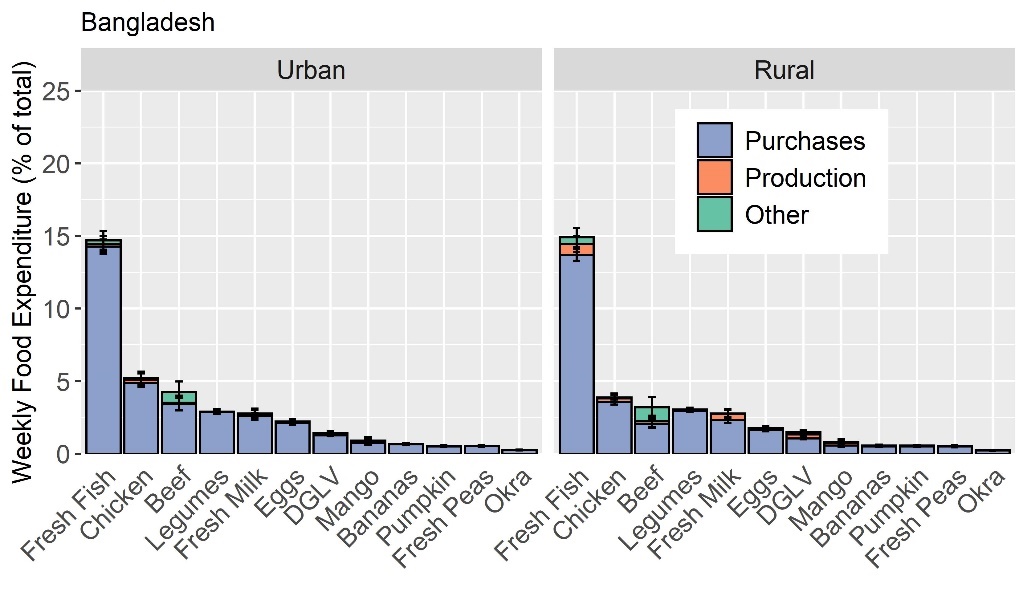

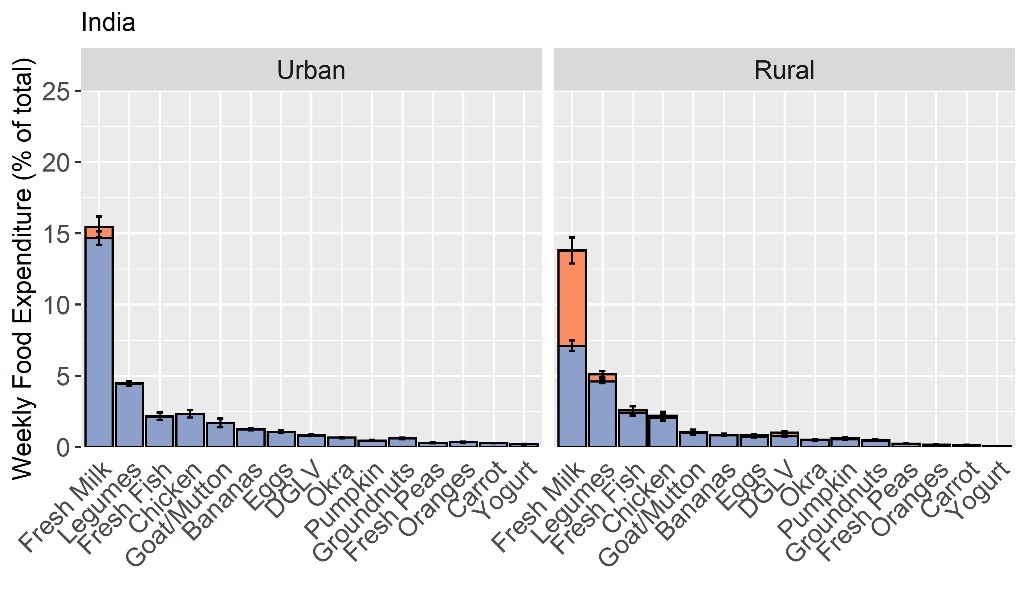

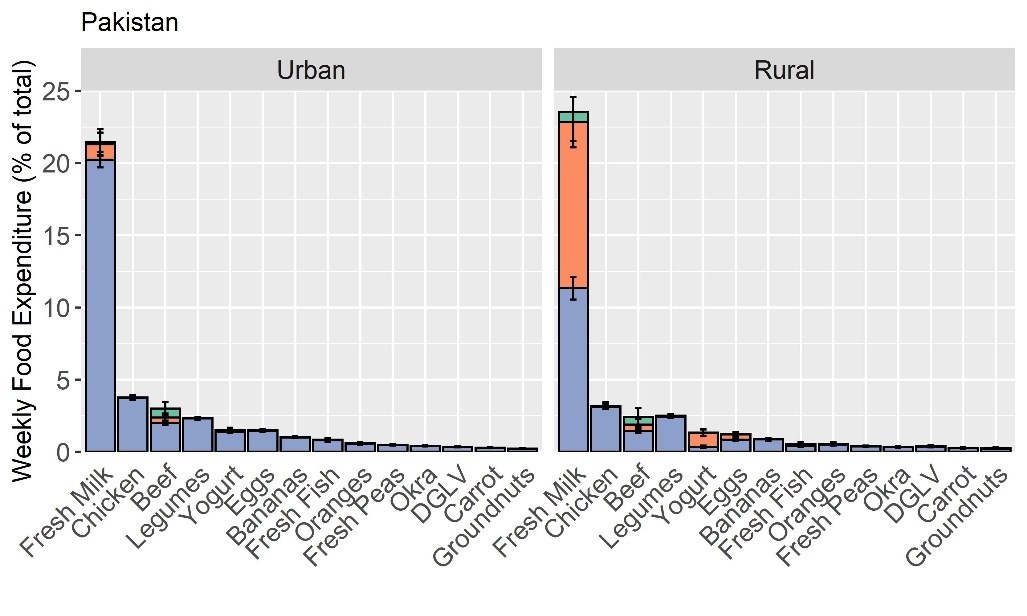
**

Note: only households with children of complementary feeding age are shown. Error bars represent 95% confidence intervals. Different surveys recorded household consumption over different periods: Bangladesh (2 weeks), India (1 week for fruits, vegetables, roots and tubers, and meats; 30 days for cereal products, legumes, and dairy products), Pakistan (2 weeks for most foods, 1 month for cereals and legumes), but we converted expenditures to weekly values in our analysis.


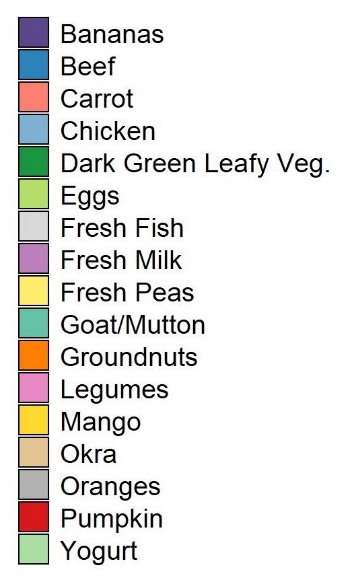
Figure S13: Current consumption of selected nutritious foods, by quintile
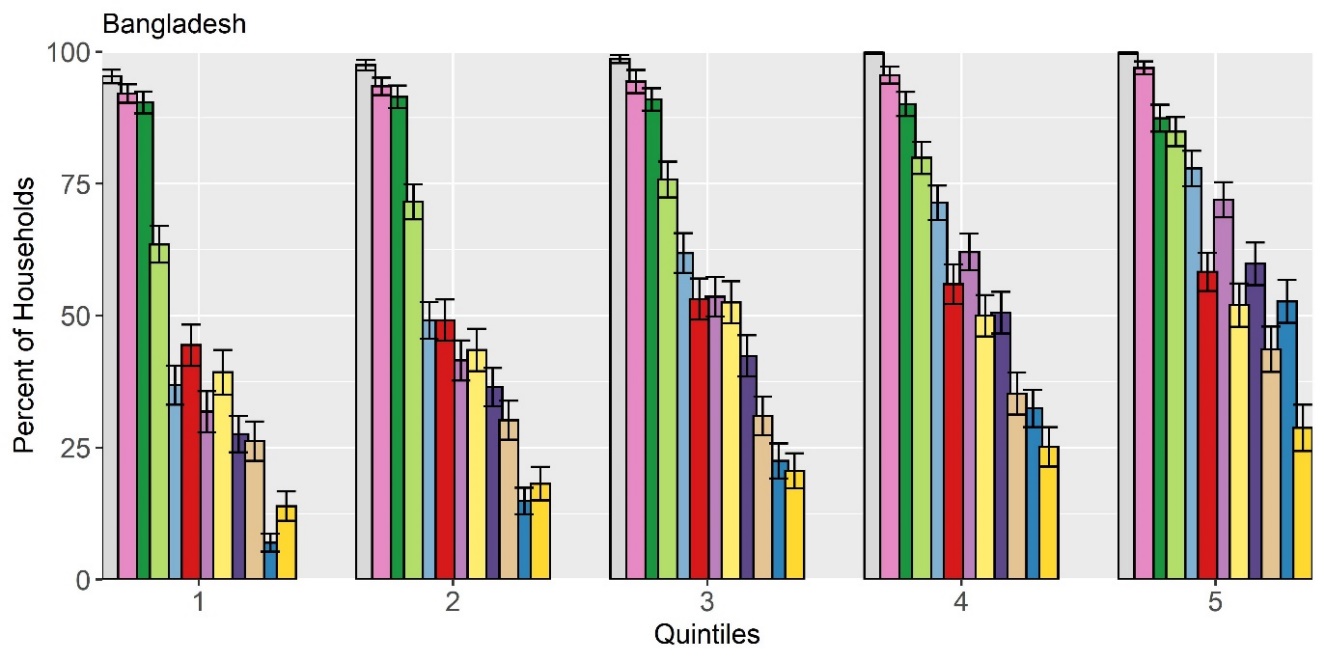

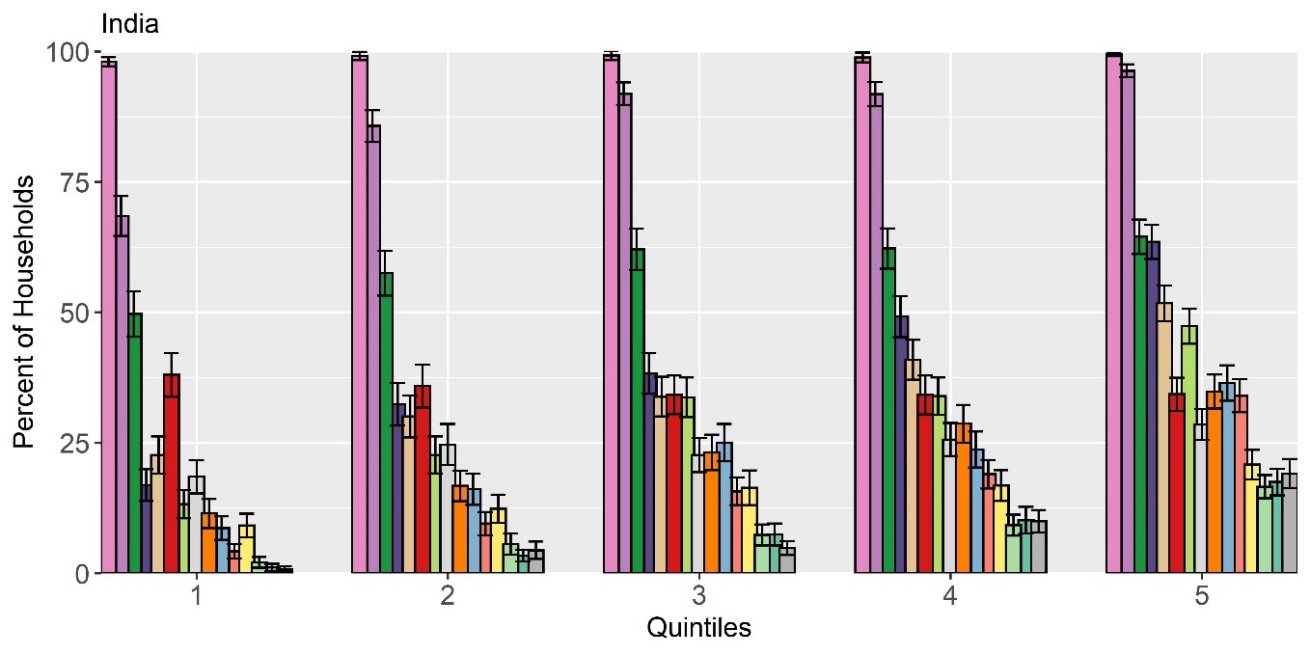


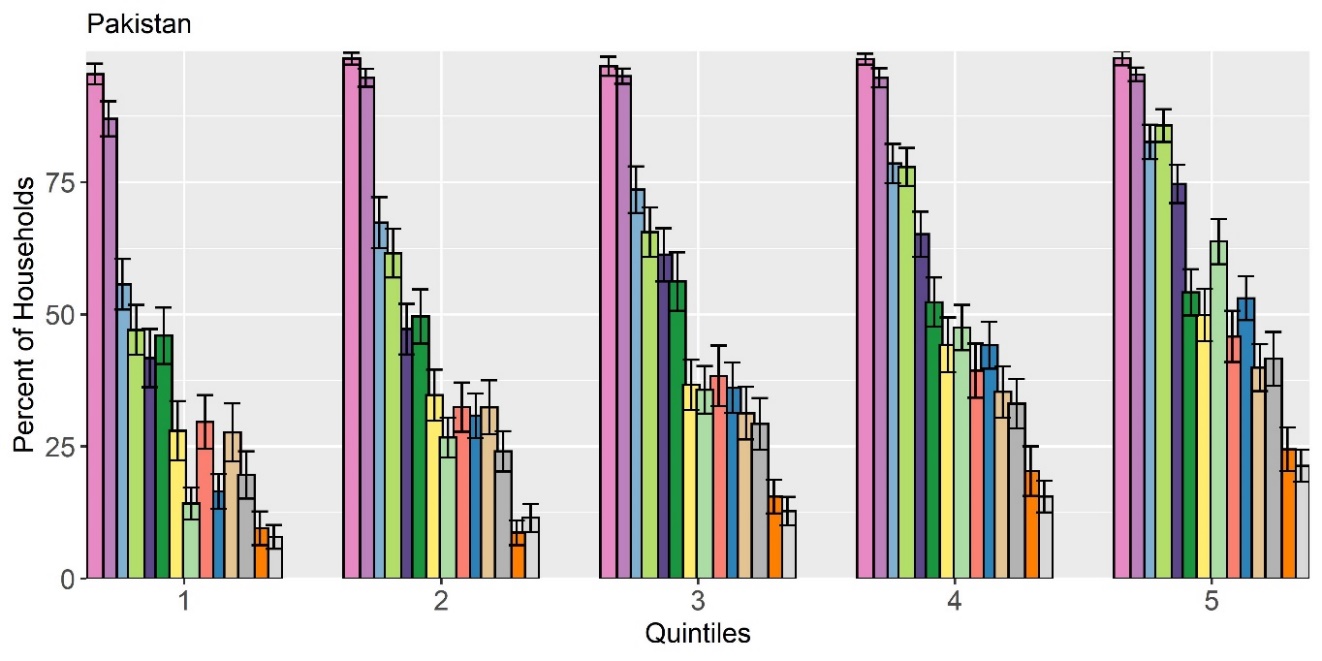


Note: only households with children of complementary feeding age are shown. Error bars represent 95 confidence intervals. Surveys recorded household consumption over different periods: Bangladesh (2 weeks), India (1 week for fruits, vegetables, roots and tubers, and meats; 30 days for cereal products, legumes, and dairy products), Pakistan (2 weeks for most foods, 1 month for cereals and legumes), but we converted expenditures to weekly values in our analysis. Quintiles are based on household food expenditures per AEQ.

# Figure S14: Current expenditure on selected nutritious foods, by quintile


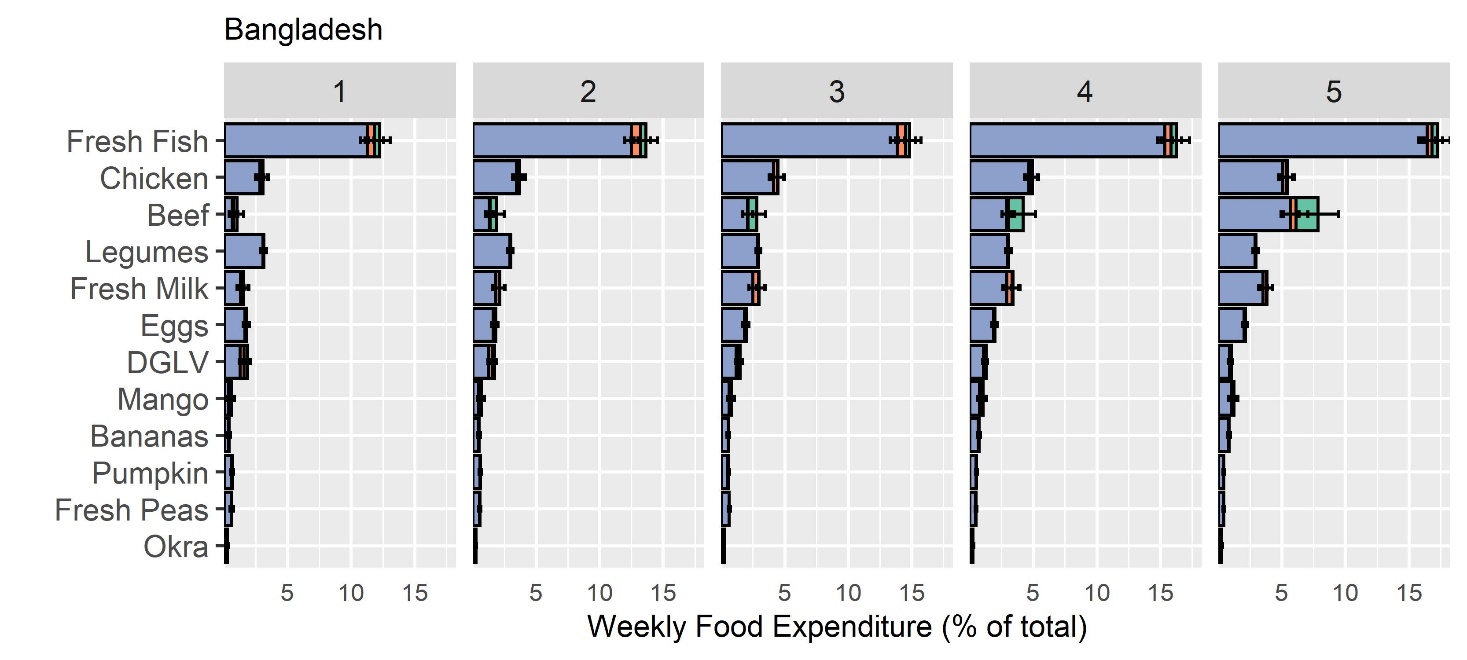

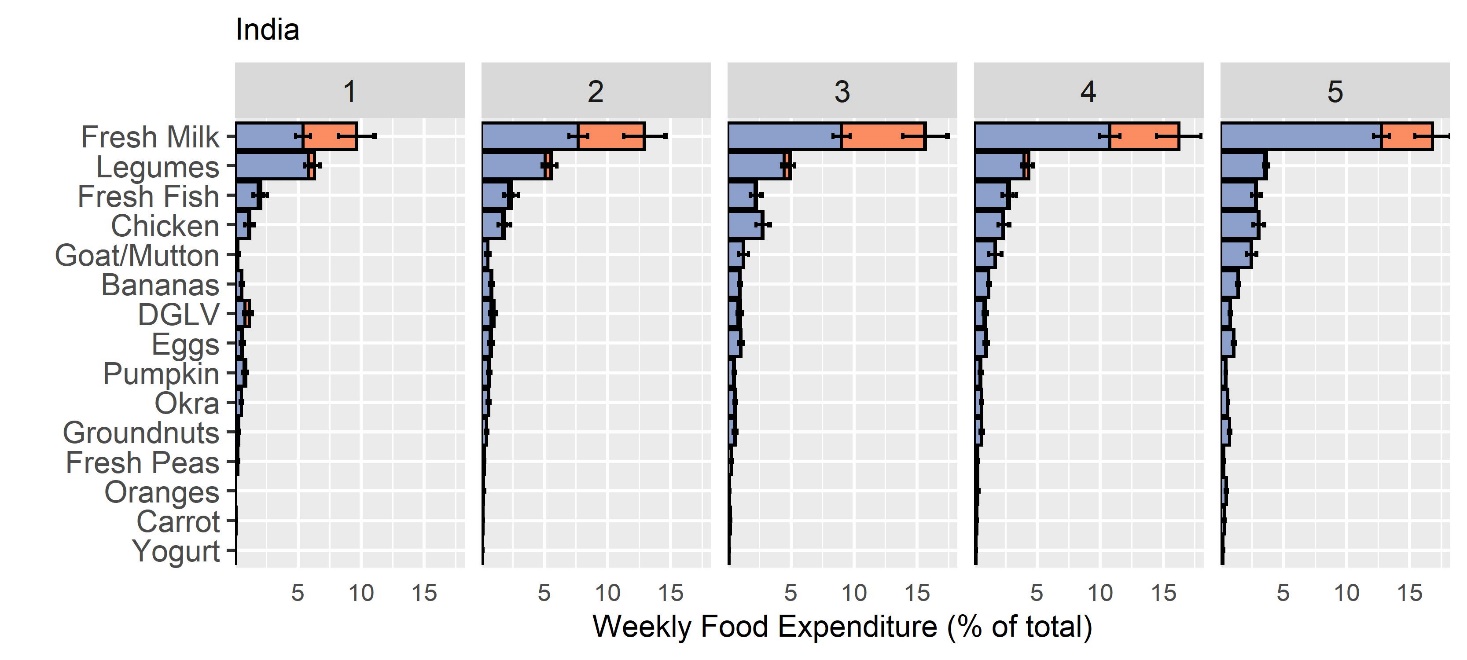

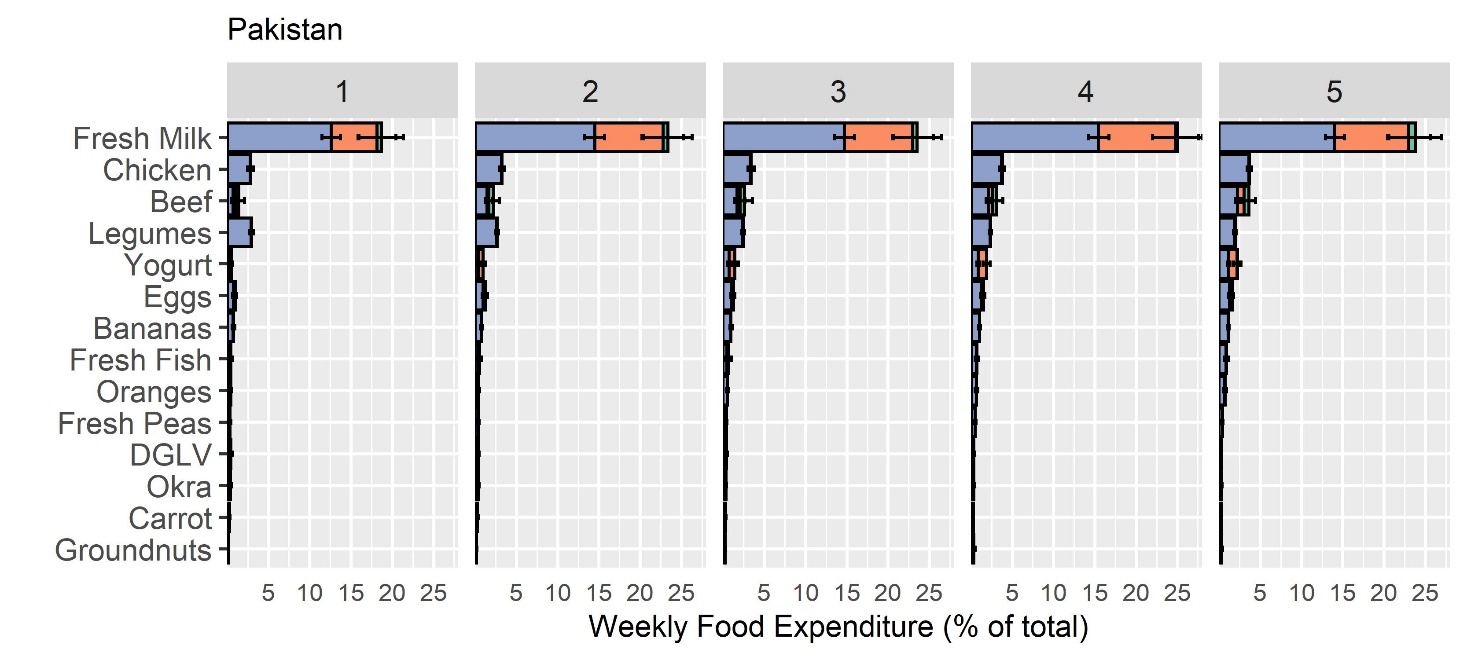


Note: only households with children of complementary feeding age are shown. Error bars represent 95 confidence intervals. Different surveys recorded household consumption over different periods: Bangladesh (2 weeks), India (1 week for fruits, vegetables, roots and tubers, and meats; 30 days for cereal products, legumes, and dairy products), Pakistan (2 weeks for most foods, 1 month for cereals and legumes), but we converted expenditures to weekly values in our analysis. Quintiles are based on household food expenditures per AEQ.

# Figure S15: Portion size cost, as a share of total household food expenditure per adult equivalent, by food


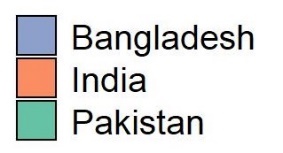

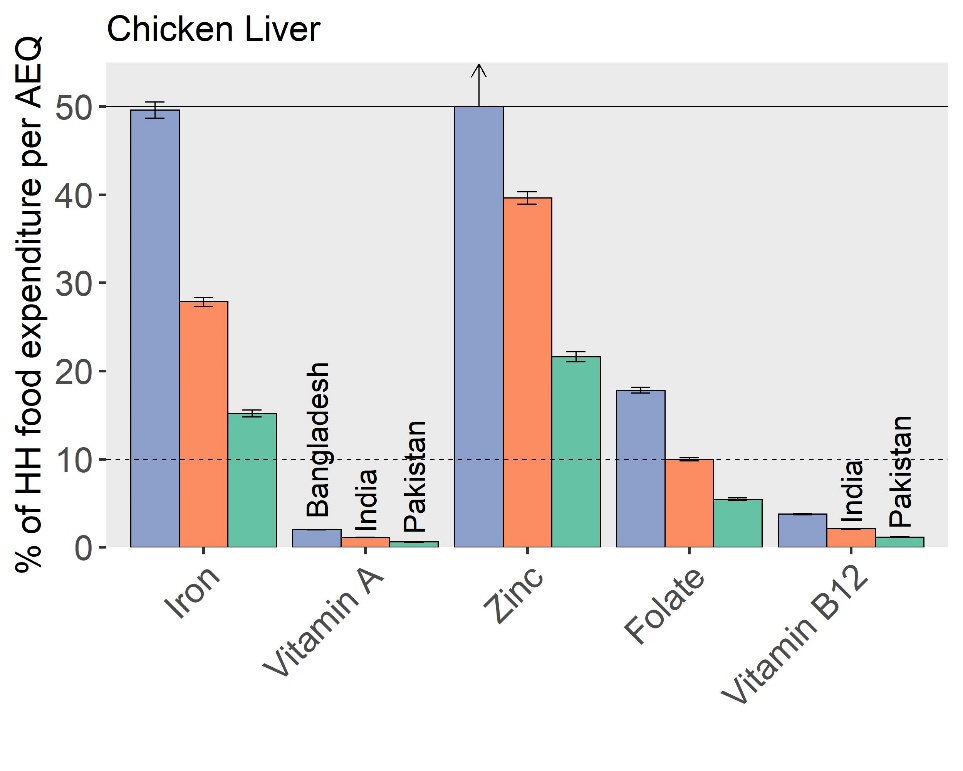

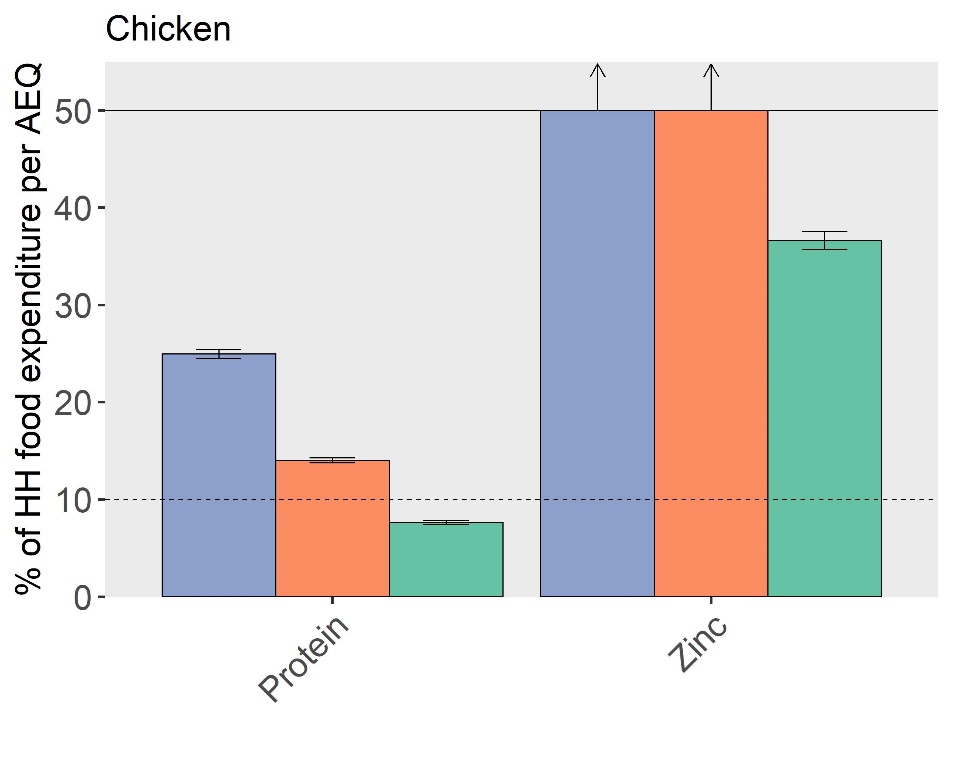

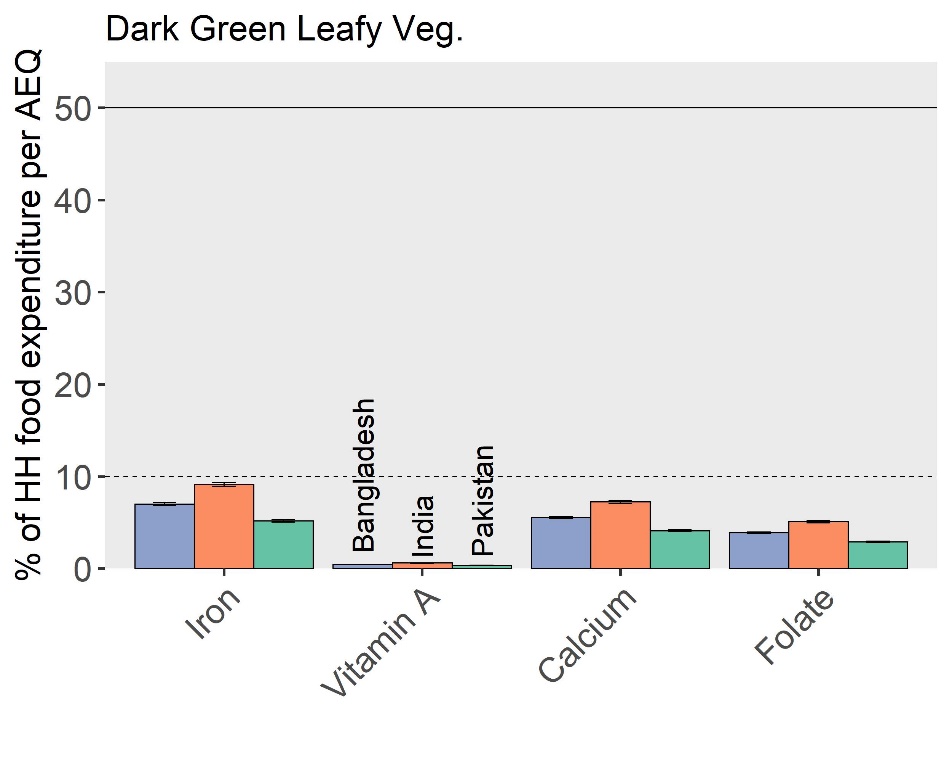

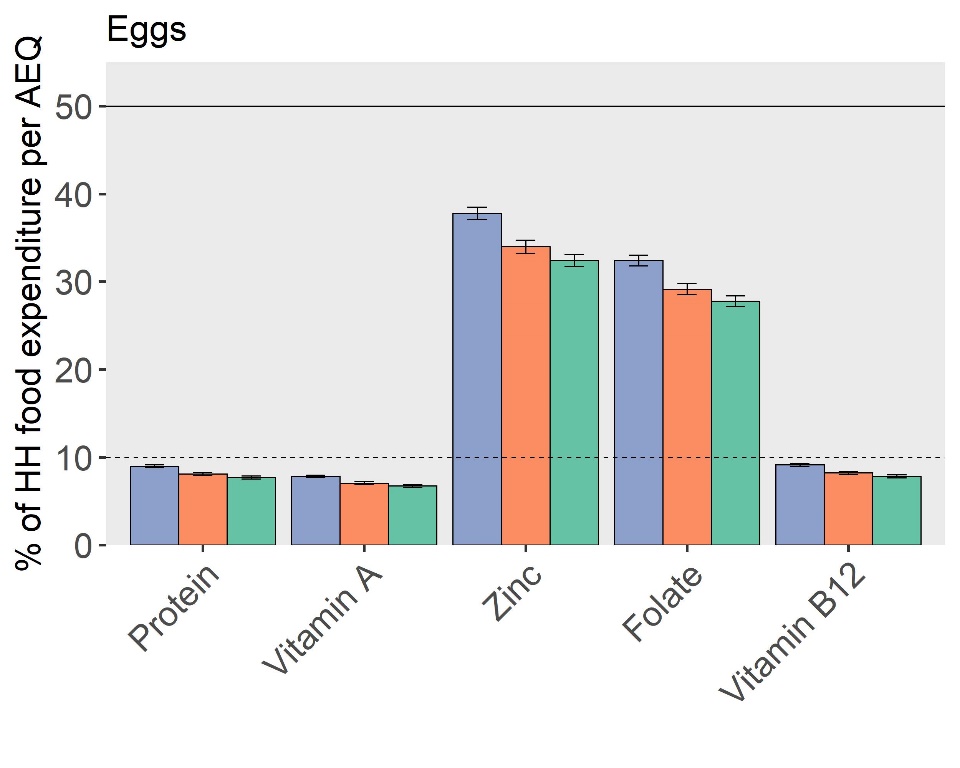

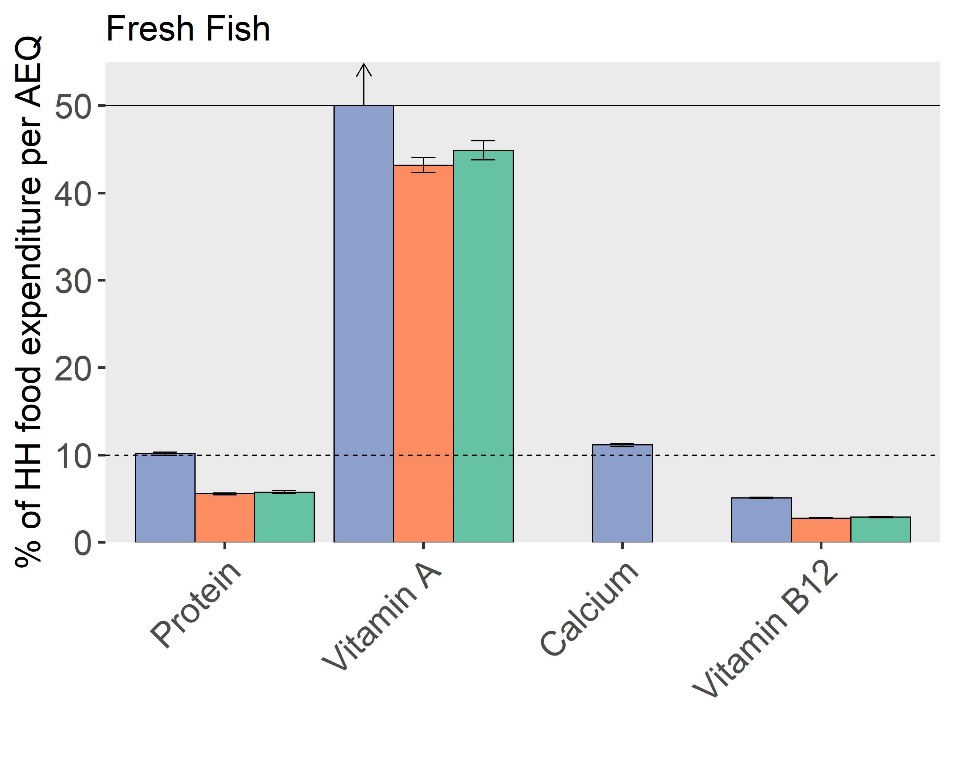

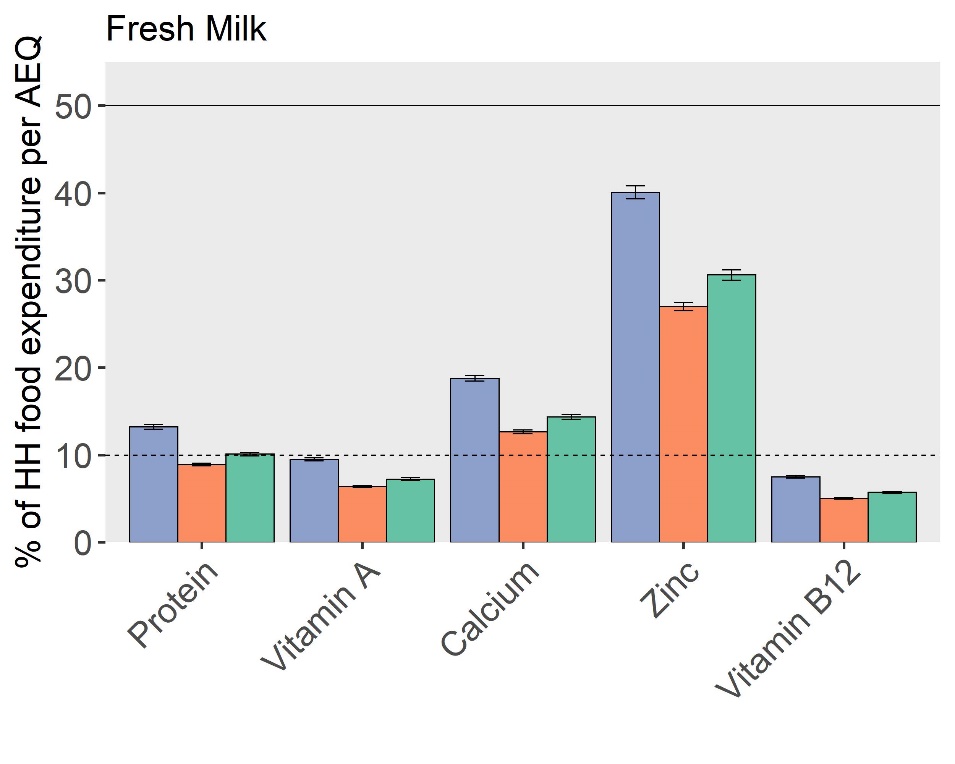

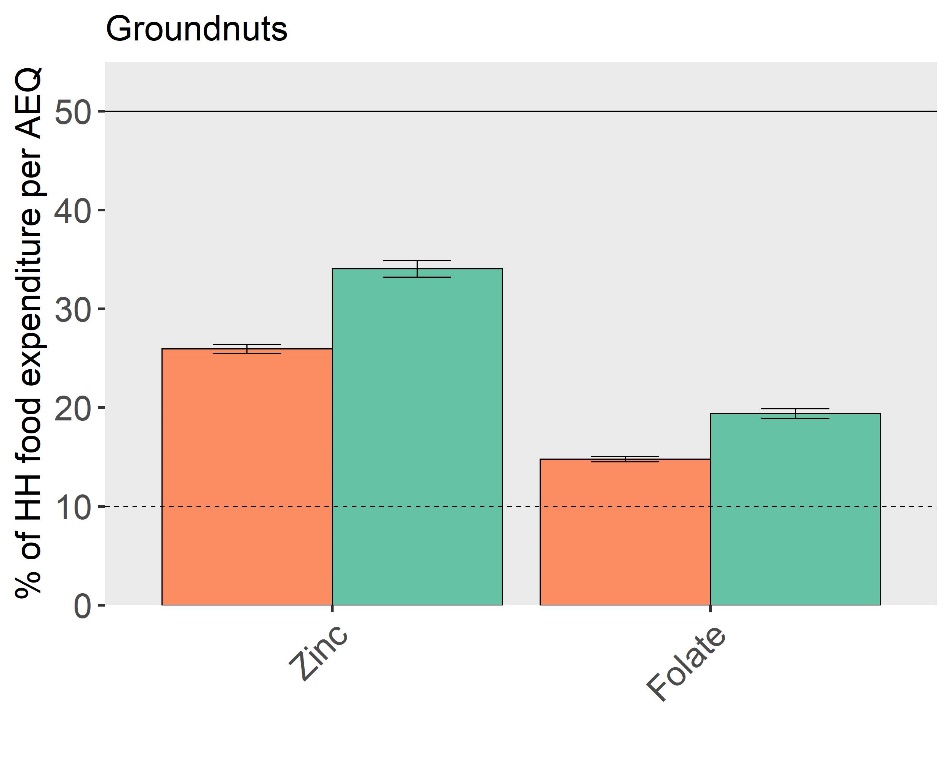

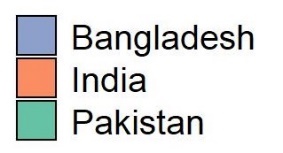

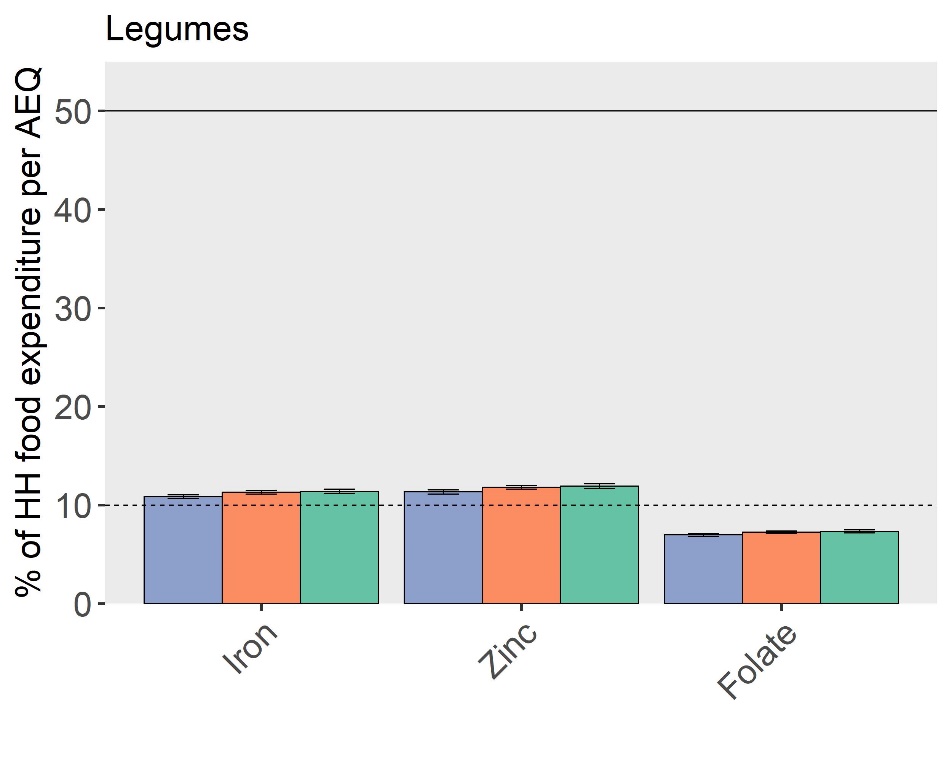

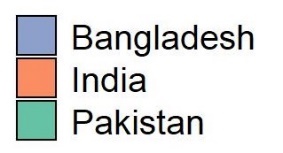

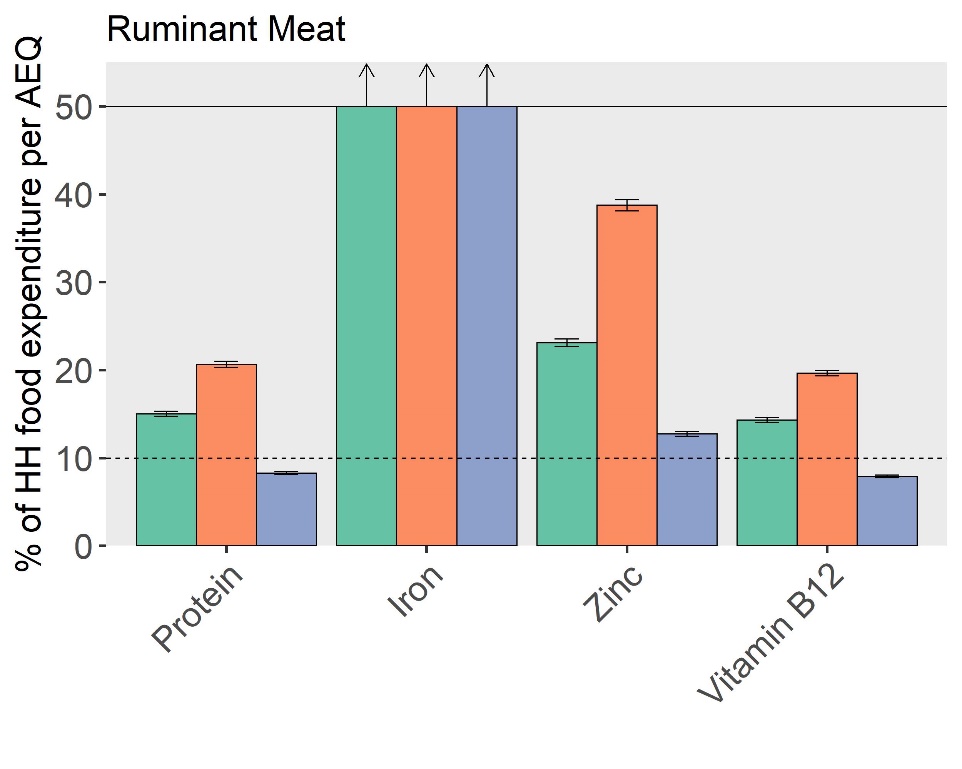

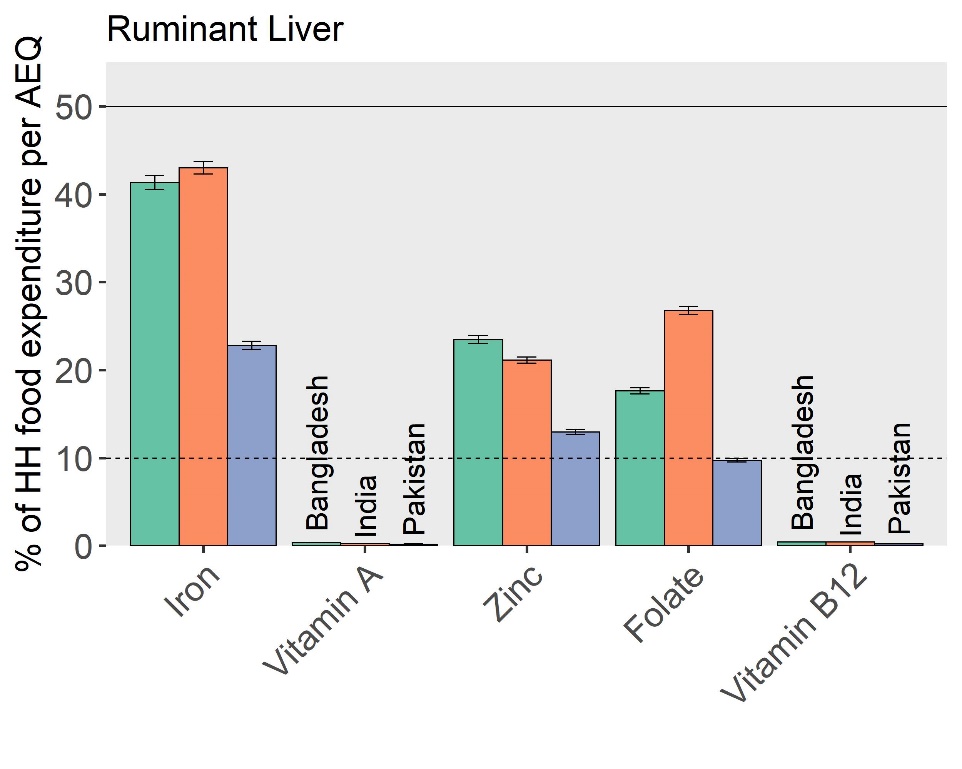

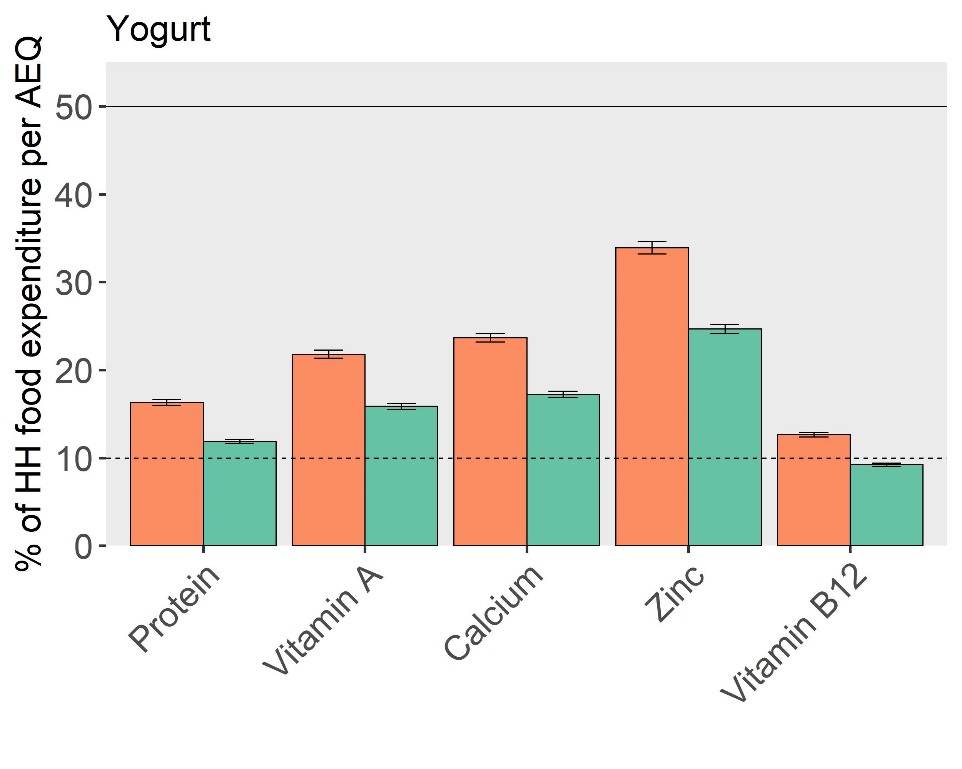
Note: Figure S12 includes only foods that could meet multiple nutrient needs for multiple countries. The y-axis was truncated at 50, but the costs of some foods exceeded 50% of household food expenditure per AEQ; these foods are designated with vertical arrows indicating that the bar continues vertically beyond the scale of the graph. Error bars represent 95% confidence intervals.

# Figure S16: Food prices per kg by country, adjusted for currency exchange rates and purchasing power parities


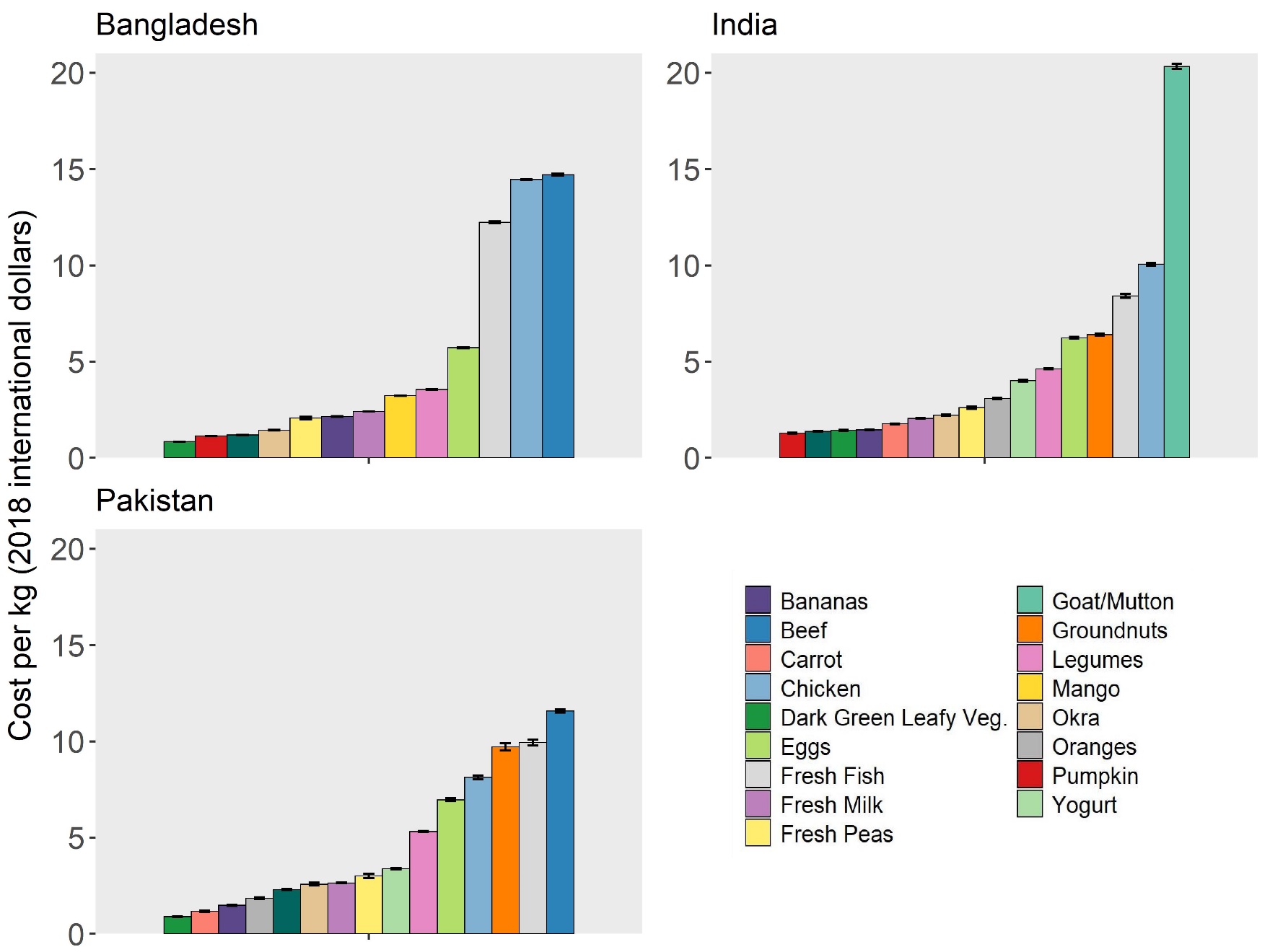


Note: error bars represent 95% confidence intervals

# Figure S17: Sensitivity analysis results for nutrient density and refuse for dark leafy green vegetables, legumes, and chicken liver


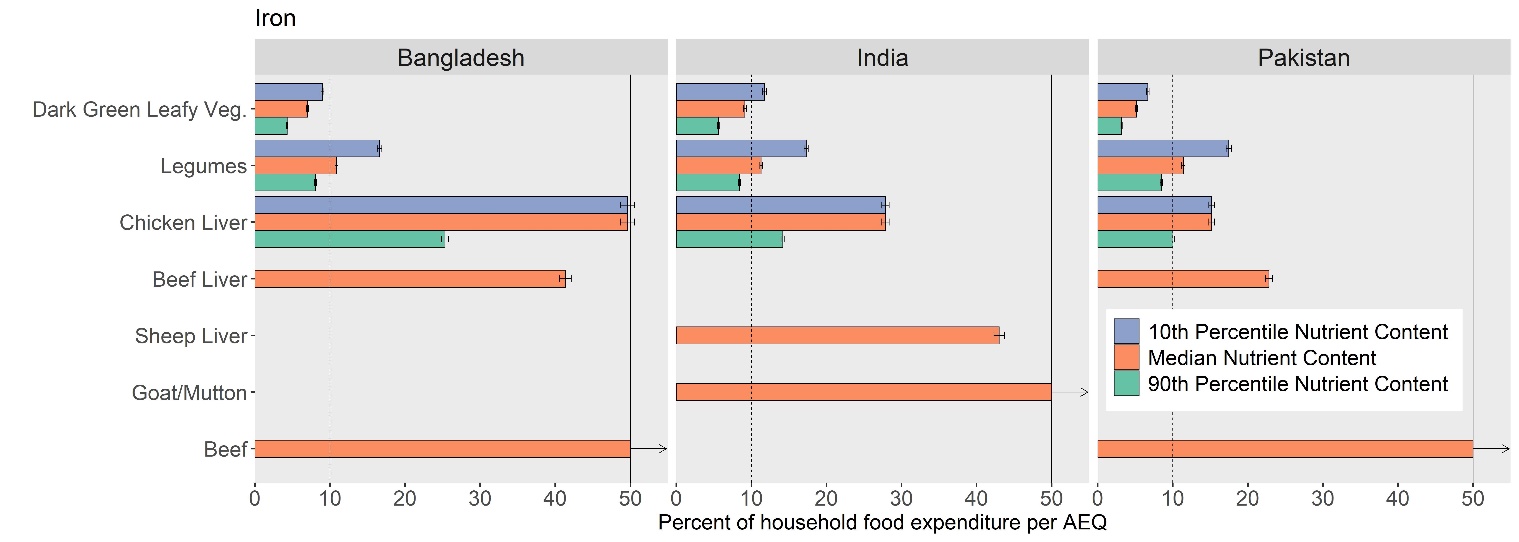


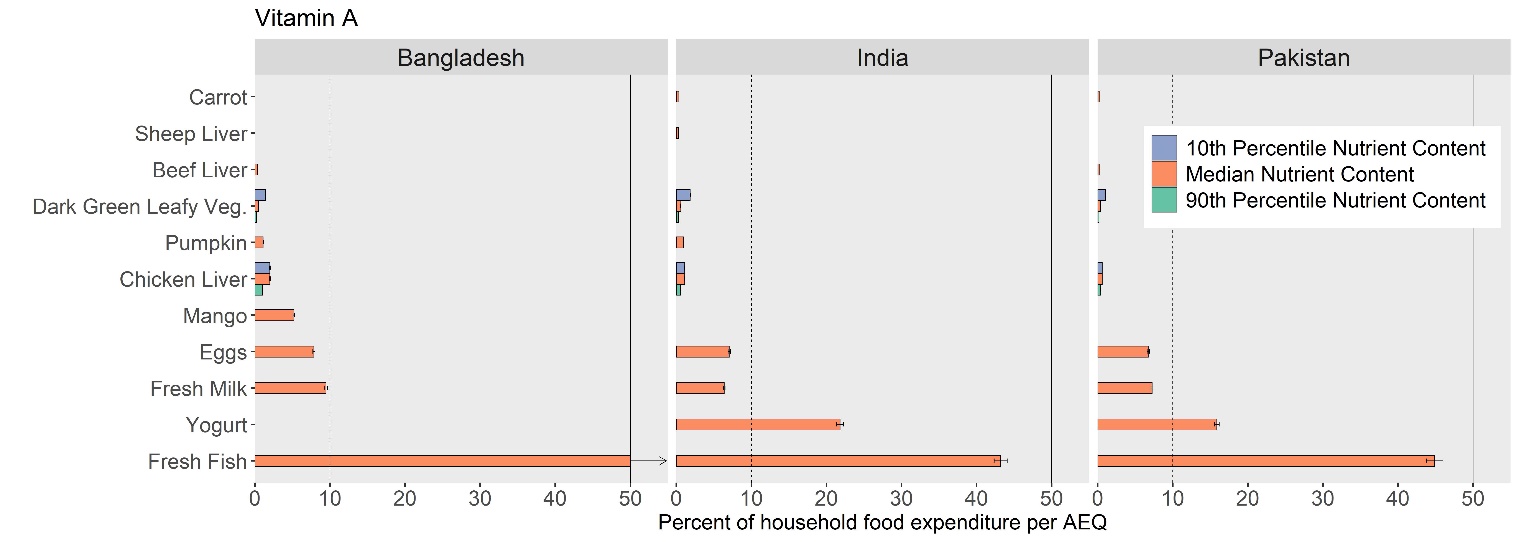


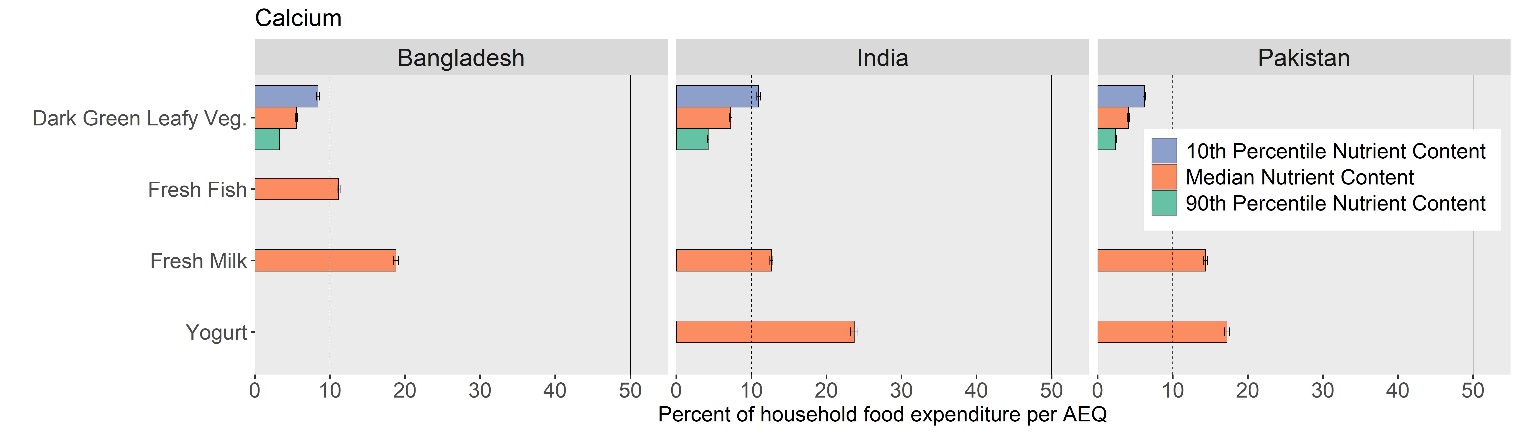


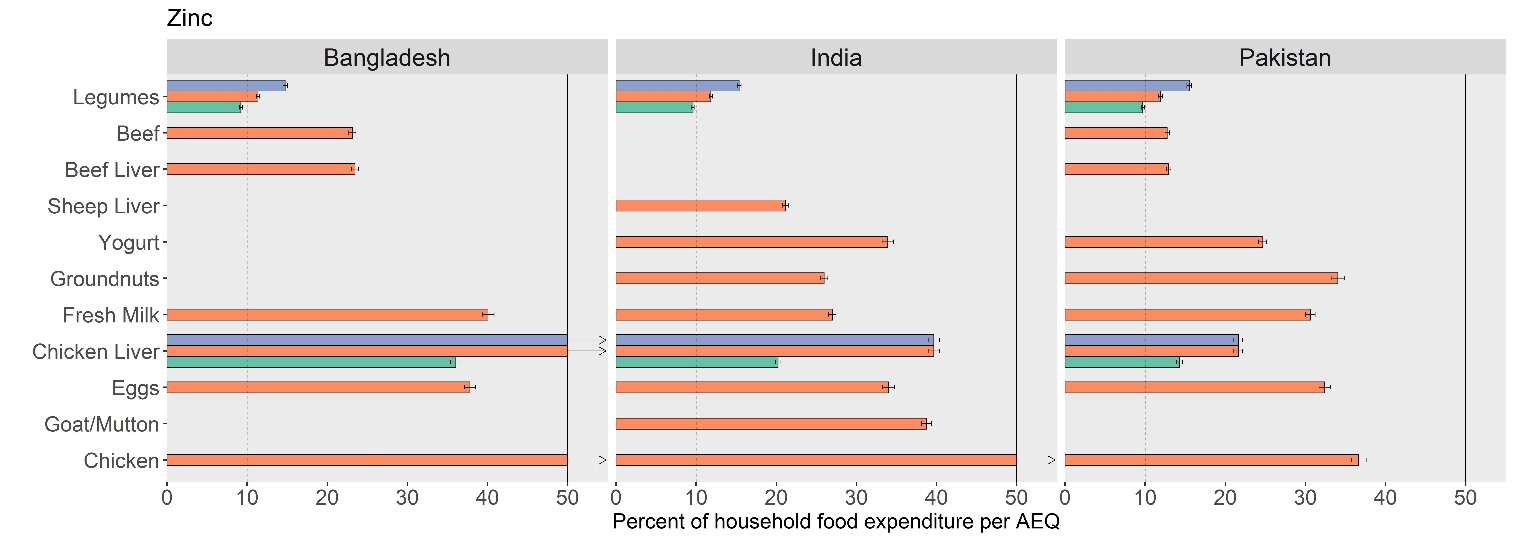


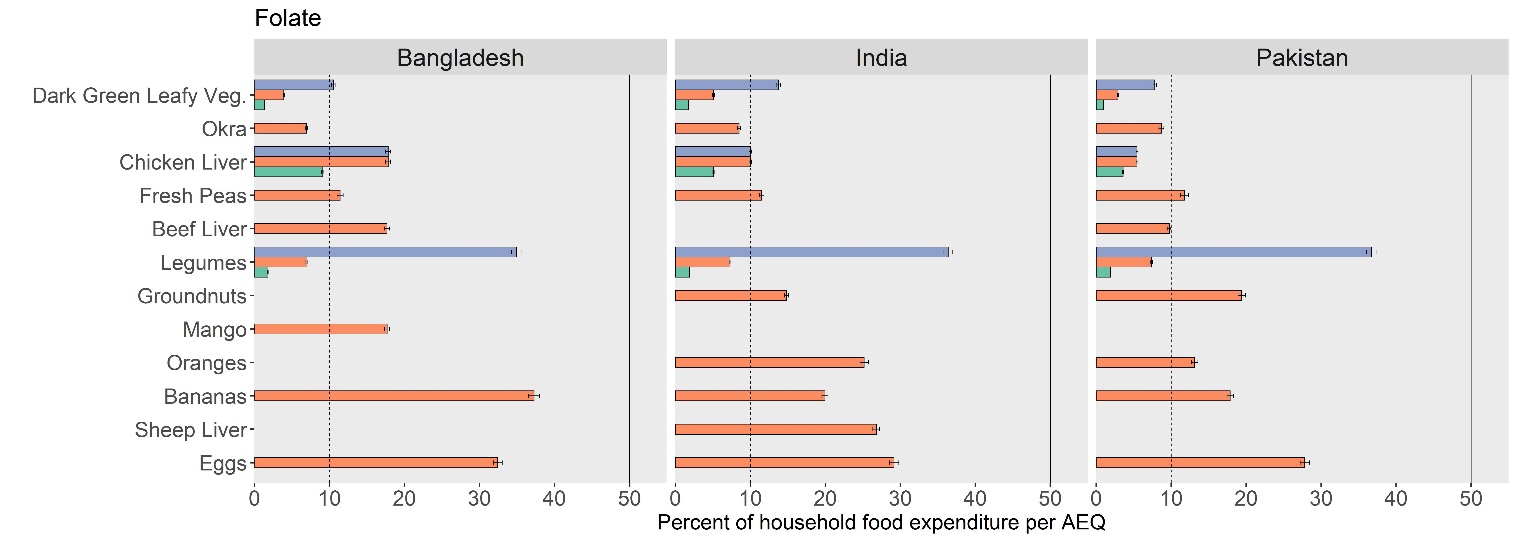

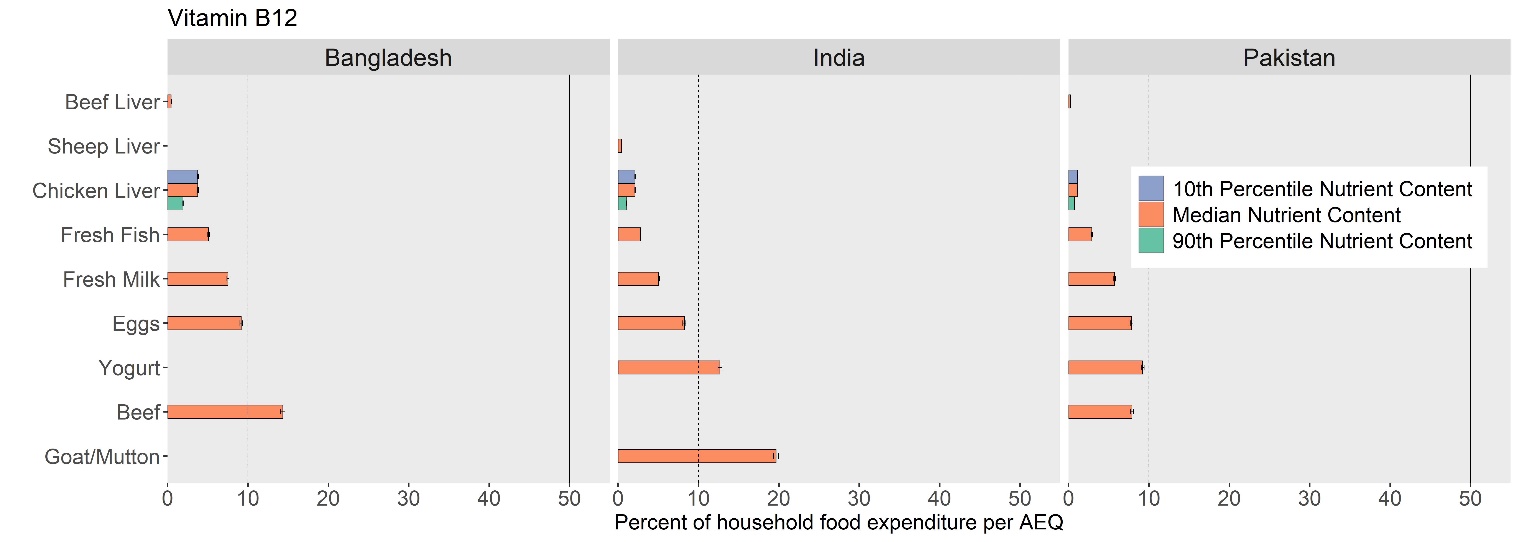


Note: The y-axis was truncated at 50, but the costs of some foods exceeded 50% of household food expenditure per AEQ; these foods are designated with vertical arrows indicating that the bar continues vertically beyond the scale of the graph. Error bars represent 95% confidence intervals.

# Figure S18: Cost per 450 kcal sensitivity analysis results for nutrient density (legumes)


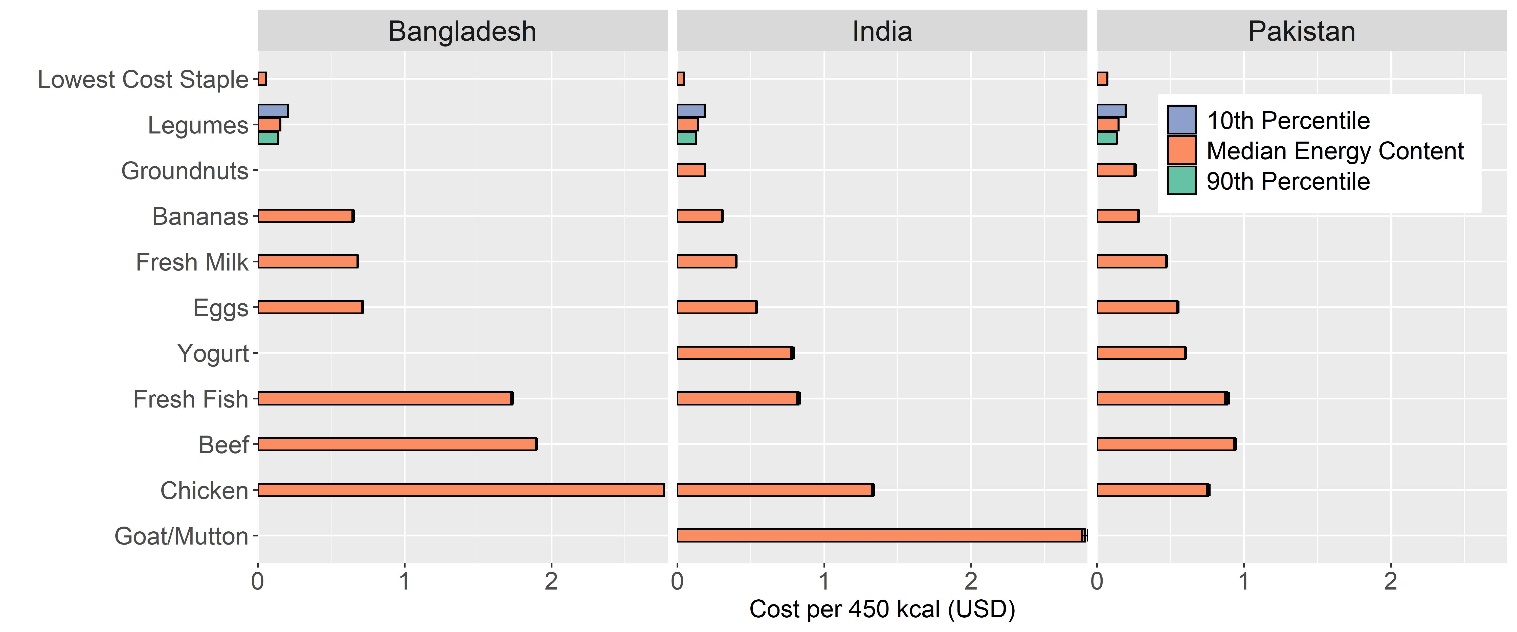


Note: Error bars represent 95% confidence intervals.

# Figure S19: Portion size cost net current expenditure per AEQ, as a share of total household food expenditure per AEQ


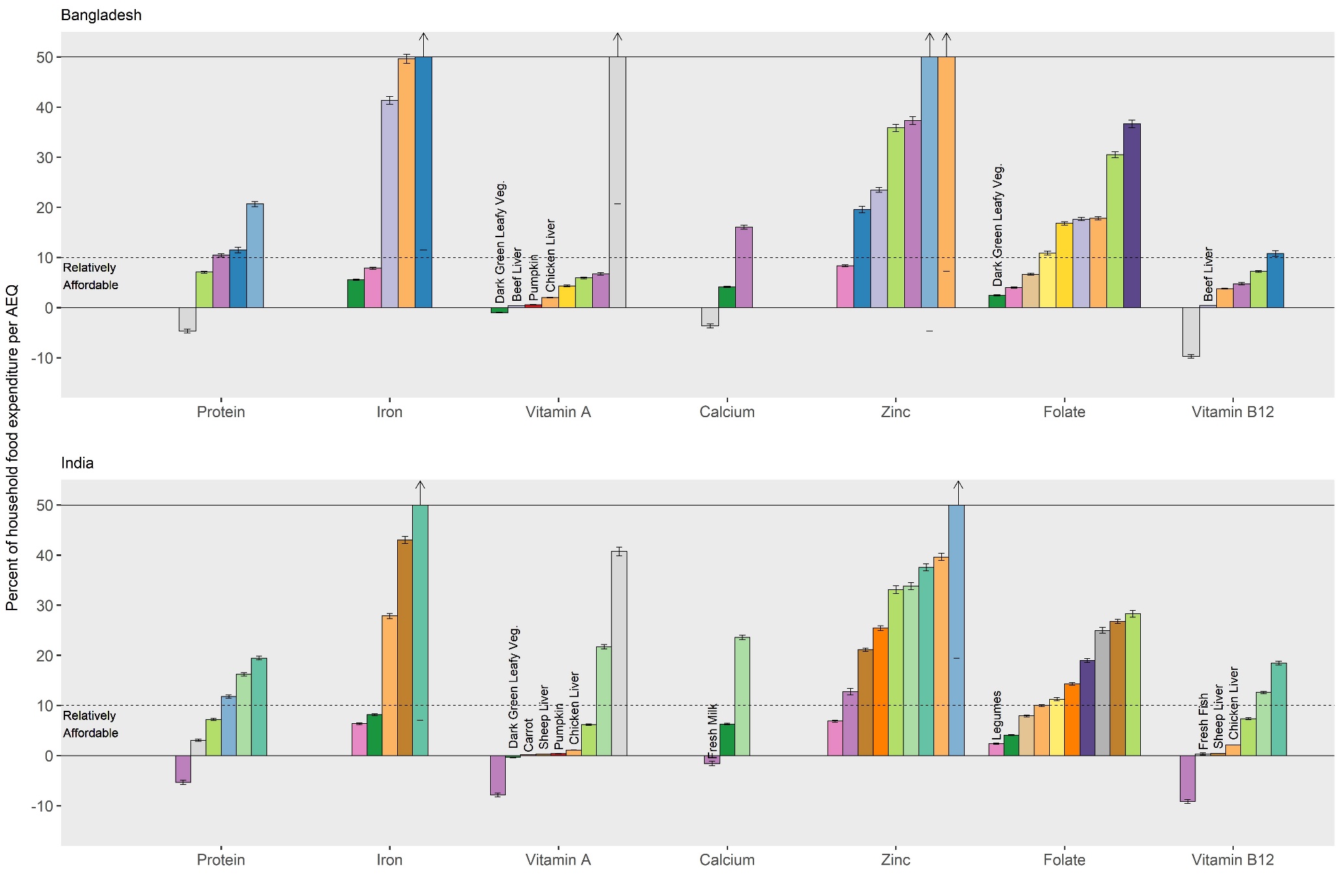


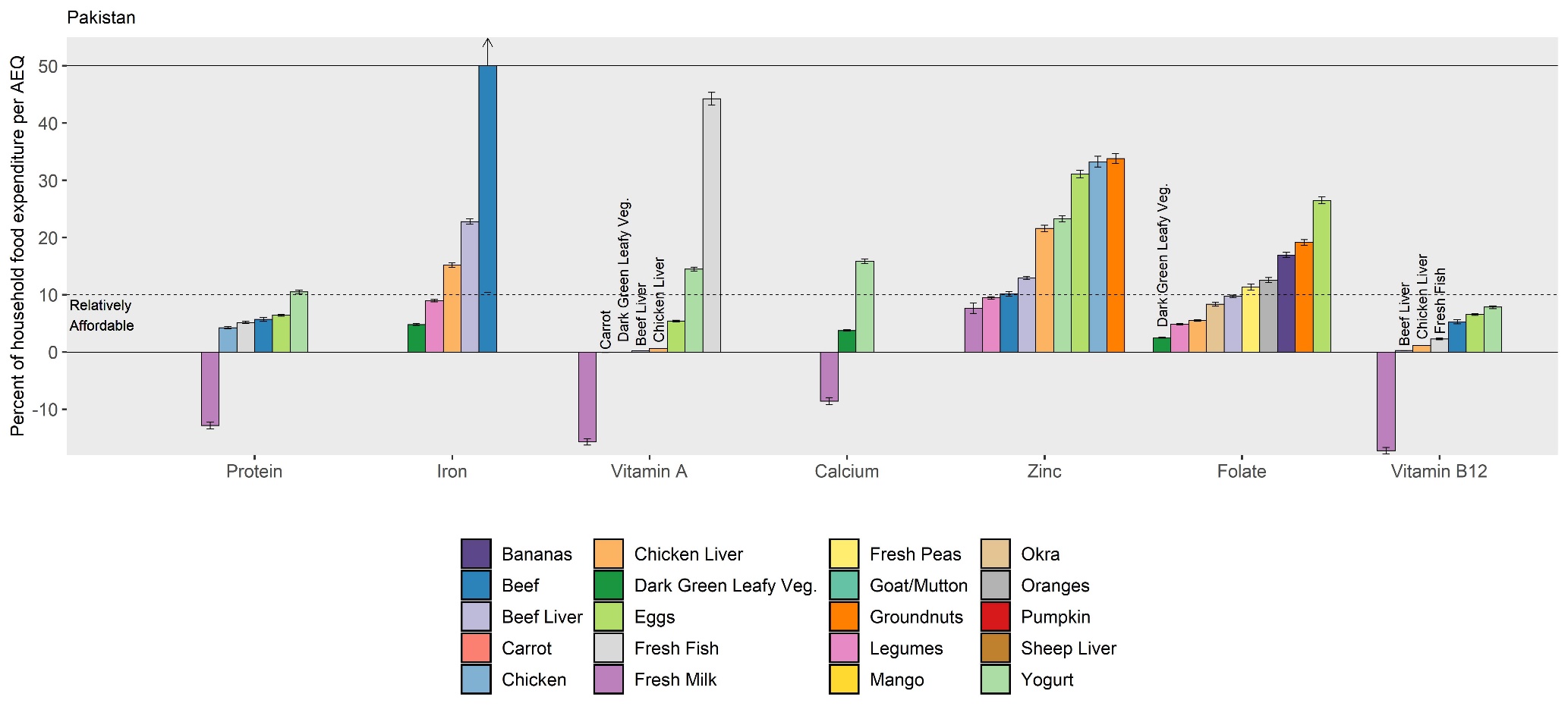


Note: The y-axis was truncated at 50, but the costs of some foods exceeded 50% of household food expenditure per AEQ; these foods are designated with vertical arrows indicating that the bar continues vertically beyond the scale of the graph. Error bars represent 95% confidence intervals.

# Figure S20: Portion size cost, as a share of total household food expenditure per adult equivalent, by rural/urban setting


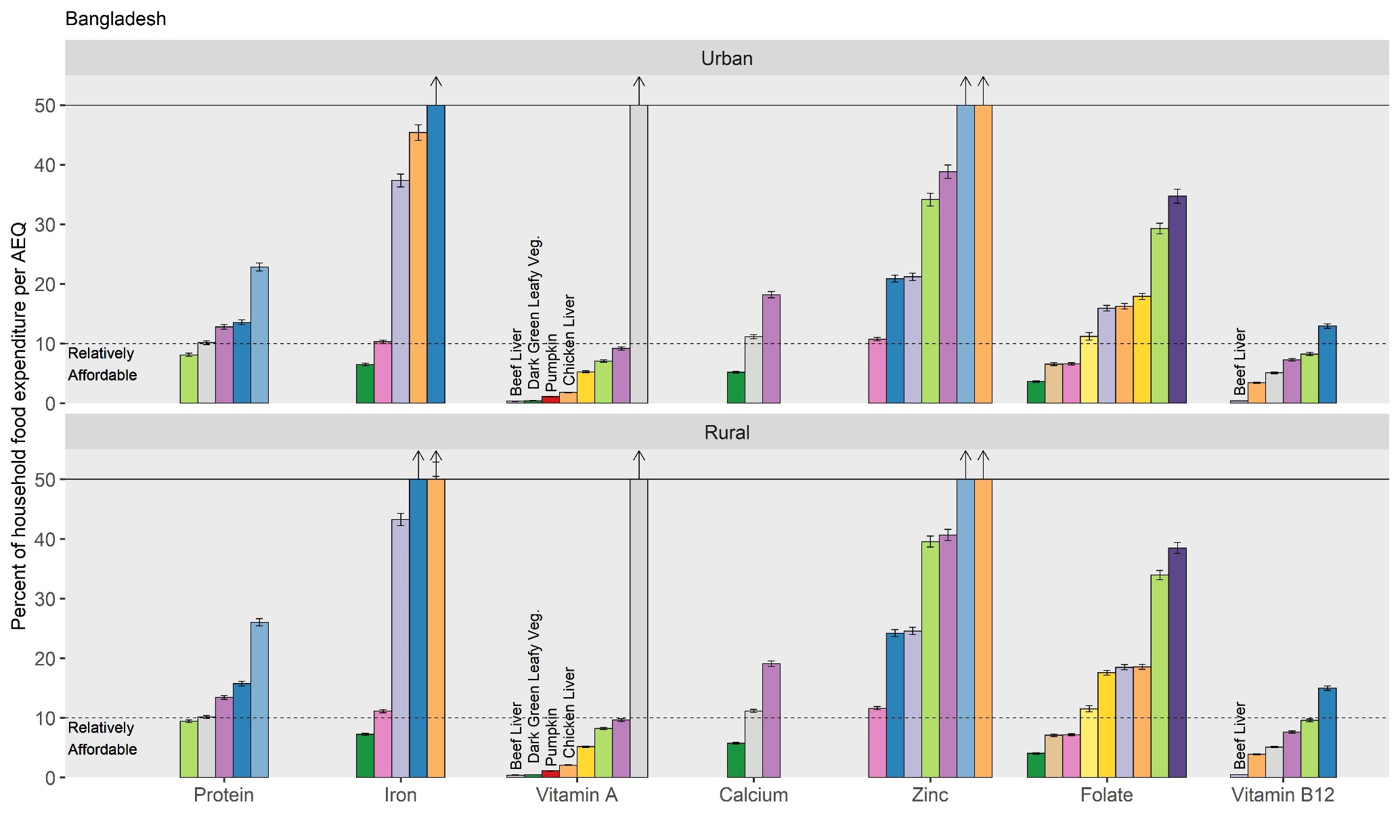

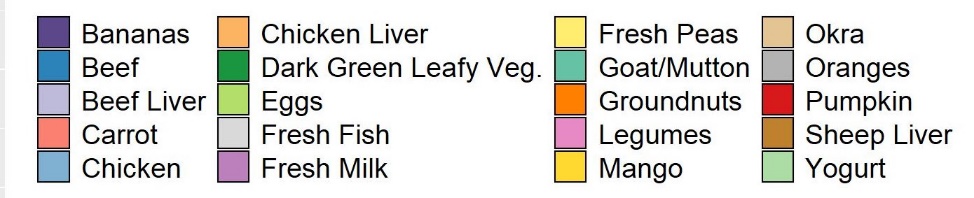


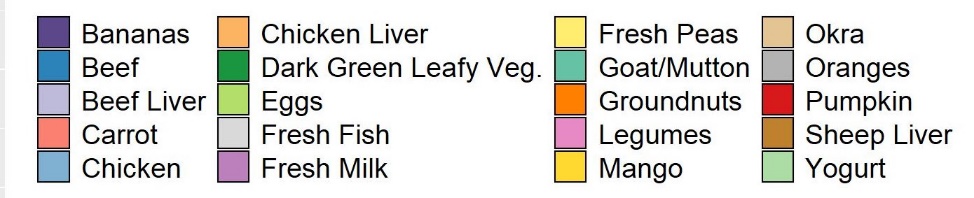


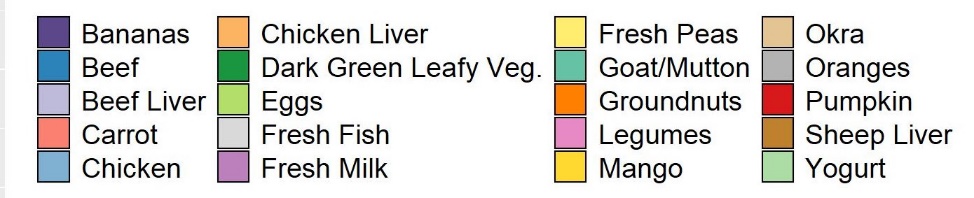


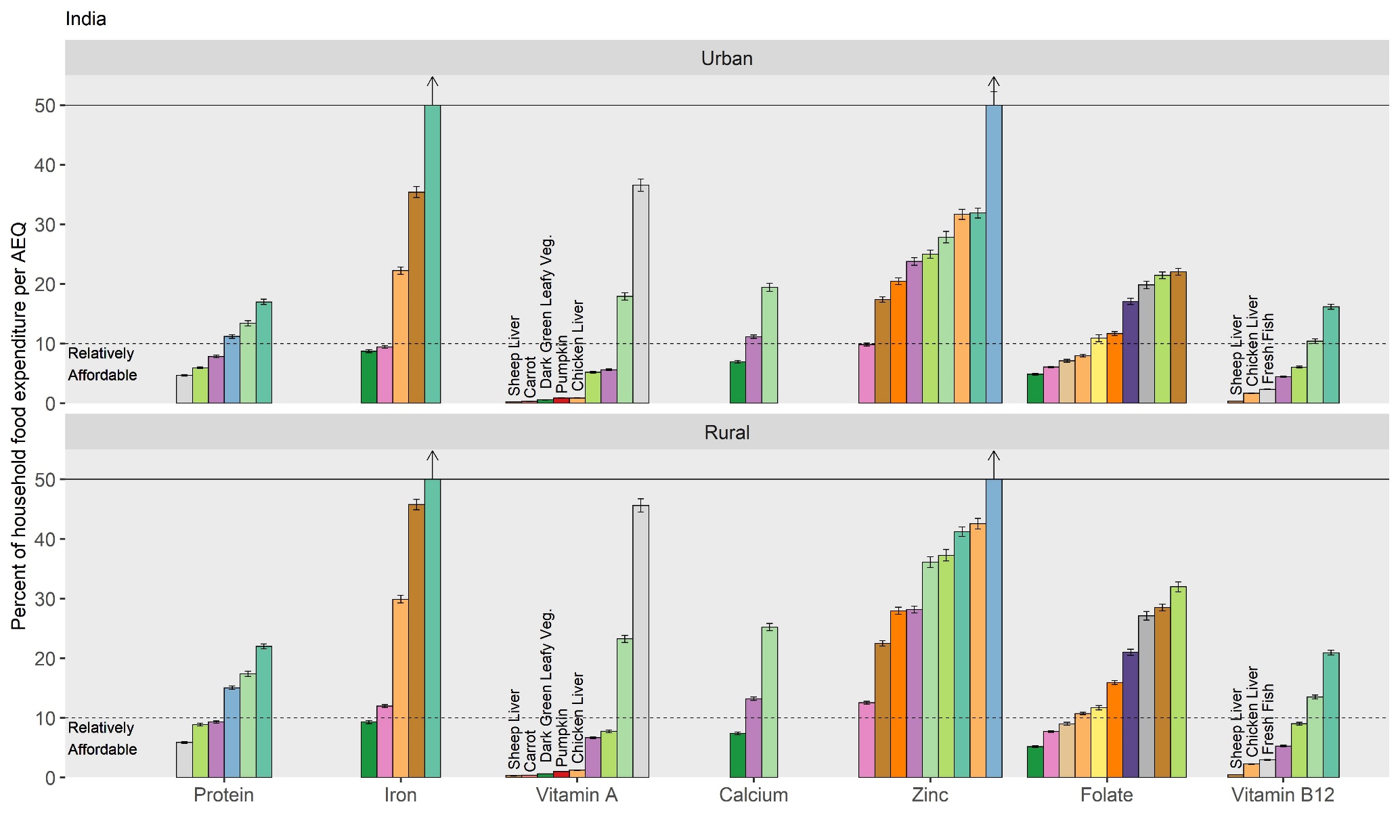

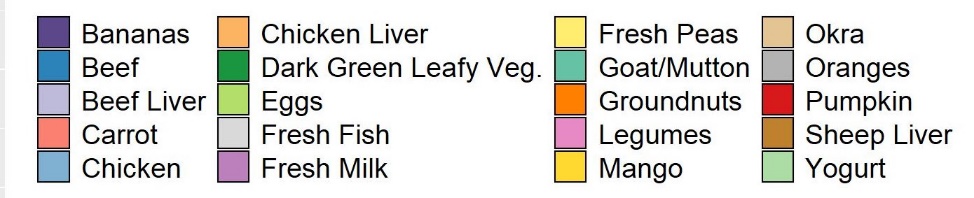


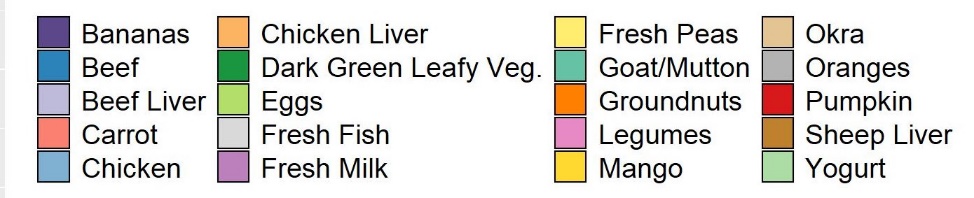

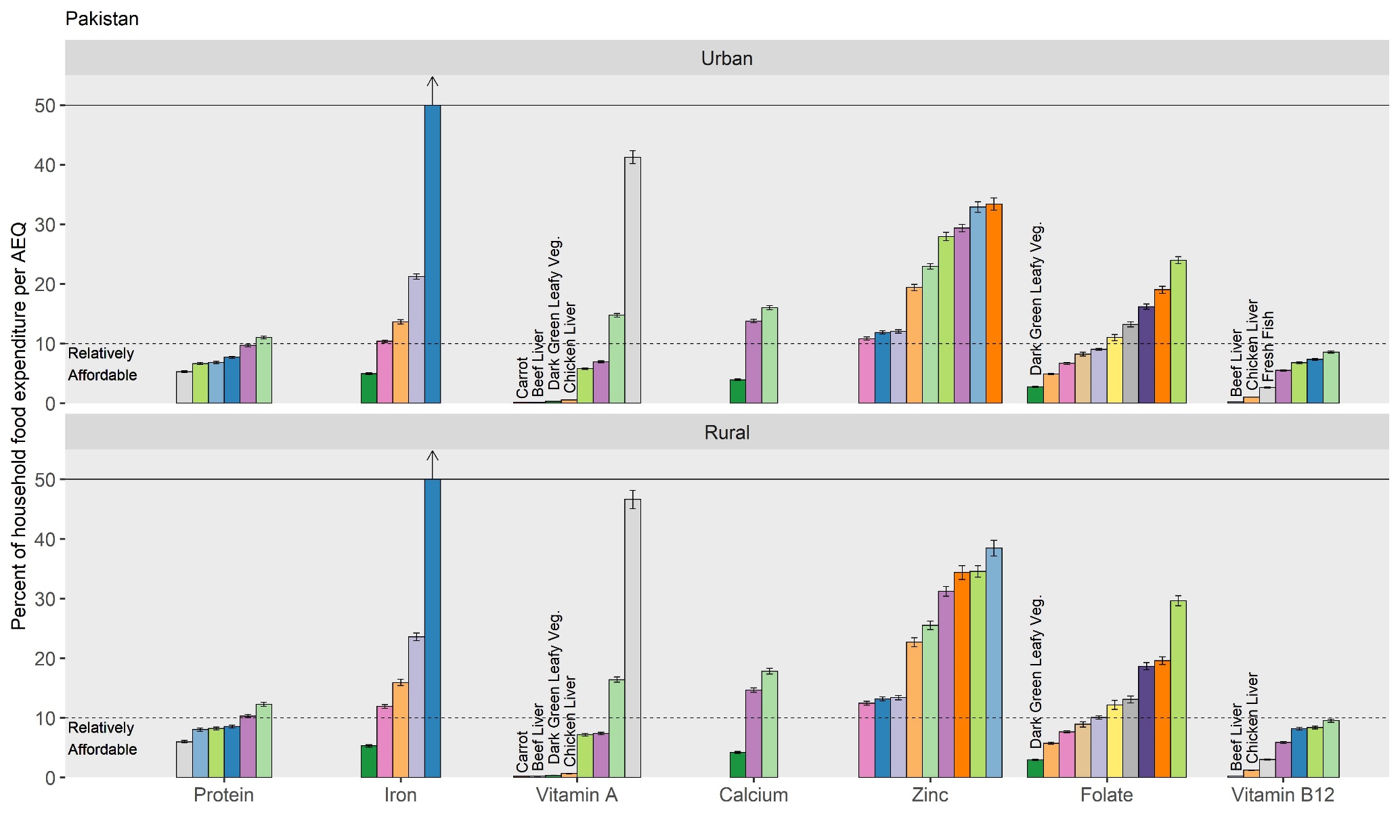


Note: The y-axis was truncated at 50, but the costs of some foods exceeded 50% of household food expenditure per AEQ; these foods are designated with vertical arrows indicating that the bar continues vertically beyond the scale of the graph. Error bars represent 95% confidence intervals.

Figure S21: Portion size cost, as a share of total household food expenditure per adult equivalent, by quintile


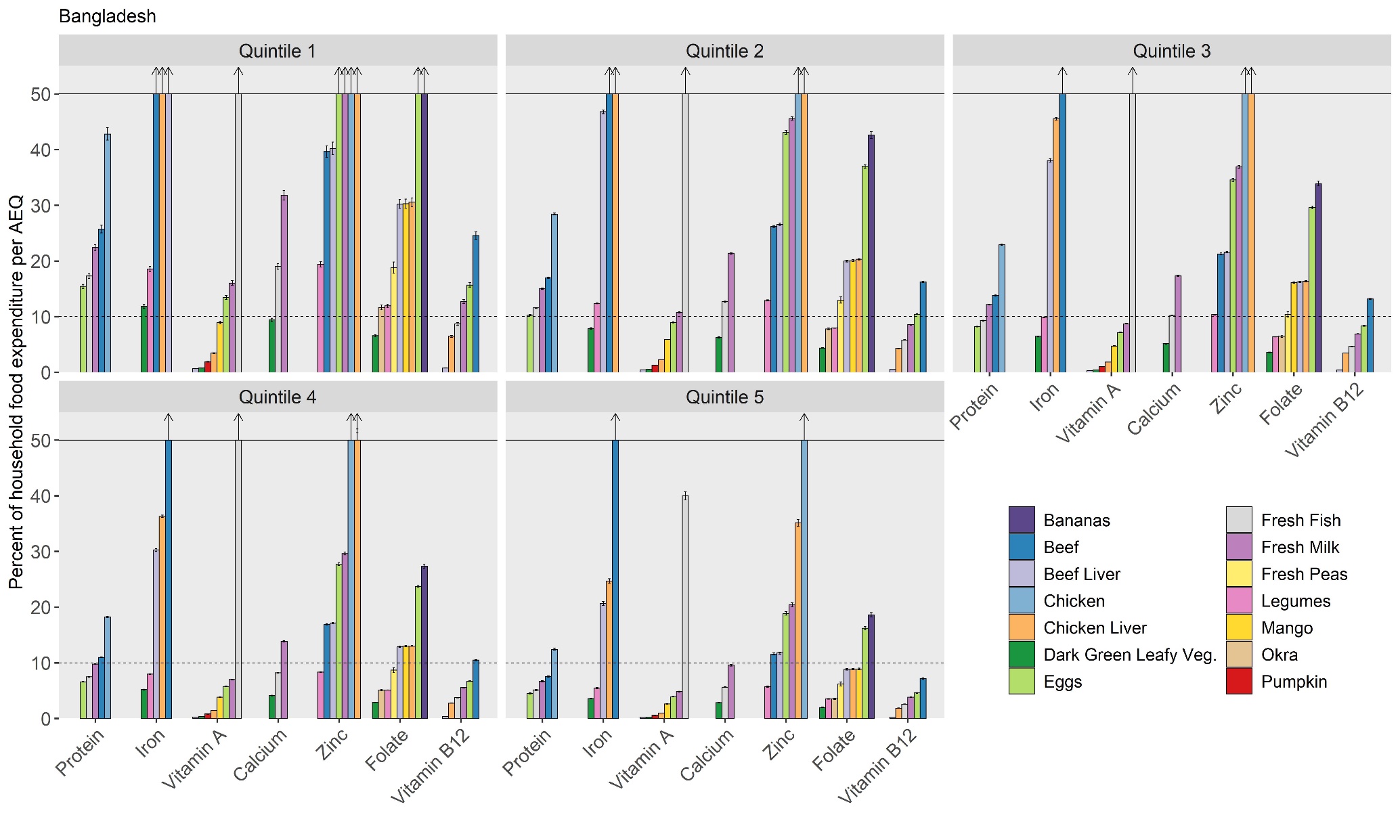


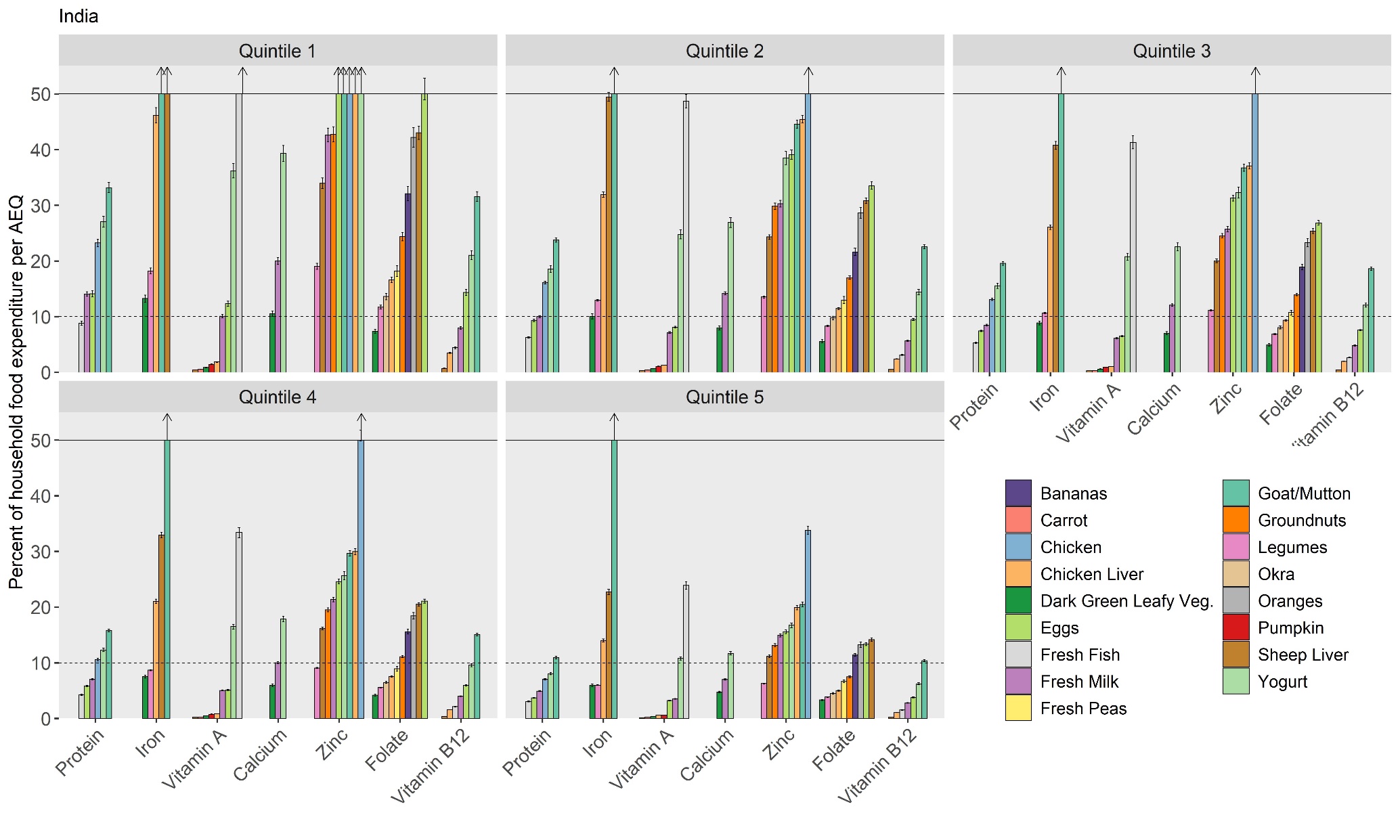


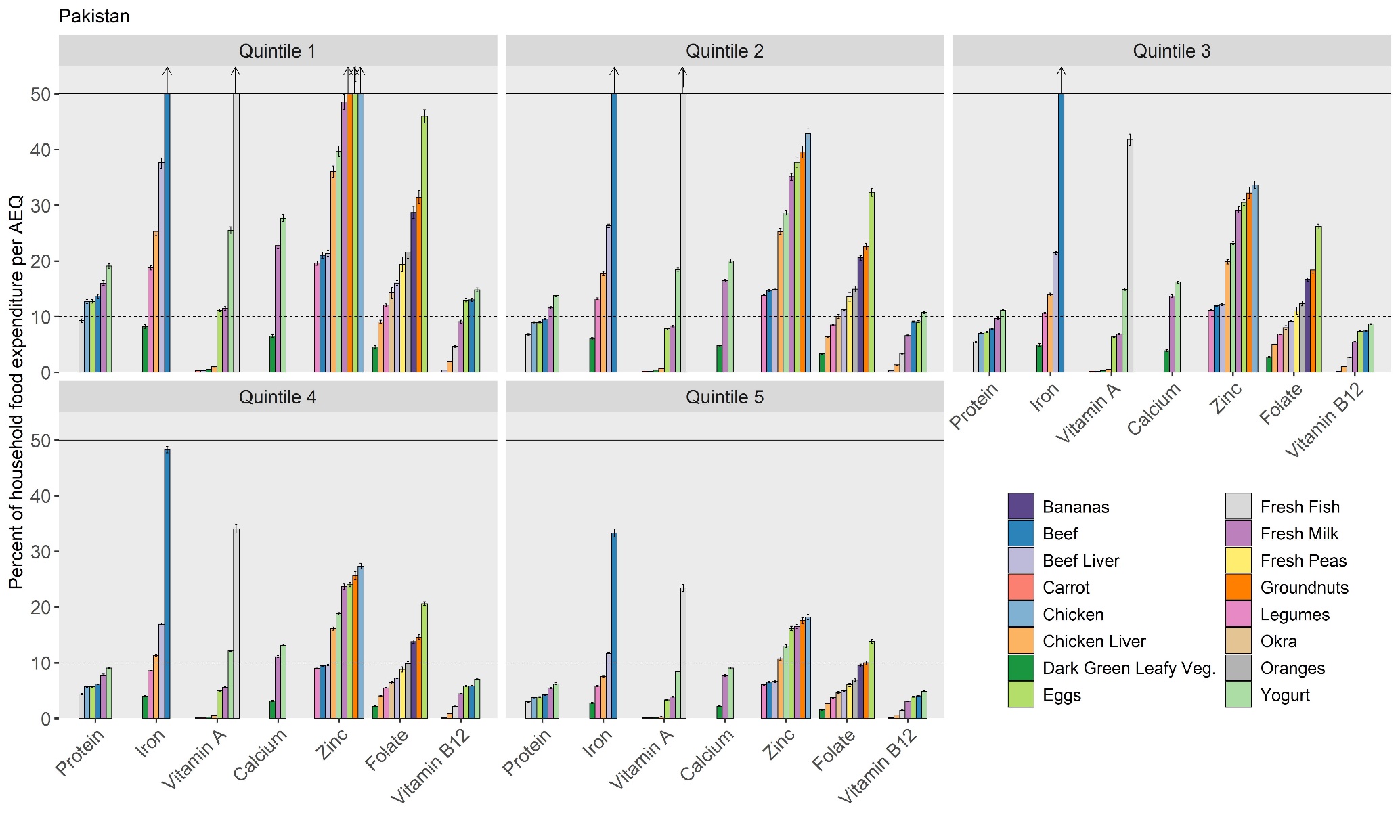


Note: The y-axis was truncated at 50, but the costs of some foods exceeded 50% of household food expenditure per AEQ; these foods are designated with vertical arrows indicating that the bar continues vertically beyond the scale of the graph. Error bars represent 95% confidence intervals. Quintiles are based on household food expenditures per AEQ.

# Figure S22: Food cost per 450 kcal by rural/urban setting


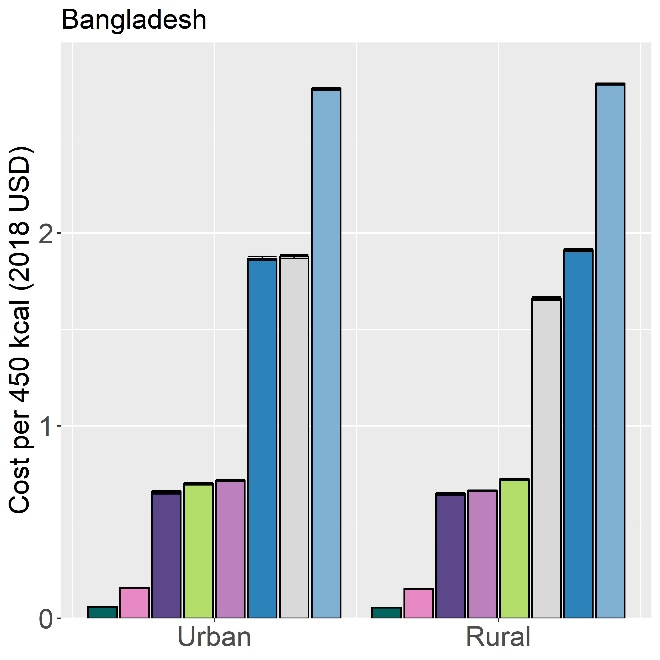

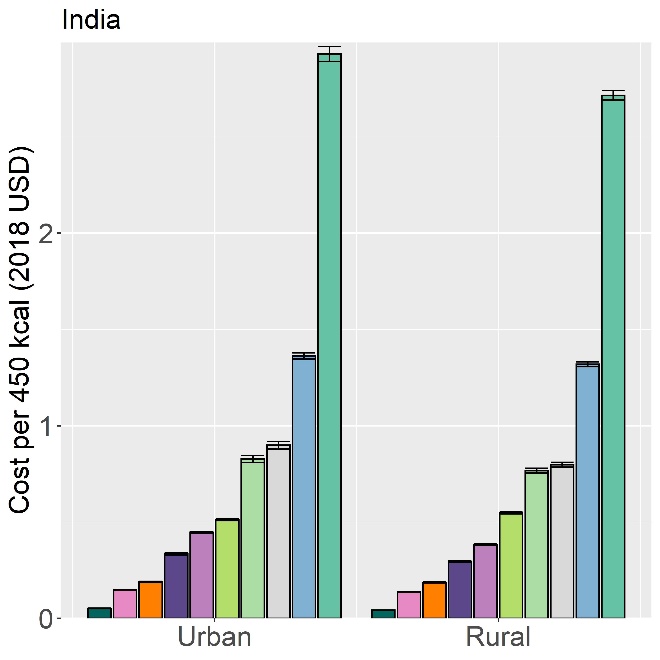

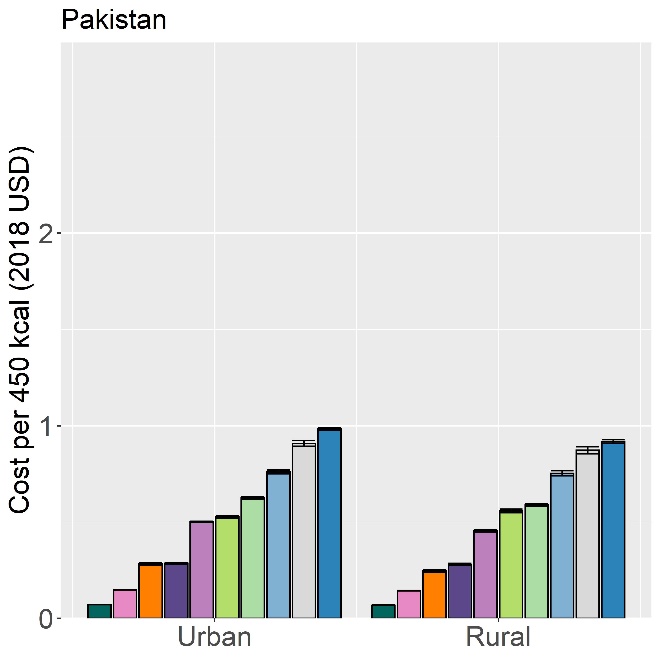


**
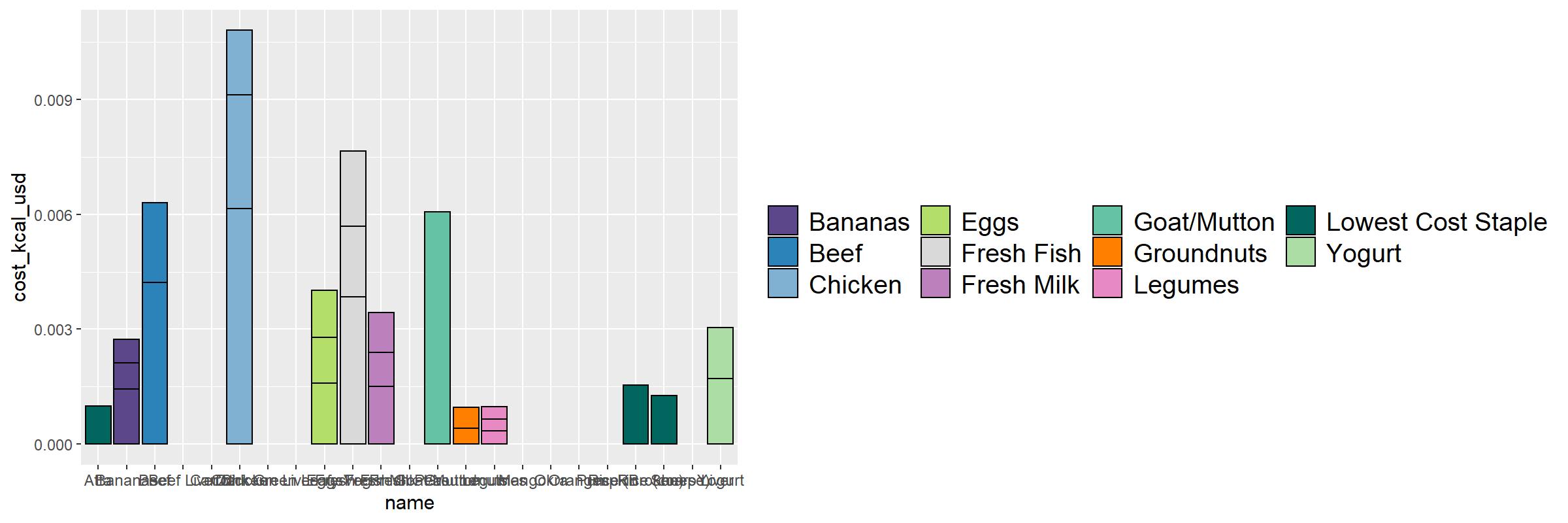
**

Note: the lowest cost staple was rice in Bangladesh and Pakistan and wheat flour in India. Error bars represent 95% confidence intervals.

# Figure S23: Food cost per 450 kcal by quintile

**
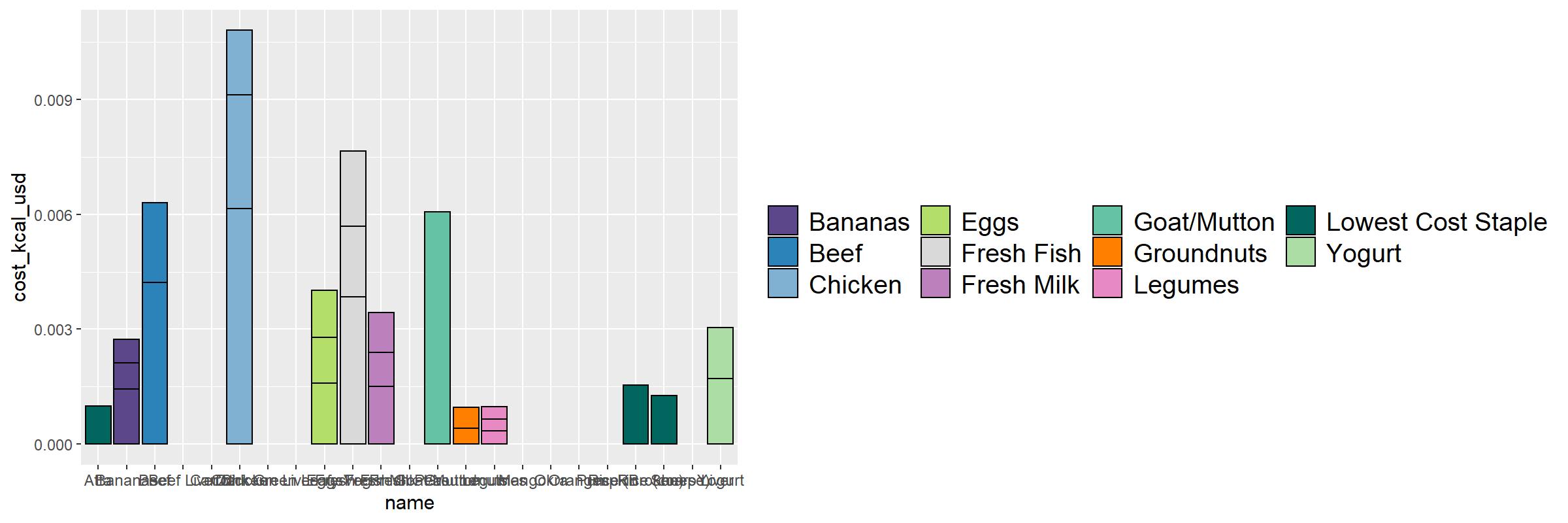
**
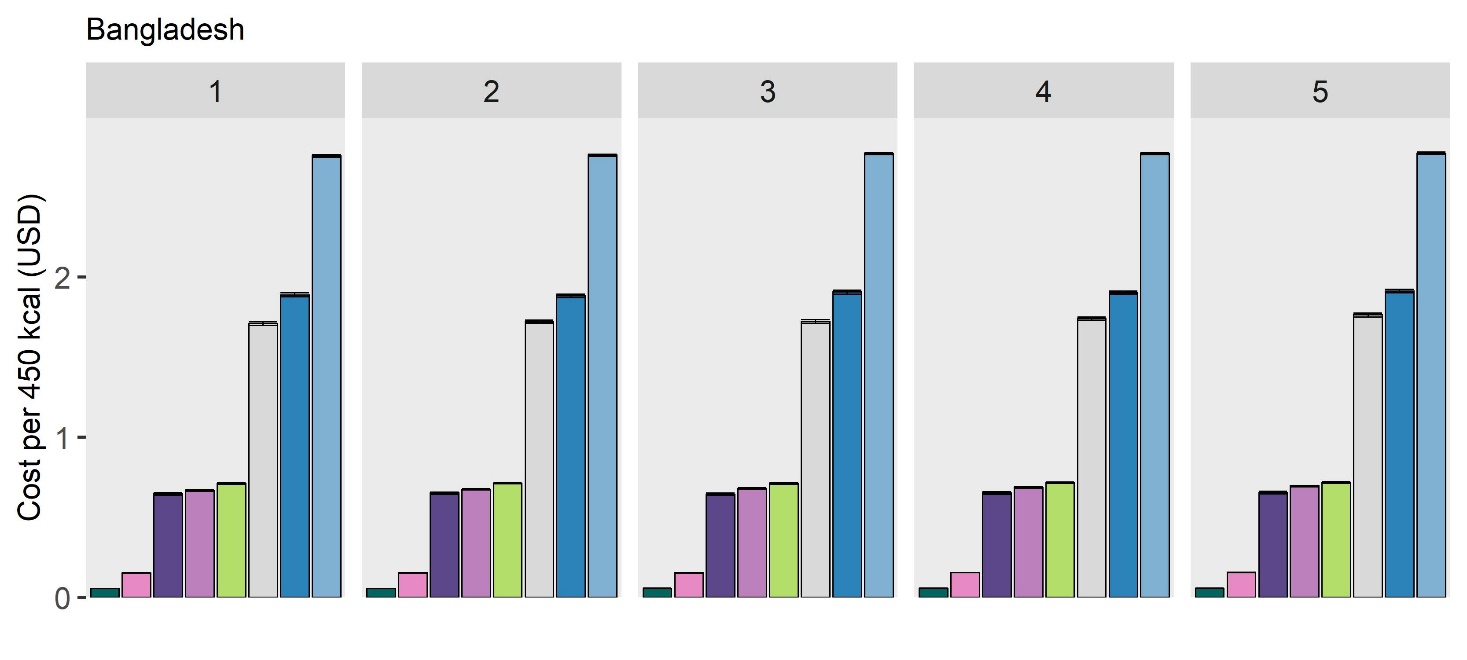

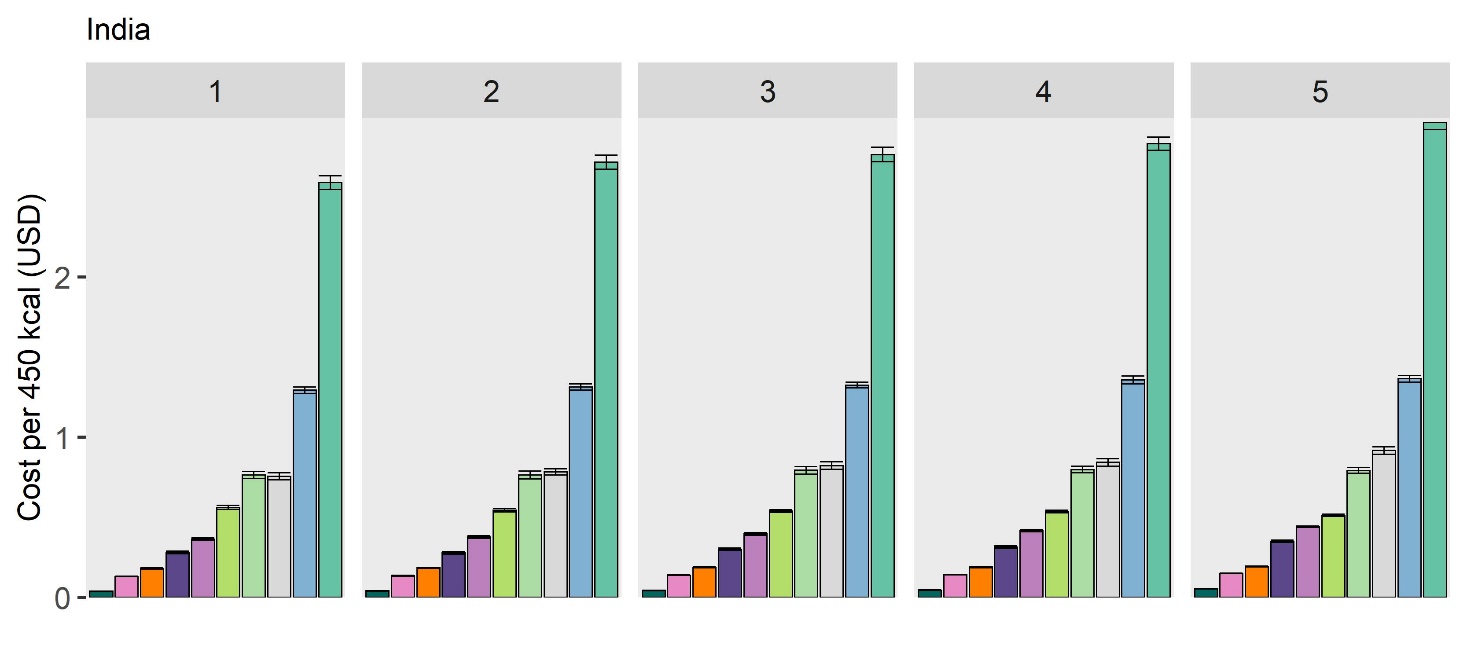

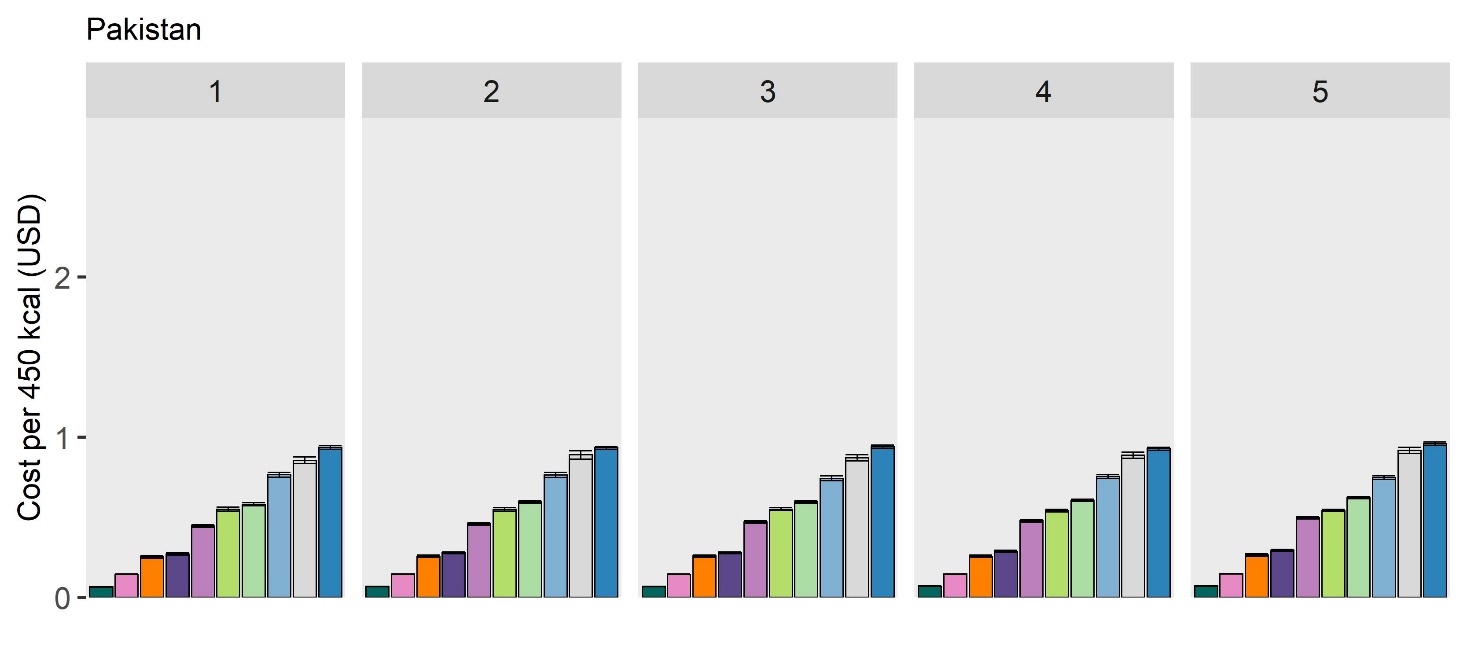


Note: the lowest cost staple was rice in Bangladesh and Pakistan and wheat flour in India. Error bars represent 95% confidence intervals. Quintiles are based on household food expenditures per AEQ.

Figure S24: Average share of micronutrient requirements affordability analysis, by rural/urban setting

**
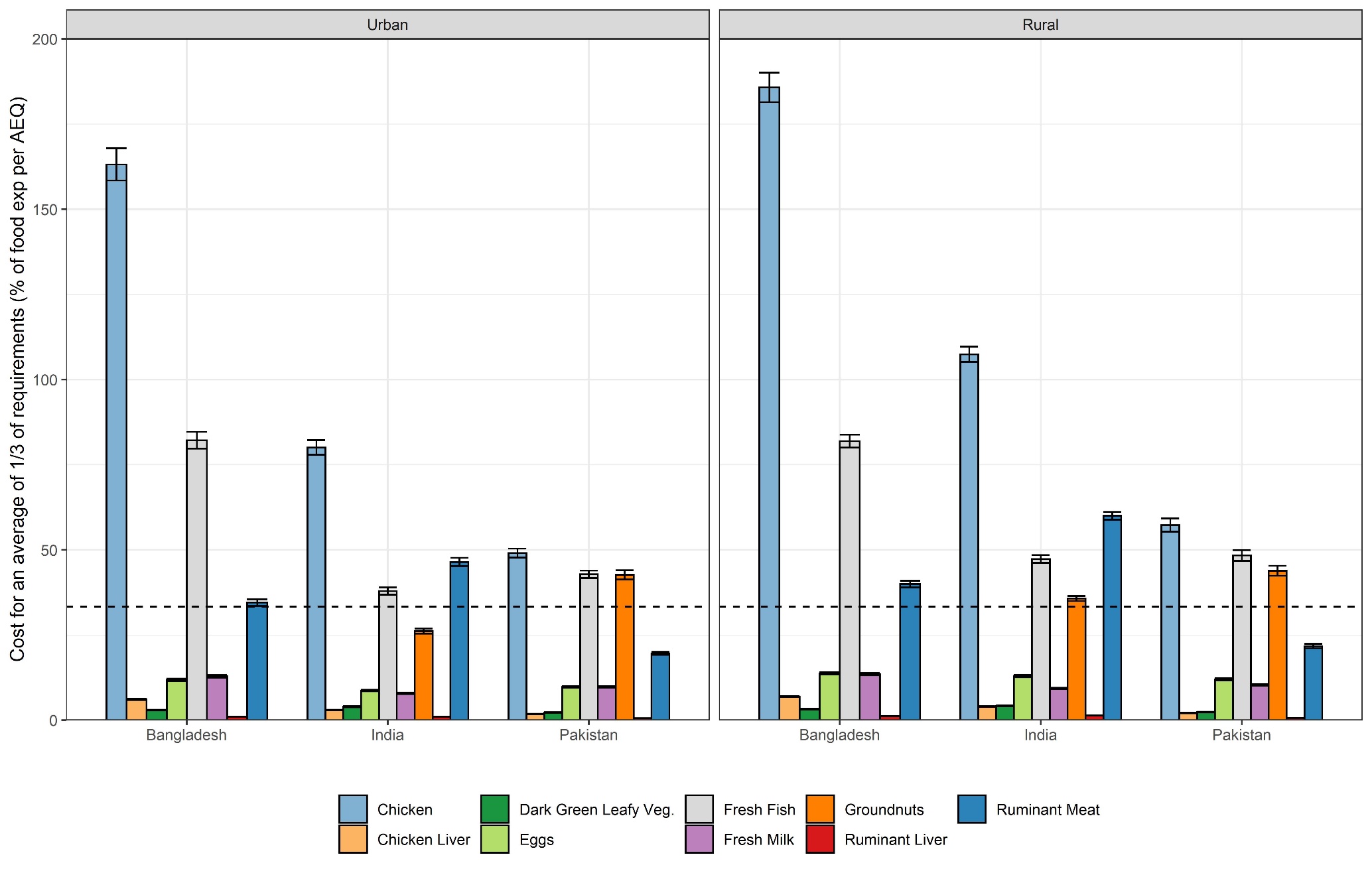
**

Figure S25: Average share of micronutrient requirements affordability analysis, by quintile


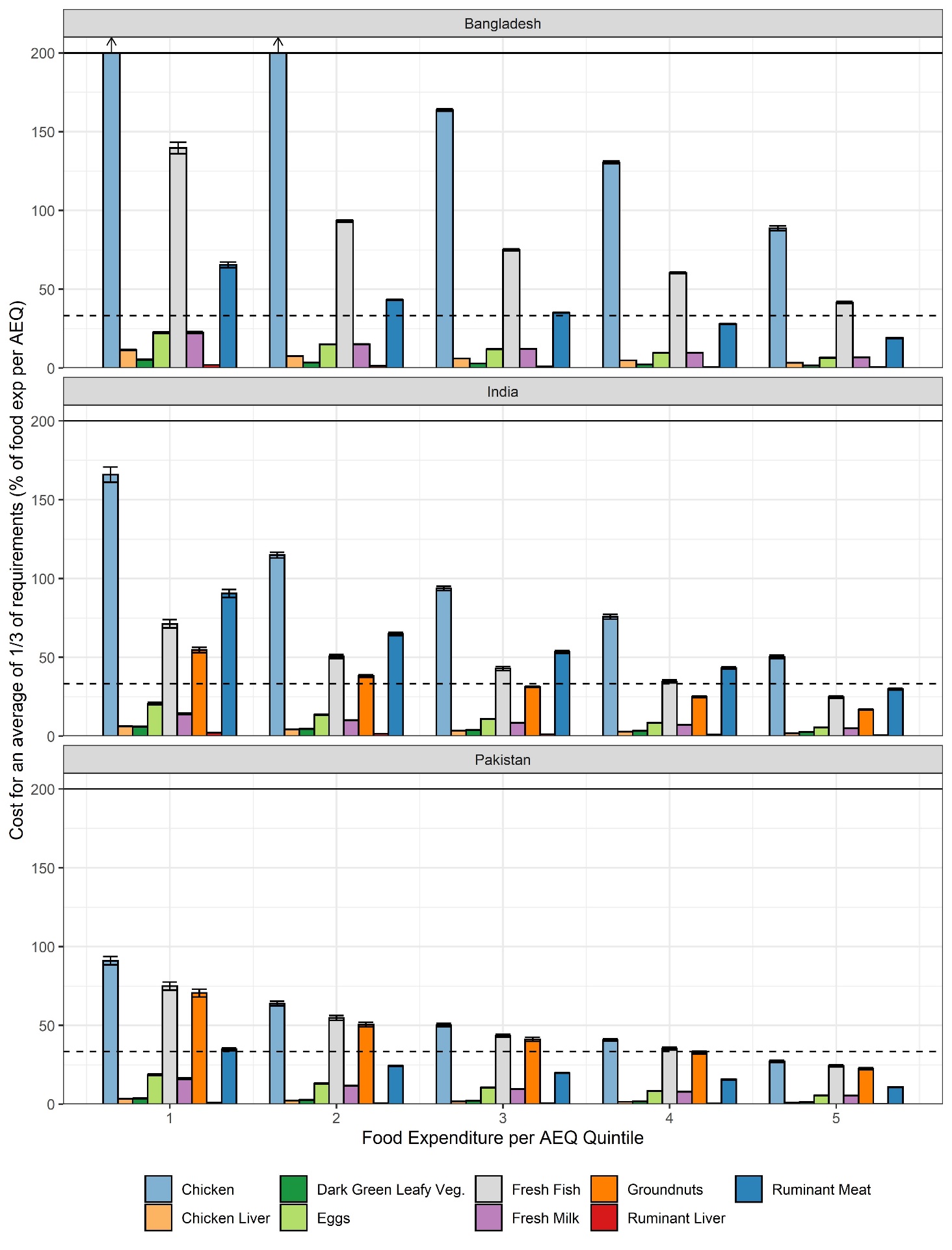


# Figure S26: Portion size cost, as a share of total household food expenditure per adult equivalent, using all surveyed households


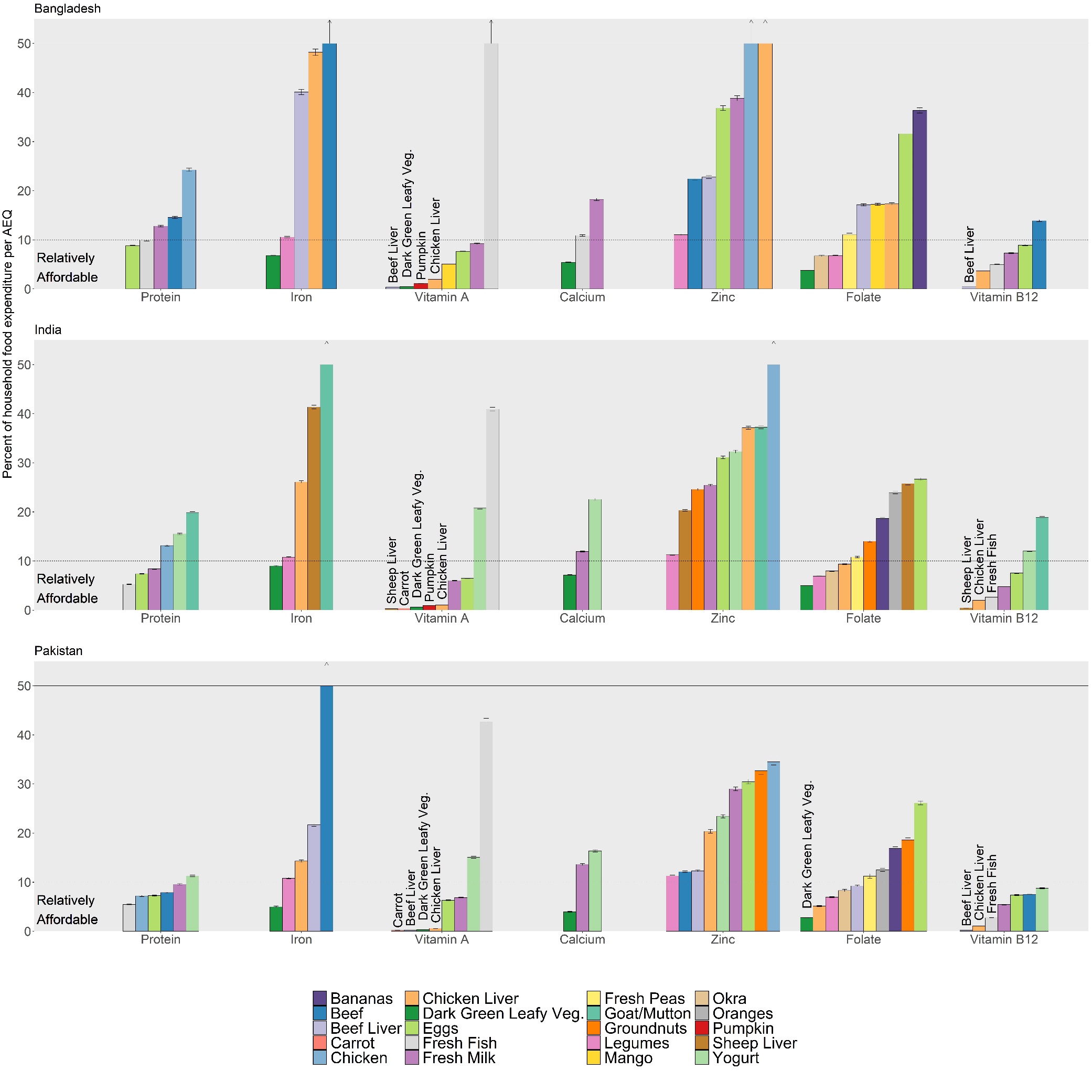


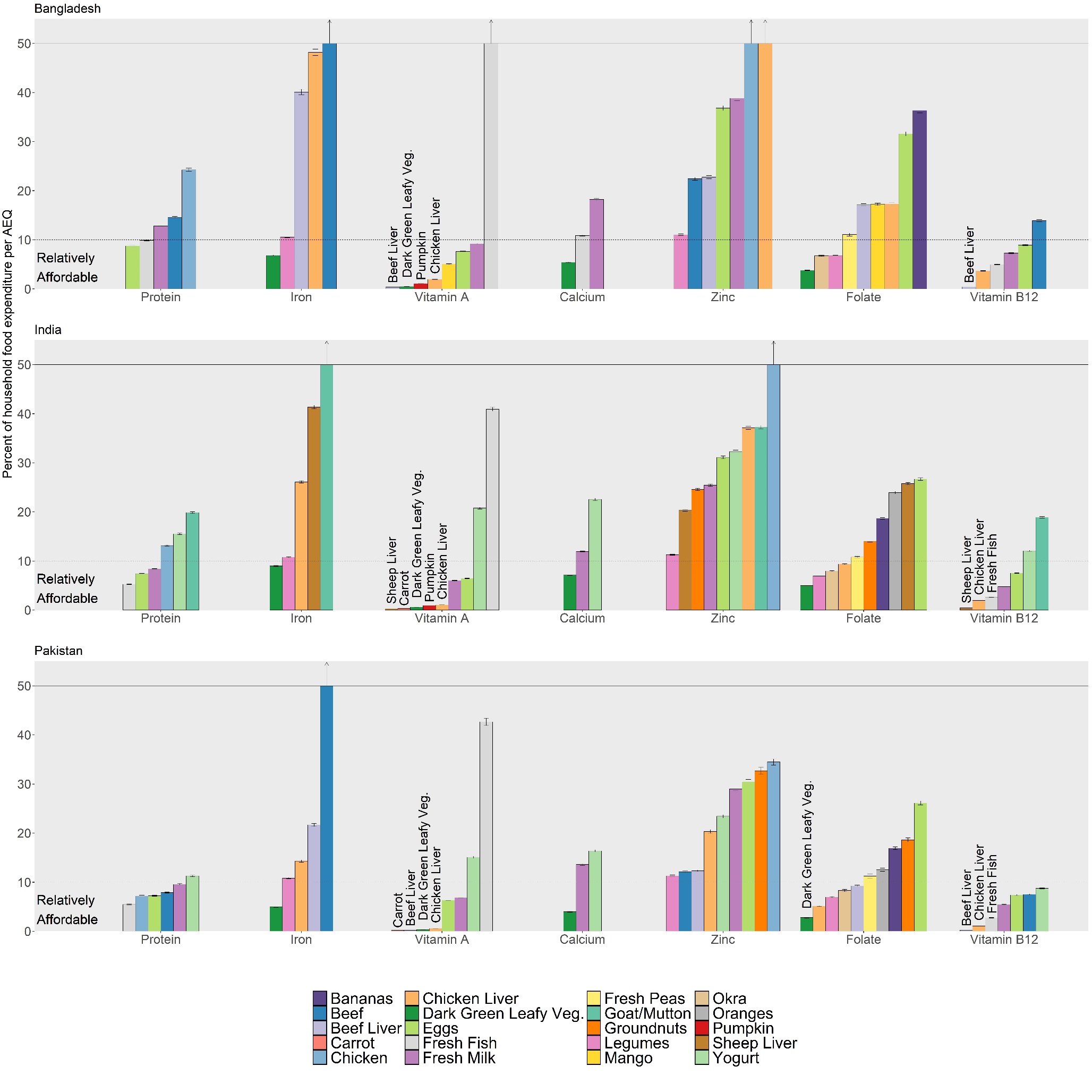


Note: The y-axis was truncated at 50, but the costs of some foods exceeded 50% of household food expenditure per AEQ; these foods are designated with vertical arrows indicating that the bar continues vertically beyond the scale of the graph. Sample sizes were 45,990 households (Bangladesh), 101,638 households (India), and 24,238 households (Pakistan). Error bars represent 95% confidence intervals.

# Figure S27A: Seasonal price variation by food and country – Bangladesh (taka)


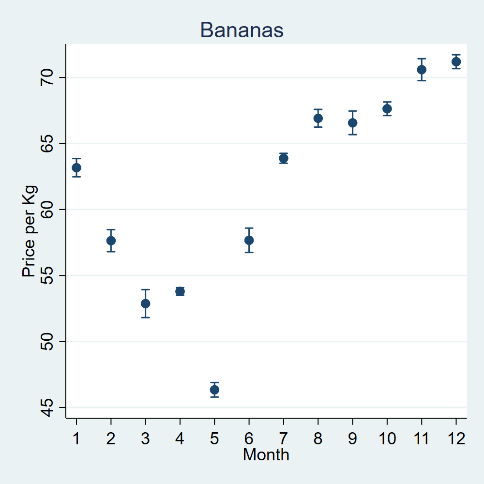

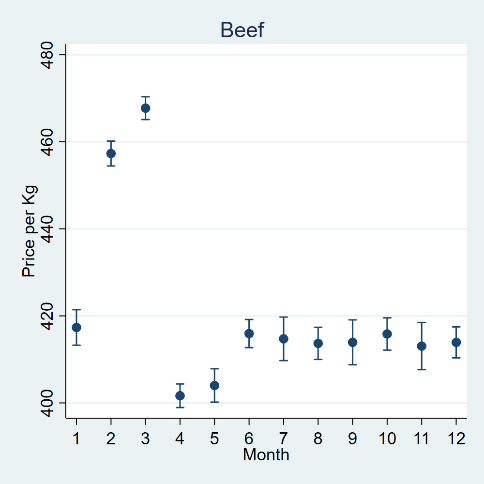

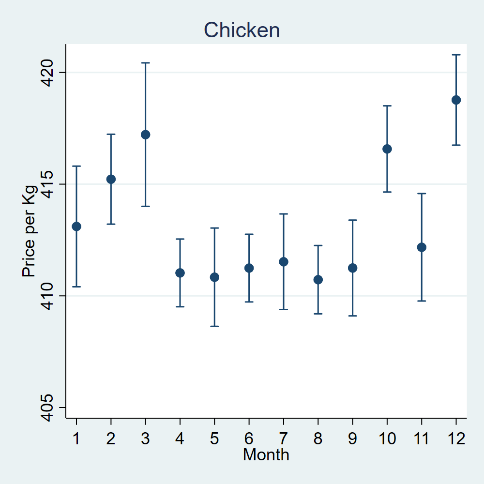

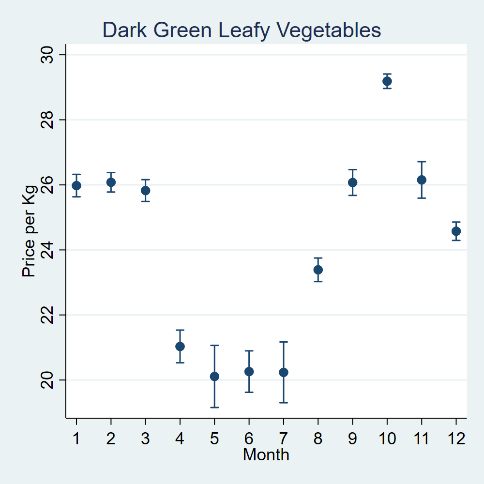

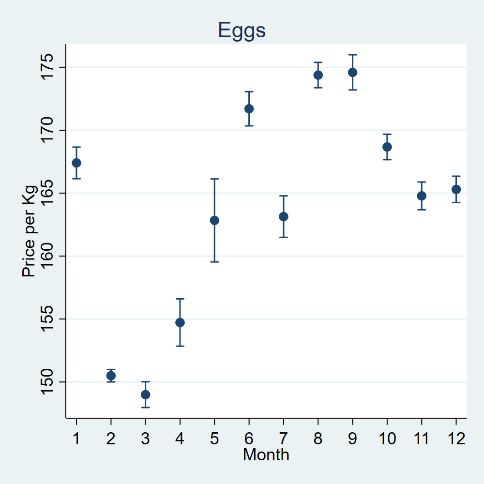

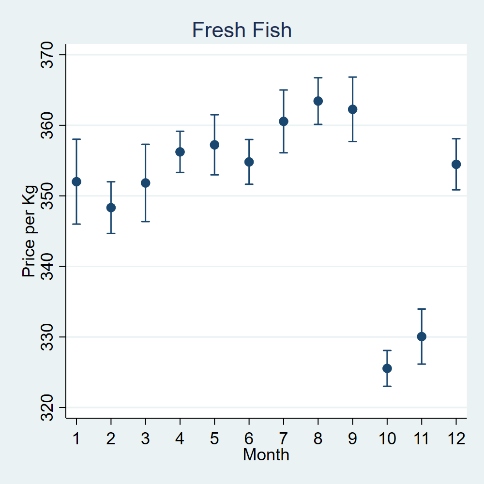

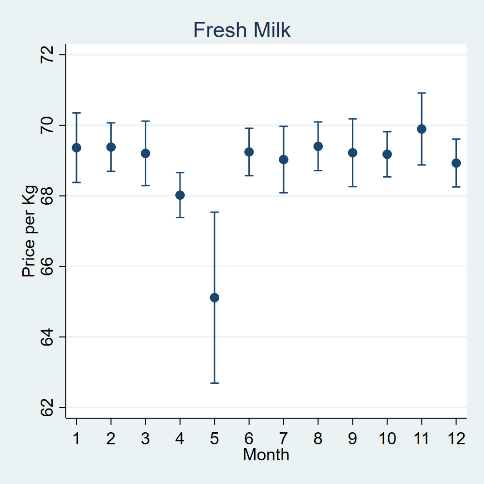

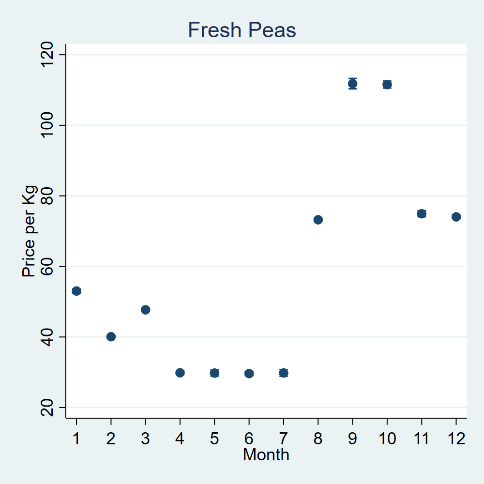

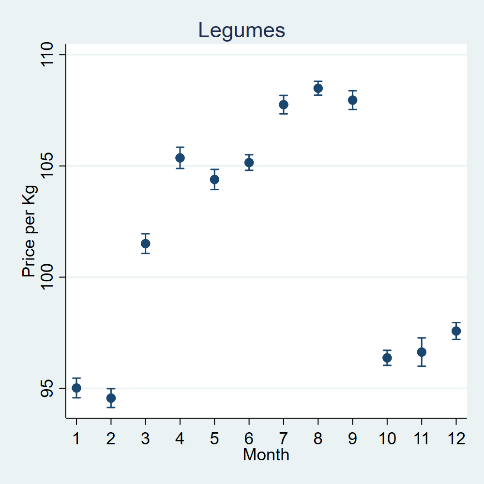


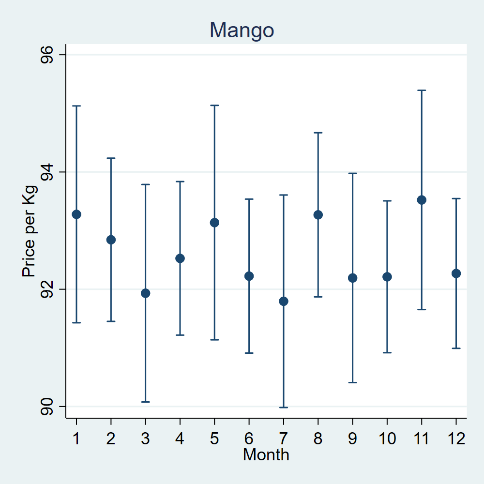

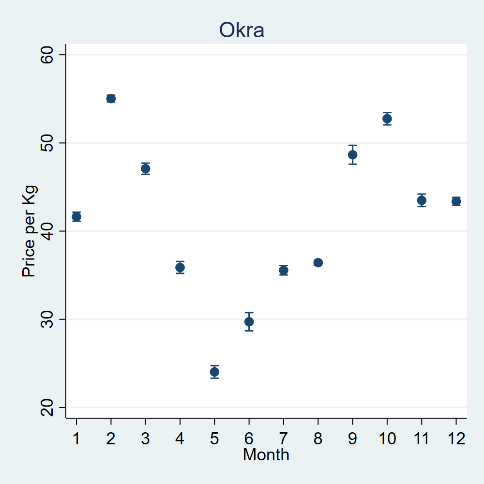

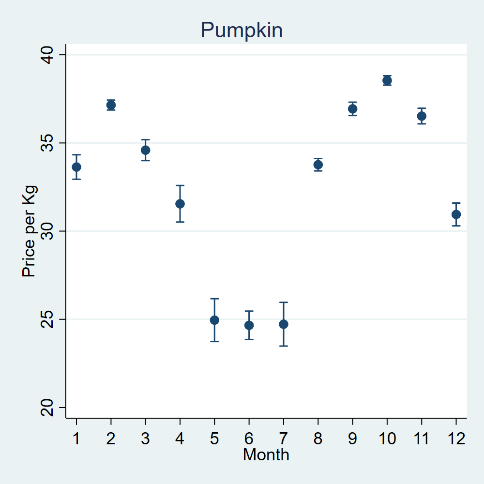


# Figure S27B: Seasonal price variation by food and country – India (rupees)


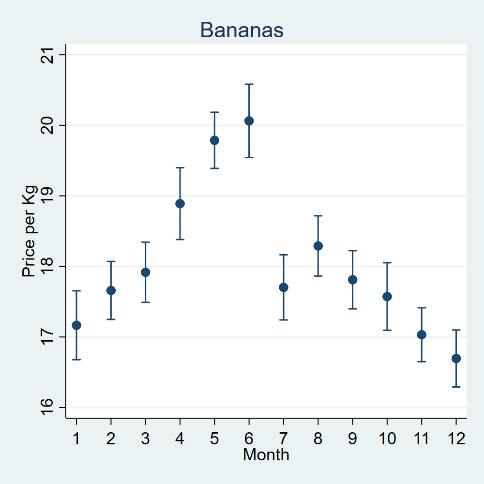

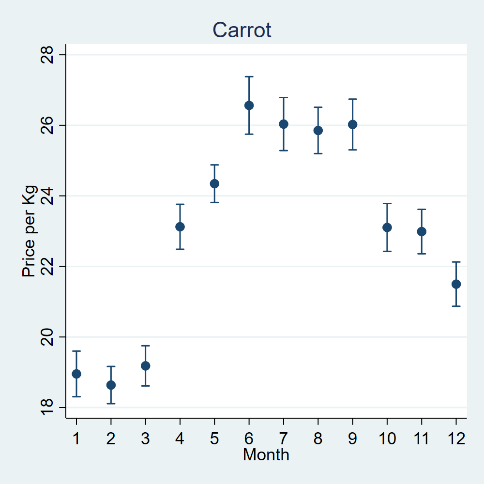

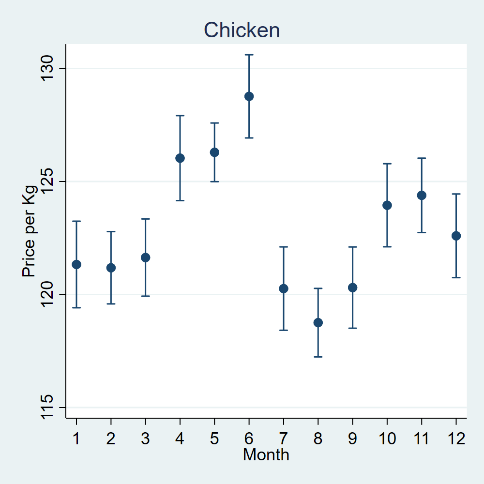


# Figure S27C: Seasonal price variation by food and country – Pakistan (rupees)

# References

1. *Bangladesh Annual Statistical Pocketbook*. Bangladesh Bureau of Statistics Accessed May 3, 2020. http://www.bbs.gov.bd/site/page/23b9eb9e-160e-4407-a49f-5722bc57f528/-

2. *Monthly Statisticaly Bulletins*. Bangladesh Bureau of Statistics Accessed May 3, 2020. http://www.bbs.gov.bd/site/page/62b0ad6f-ce62-499a-9ced-c7a93a980cd2/-

3. Food Prices. Accessed April 19, 2020. https://www.numbeo.com/food-prices/

4. U.S. Department of Agriculture, Agricultural Research Service. FoodData Central. Published 2019. Accessed January 26, 2020. https://fdc.nal.usda.gov./

5. Amalraj A, Pius A. Bioavailability of calcium and its absorption inhibitors in raw and cooked green leafy vegetables commonly consumed in India – An in vitro study. *Food Chemistry*. 2015;170:430-436. doi:10.1016/j.foodchem.2014.08.031

6. Shaheen N, Bari L, Mannan MA. Food Composition Table for Bangladesh. Published online 2013. http://www.fao.org/fileadmin/templates/food_composition/documents/FCT_10_2_14_final_version.pdf

7. Thilsted SH, Roos N, Hassan N. The role of small indigenous fish species in food and nutrition security in Bangladesh. *Naga, the ICLARM Quarterly*. 1997;20(3-4):82-84;102.

8. International Household Survey Network. Documentation: Bangladesh Household Income and Expenditure Survey 2016-17. Accessed September 9, 2020. https://catalog.ihsn.org/index.php/catalog/7399/related-materials

9. International Household Survey Network. Documentation: India National Sample Survey 2011-2012 (68th round) - Schedule 1.0 (Type 2) - Consumer Expenditure. Accessed September 9, 2020. https://catalog.ihsn.org/index.php/catalog/3282/related-materials

10. HIICS female Questionnaire (2015-16).pdf. Accessed September 9, 2020. http://www.pbs.gov.pk/sites/default/files//pslm/publications/pslm_microdata_2015_16_HIES/HIICS%20female%20Questionnaire%20%282015-16%29.pdf

11. Monthly Price Indices (Base Year 2015-16) | Pakistan Bureau of Statistics. Accessed April 19, 2020. http://www.pbs.gov.pk/cpi-nb

12. Institute of Medicine (US) Committee to Review Dietary Reference Intakes for Vitamin D and Calcium. *Dietary Reference Intakes for Calcium and Vitamin D*. (Ross AC, Taylor CL, Yaktine AL, Del Valle HB, eds.). National Academies Press (US); 2011. Accessed June 14, 2020. http://www.ncbi.nlm.nih.gov/books/NBK56070/

13. World Health Organization, Food and Agriculture Organization of the United Nations, eds. *Vitamin and Mineral Requirements in Human Nutrition*. 2nd ed. World Health Organization ; FAO; 2004. https://apps.who.int/iris/bitstream/handle/10665/42716/9241546123.pdf

14. Dewey KG. Nutrition, Growth, and Complementary Feeding of The Brestfed Infant. *Pediatric Clinics of North America*. 2001;48(1):87-104. doi:10.1016/S0031-3955(05)70287-X

15. Azahan E. Edible Component Parts of Broiler Chickens. *MARDI Research Bulletin*. 1984;12(1):153-156.

16. Bognar A. *Tables on Weight Yield of Food and Retention Factors of Food Constituents for the Calculation of Nutrient Composition of Cooked Foods (Dishes)*. Bundesforschungsanstalt für Ernährung; 2002. http://www.fao.org/uploads/media/bognar_bfe-r-02-03.pdf

17. Roseland JM, Nguyen QA, Williams JR, Patterson KY, Showell B, Pehrsson PR. USDA Table of Cooking Yields for Meat and Poultry. Published online 2014. https://data.nal.usda.gov/dataset/usda-table-cooking-yields-meat-and-poultry
